# Analysis of transition state mimicry by tight binding aminothiazoline inhibitors provides insight into catalysis by human *O*-GlcNAcase†
†Electronic supplementary information (ESI) available: Detailed experimental procedures, NMR spectra and additional figures illustrating *K*_i_ graphs, kinetic data, p*K*_a_ titration data and structural analysis data. See DOI: 10.1039/c6sc00370b


**DOI:** 10.1039/c6sc00370b

**Published:** 2016-02-15

**Authors:** N. Cekic, J. E. Heinonen, K. A. Stubbs, C. Roth, Y. He, A. J. Bennet, E. J. McEachern, G. J. Davies, D. J. Vocadlo

**Affiliations:** a Department of Chemistry , Simon Fraser University , Burnaby , British Columbia V5A 1S6 , Canada . Email: dvocadlo@sfu.ca; b School of Chemistry and Biochemistry , The University of Western Australia (M313) , 35 Stirling Highway , Crawley , WA 6009 , Australia; c York Structural Biology Laboratory , Department of Chemistry , The University of York , YO10 5DD , UK; d Department of Molecular Biology and Biochemistry , Simon Fraser University , Burnaby , British Columbia V5A 1S6 , Canada

## Abstract

The modification of nucleocytoplasmic proteins with *O*-linked *N*-acetylglucosamine (*O*-GlcNAc) plays diverse roles in multicellular organisms. Inhibitors of *O*-GlcNAc hydrolase (OGA), the enzyme that removes *O*-GlcNAc from proteins, lead to increased *O*-GlcNAc levels in cells and are seeing widespread adoption in the field as a research tool used in cells and *in vivo*. Here we synthesize and study a series of tight binding carbohydrate-based inhibitors of human OGA (hOGA). The most potent of these 2′-aminothiazolines binds with a sub-nanomolar *K*_i_ value to hOGA (510 ± 50 pM) and the most selective has greater than 1 800 000-fold selectivity for hOGA over mechanistically related human lysosomal β-hexosaminidase. Structural data of inhibitors in complex with an hOGA homologue reveals the basis for variation in binding among these compounds. Using linear free energy analyses, we show binding of these 2′-aminothiazoline inhibitors depends on the p*K*_a_ of the aminothiazoline ring system, revealing the protonation state of the inhibitor is a key driver of binding. Using series of inhibitors and synthetic substrates, we show that 2′-aminothiazoline inhibitors are transition state analogues of hOGA that bind to the enzyme up to 1-million fold more tightly than the substrate. These collective data support an oxazoline, rather than a protonated oxazolinium ion, intermediate being formed along the reaction pathway. Inhibitors from this series will prove generally useful tools for the study of *O*-GlcNAc. The new insights gained here, into the catalytic mechanism of hOGA and the fundamental drivers of potency and selectivity of OGA inhibitors, should enable tuning of hOGA inhibitors with desirable properties.

## Abstract 2

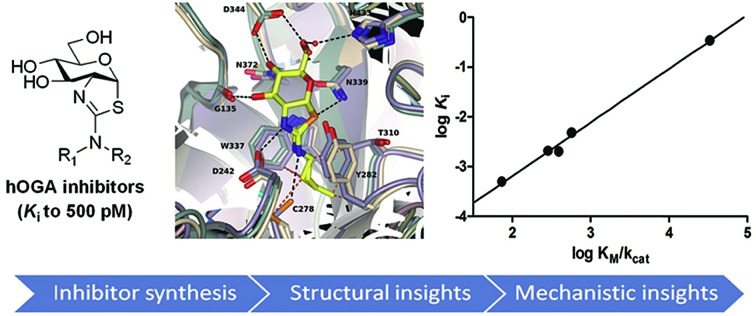
2′-Aminothiazoline inhibitors of human OGA are tight binding transition state mimics for which binding depends on inhibitor p*K*_a_.

## Introduction

The modification of serine and threonine residues of nuclear and cytoplasmic proteins with terminal *O*-linked β-*N*-acetylglucosamine (*O*-GlcNAc)^1^ has been found on hundreds of proteins.^2^–^4^ *O*-GlcNAc is present in all multi-cellular eukaryotes studied and occurs in a dynamic and reversible manner.^5^ Global *O*-GlcNAc levels have been shown to vary in response to cellular nutrient availability and stress and in some cases has been found to influence protein phosphorylation.^6^ These observations have stimulated interest in the physiological roles played by *O*-GlcNAc and research has implicated this modification in controlling various cellular processes including, for example, proteosomal degradation of proteins^7^–^9^ and transcriptional regulation.^10^,^11^ Additionally, a growing body of literature has implicated *O*-GlcNAcylation in chronic diseases such as neurodegeneration^12^–^15^ and cancer.^9^,^16^,^17^ Given the mounting potential in targeting protein *O*-GlcNAcylation for therapeutic benefit, there has been rising interest in understanding the molecular basis for inhibition of modulators of the *O*-GlcNAc pathway and the creation of small molecule modulators of this pathway for use in tissues.^18^–^20^

The glycosyltransferase uridine diphospho-*N*-acetylglucosamine:peptide β-*N*-acetylglucosaminyl transferase (OGT) installs *O*-GlcNAc residues using uridine diphosphate *N*-acetylglucosamine (UDP-GlcNAc) as the sugar substrate donor.^21^,^22^ The enzyme responsible for removing *O*-GlcNAc from proteins is *O*-GlcNAcase (OGA),^23^ which is a member of glycoside hydrolase family 84 (GH84) of the CAZy classification system.^24^ Consistent with the reversible nature of protein *O*-GlcNAcylation, inhibitors of these enzymes have been shown to induce time-dependent changes in cellular *O*-GlcNAc levels.^19^,^25^–^27^ Small molecule inhibitors of OGA, in particular, have emerged as commonly used research tools for evaluating the phenotypic effects of increased *O*-GlcNAc levels in cultured cells, as well as *in vivo*.

**Fig. 1:**
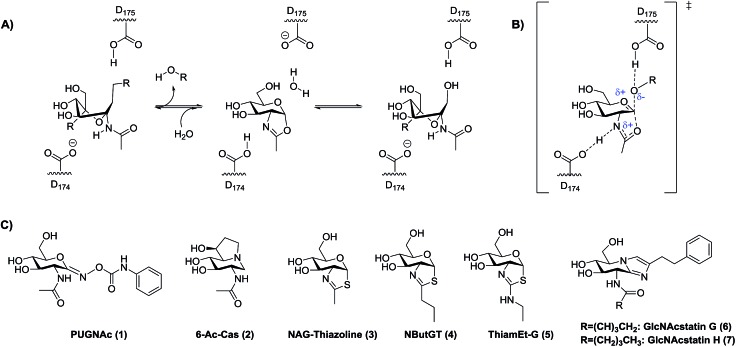
Catalytic mechanism of OGA and lysosomal β-hexosaminidases and some known hOGA inhibitors. (A) hOGA uses a substrate-assisted catalytic mechanism involving two key catalytic aspartate residues that enable the transient formation of an oxazoline intermediate. R = leaving group. (B) The proposed transition state (TS) for formation of the oxazoline intermediate. Note that the extent of proton transfer to the stabilizing residue D_174_/D_354_ is not known. (C) Some known hOGA inhibitors. Catalytic residues are D_174_/D_175_ for human OGA and D_354_/E_355_ for human lysosomal β-hexosaminidase A.

**Fig. 2:**
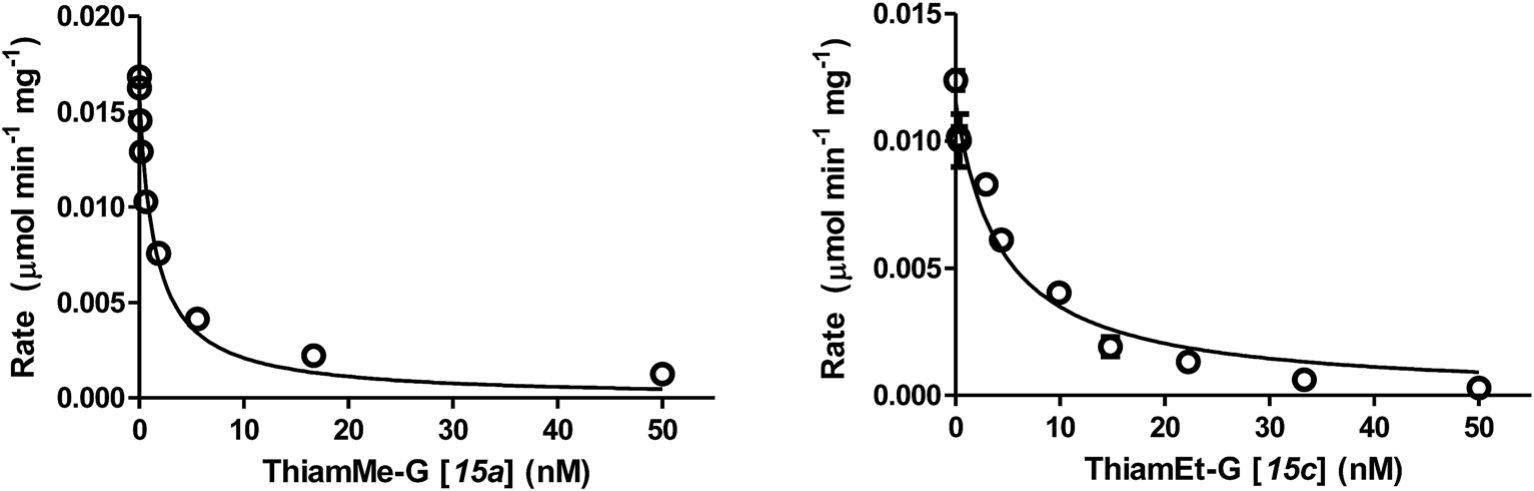
Representative Morrison data and fitted curves used to obtain *K*_i_ values for the tight binding hOGA inhibitors, shown for ThiamMe-G (**15a**) and ThiamEt-G (**15c**). Data was obtained in triplicate and error bars represent the standard error of the mean (S.E.M.).

**Fig. 3:**
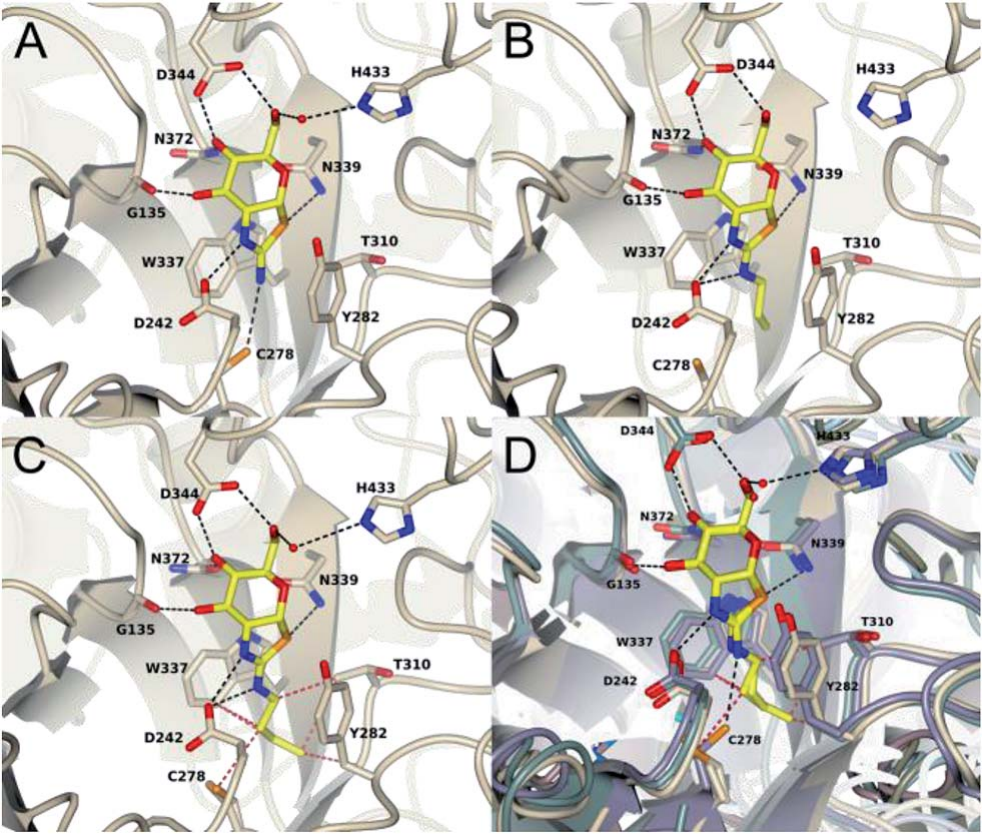
Cartoon plots of *Bt*GH84 in complex with **11a** (A), **11b** (B) and **15e** (C). The inhibitors are shown in stick representation. The side chains of the residues forming the active site are shown in stick representation. Hydrogen bonds between the protein and the inhibitor are shown as black dashed lines. Close contacts (*d* < 3.4 Å) of butylaminothiazoline (C) are shown as red dashed lines. (D) shows an overlay of all three structures based on the bound aminothiazoline. The respective protein environment is coloured in darkblue for aminothiazoline containing structure, for propyleneaminothiazoline in bluegreen and butylaminothiazoline in beige. A change of the rotamer of C278 and a shift of the corresponding β-strand is observed in response to the steric repulsion with the butyl substituent.

Interest in inhibitors of OGA has gained increasing attention due to growing recognition of the physiological roles of *O*-GlcNAc. Among the first reported inhibitors of OGA is *O*-(2-acetamido-2-deoxy-d-gluco-pyranosylidene)amino-*N*-phenylcarbamate (PUGNAc, Fig. 1, **1**)^28^ (hOGA *K*_i_ = 46 nM). This inhibitor, however, has well-described off target effects^29^,^30^ including the inhibition of the functionally related lysosomal β-hexosaminidases HEXA and HEXB from family GH20.^26^ These two lysosomal enzymes cleave β-linked terminal *N*-acetylhexosamine residues from various glycoconjugates including gangliosides. Genetic deficiency of these hexosaminidases results in Tay–Sachs and Sandhoff's disease, which stem from the accumulation of gangliosides within lysosomes. More recently identified inhibitors such as 6-acetamido-6-deoxy-castanospermine (6-Ac-Cas)^29^ (hOGA *K*_i_ = 300 nM) (Fig. 1, **2**), and 1,2-dideoxy-2′-methyl-α-d-glucopyranoso-[2,1-*d*]-Δ2′-thiazoline (NAG-thiazoline)^26^ (hOGA *K*_i_ = 70 nM) (Fig. 1, **3**) while fairly potent, are also non-selective. Given that gangliosides play varied roles in cellular processes ranging from cell membrane structure to cell signaling, the selectivity of OGA inhibitors has emerged as being important for the creation of useful probe molecules for use *in vivo*.^29^,^31^,^32^

**Fig. 4:**
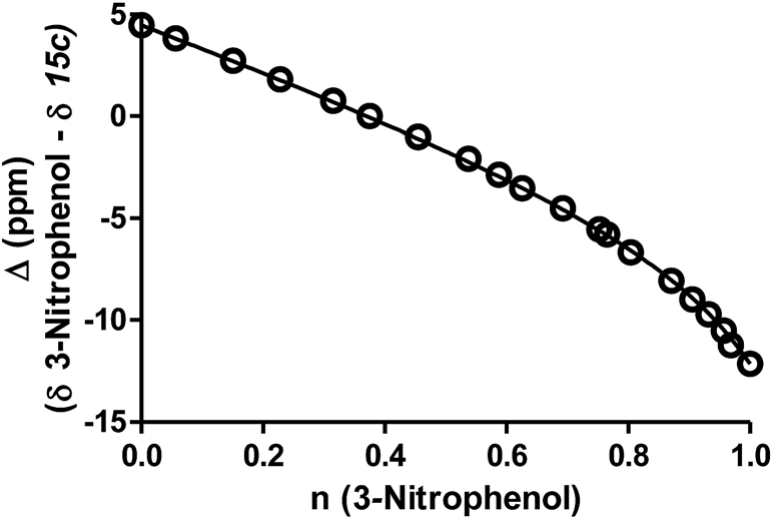
A representative example for ^13^C-NMR determination of the p*K*_a_ value for ThiamEt-G (**15c**) with chemical shifts of the 2′-C of the thiazoline ring resonances (*Δ* ppm) for compounds **15c** as a function of the fractional protonation (*n*) of the reference compound, 3-nitrophenol (p*K*_a_ of 8.42). The solid line is the best non-linear least squares fit to eqn (S1) (ESI†).

Rationally designed OGA inhibitors have been pursued based on knowledge of the catalytic mechanism of hOGA. Detailed mechanistic studies^26^,^33^ coupled with structural studies of bacterial homologues of hOGA^34^,^35^ have provided clear support for a catalytic mechanism involving substrate-assisted catalysis in which the 2-acetamido group of the substrate serves as a catalytic nucleophile to generate a transient enzyme-bound oxazoline or oxazolinium intermediate (Fig. 1A) and stabilizing an oxocarbenium ion-like transition state (Fig. 1B). In this two-step catalytic mechanism, two aspartates (Asp^174^ and Asp^175^) play key roles as general acid/base catalytic residues.^33^ Asp^174^ serves to orient and polarize the 2-acetamido group to aid its attack at the anomeric center, accepting a proton during formation of the oxazoline ring. Asp^175^ acts as a general acid, donating a proton to the glycosidic oxygen during cleavage of the glycosidic bond (Fig. 1A and B). Given the clear resemblance of NAG-thiazoline to the oxazoline intermediate, analogues of this molecule in which the 2′-position of the thiazoline ring is modified showed fair selectivities coupled with moderate nanomolar potencies as exemplified by NButGT (hOGA *K*_i_ = 230 nM, *K*_i_(HEX)/*K*_i_(hOGA) = 1,500, Fig. 1C, **4**).^26^

**Fig. 5:**
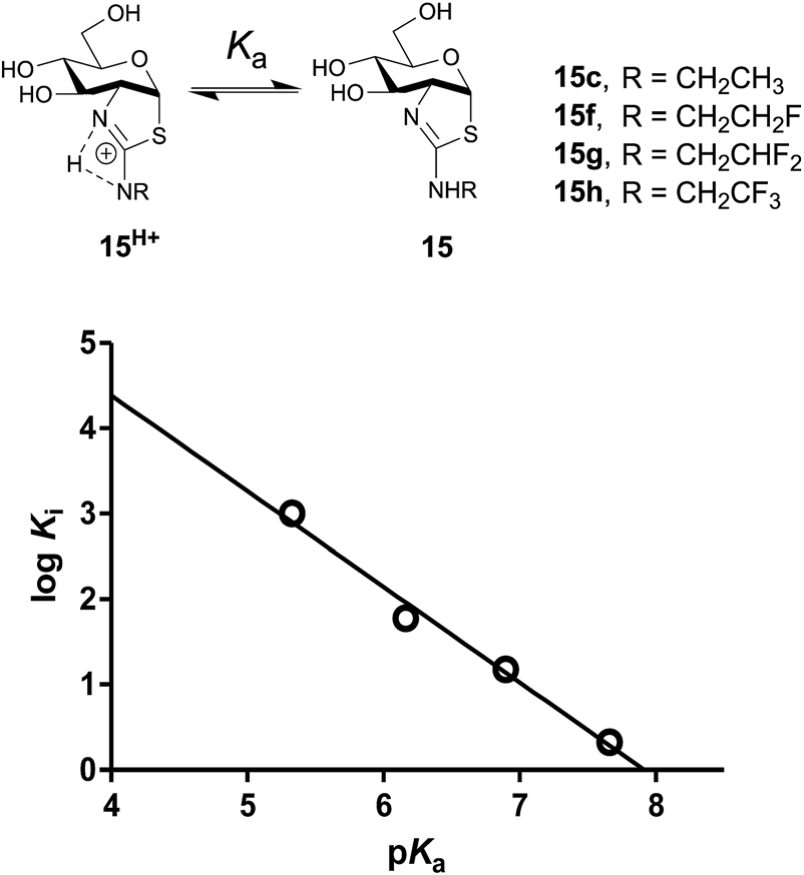
Acid dissociation of the 2′-aminothiazolium protonation state of 2′-aminothiazoline inhibitors **15c** and **15f–h** and the linear free energy relationship (LFER) analysis between the p*K*_a_ and log *K*_i_. Of these inhibitors shows a negative correlation of –1.12 ± 0.09.

More selective hOGA inhibitors have since been generated including the GlcNAcstatins^27^,^36^ such as GlcNAcstatin C (hOGA *K*_i_ = 3.2 nM, *K*_i_(HEX)/*K*_i_(hOGA) = 190) and a bioisostere of NButGT, 1,2-dideoxy-2′-ethylamino-α-d-glucopyranoso-[2,1-*d*]-Δ2′-thiazoline (ThiamEt-G)(hOGA *K*_i_ = 21 nM, *K*_i_(HEX)/*K*_i_(hOGA) = 37 000)^19^ (Fig. 1C, **5**), which is more synthetically accessible. ThiamEt-G is orally available and increases brain *O*-GlcNAc levels in mammals and has been used by several groups to show chronic OGA inhibition and increased *O*-GlcNAcylation over several months has no apparent deleterious effects and also protects in various mouse models of AD against both tau^14^,^37^,^38^ and amyloid pathologies.^39^,^40^ Given the great interest in OGA inhibitors as research tools, we aimed to explore the basis for the inhibition of hOGA by ThiamEt-G. Here we report on the synthesis and characterization of aminothiazoline inhibitors with hOGA and human HexB (hHexB), demonstrate the strongly p*K*_a_ dependent inhibition of hOGA by such inhibitors, illuminate the molecular basis for observed selectivity and potency using structural biology, and reveal these inhibitors are genuine TS analogues – which reveals new insight into the nature of the catalytic mechanism as well as explaining the picomolar binding of the best representative from this inhibitor family.

## Results and discussion

**Fig. 6:**
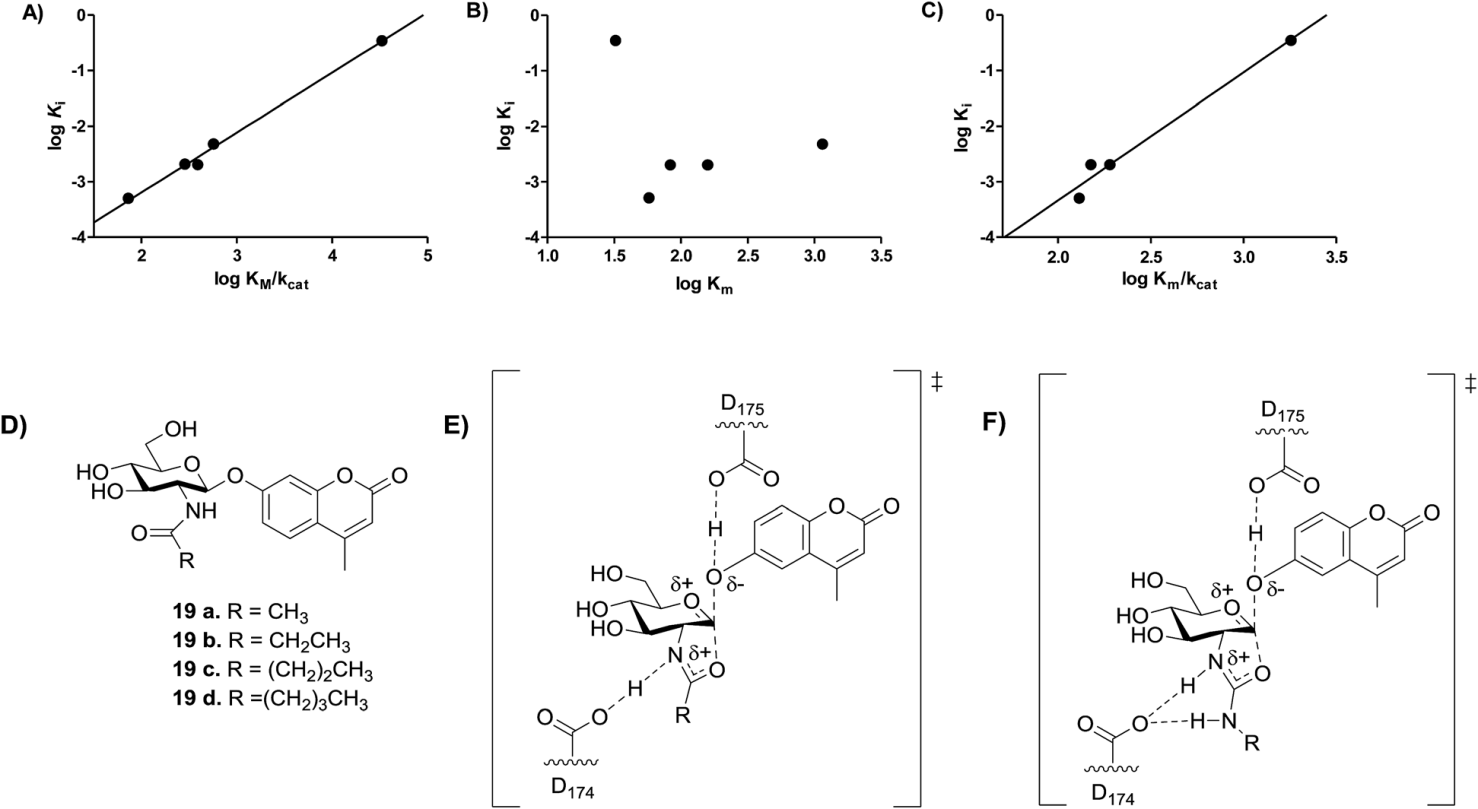
Analysis of transition state analogy for hOGA 2′-aminothiazoline inhibitors (**11a**, **15a**, **15c–e**) and urea (**18a–e**) and acyl (**19a–e**) substrates. (A) The correlation between log *K*_m_/*k*_cat_ of substrates **18a–e** and log *K*_i_ of inhibitors **11a**, **15a** and **15c–e** (*m* = 1.08 ± 0.04, *R*^2^ = 0.9950). (B) The correlation between the log *K*_m_ of substrates **18a–e** and log *K*_i_ of inhibitors **11a**, **15a**, **15c–e**. (C) The correlation between the log *K*_m_/*k*_cat_ of substrates **19a–d** and log *K*_i_ of inhibitors **15a** and **15c–e** (*m* = 2.31 ± 0.25, *R*^2^ = 0.9768). (D) Substrates **19a–d** synthesized by Whitworth *et al.*^51^ (E) Transition state for the hOGA catalyzed hydrolysis of substrates **19a–d** likely involves transfer of the amidic proton. (F) Transition state for the hOGA catalyzed hydrolysis of substrates **18a–e** likely involves no transfer of the amidic proton.

Early studies showed that varying the 2′-alkyl substituent of NAG-thiazoline resulted in increased selectivity for OGA over the lysosomal hexosaminidases at the slight expense of potency.^26^ This trend does not hold for PUGNAc analogues which show only modest selectivity.^41^,^42^ Exploiting a similar approach of increasing steric bulk of the acetamido group, however, cell-penetrant glucoimidazole inhibitors including the hOGA inhibitors GlcNAcstatin G (hOGA = 4.1 nM, *K*_i_(HEX)/*K*_i_(hOGA) = 900 000) (Fig. 1C, **6**) and GlcNAcstatin H (hOGA = 2.6 nM, *K*_i_(HEX)/*K*_i_(hOGA) = 35 000) (Fig. 1C, **7**), have been uncovered as some of the most selective hOGA inhibitors to date.^20^ Structural studies have suggested that these OGA inhibitors derive their selectivity from structural differences between the active sites of OGA and the lysosomal β-hexosaminidases in the region that serves to position the 2-acetamido group of the substrate.^34^,^43^ We therefore first set out to gain an understanding of the detailed relationships between the size of substituents at the 2′-position of a series of 2′-aminothiazoline inhibitors and the influence on binding of altered electronic properties of the 2′-aminothiazoline system.

### Preparation, potency, and selectivity of 2′-alkylaminothiazoline OGA inhibitors

**Scheme 1:**
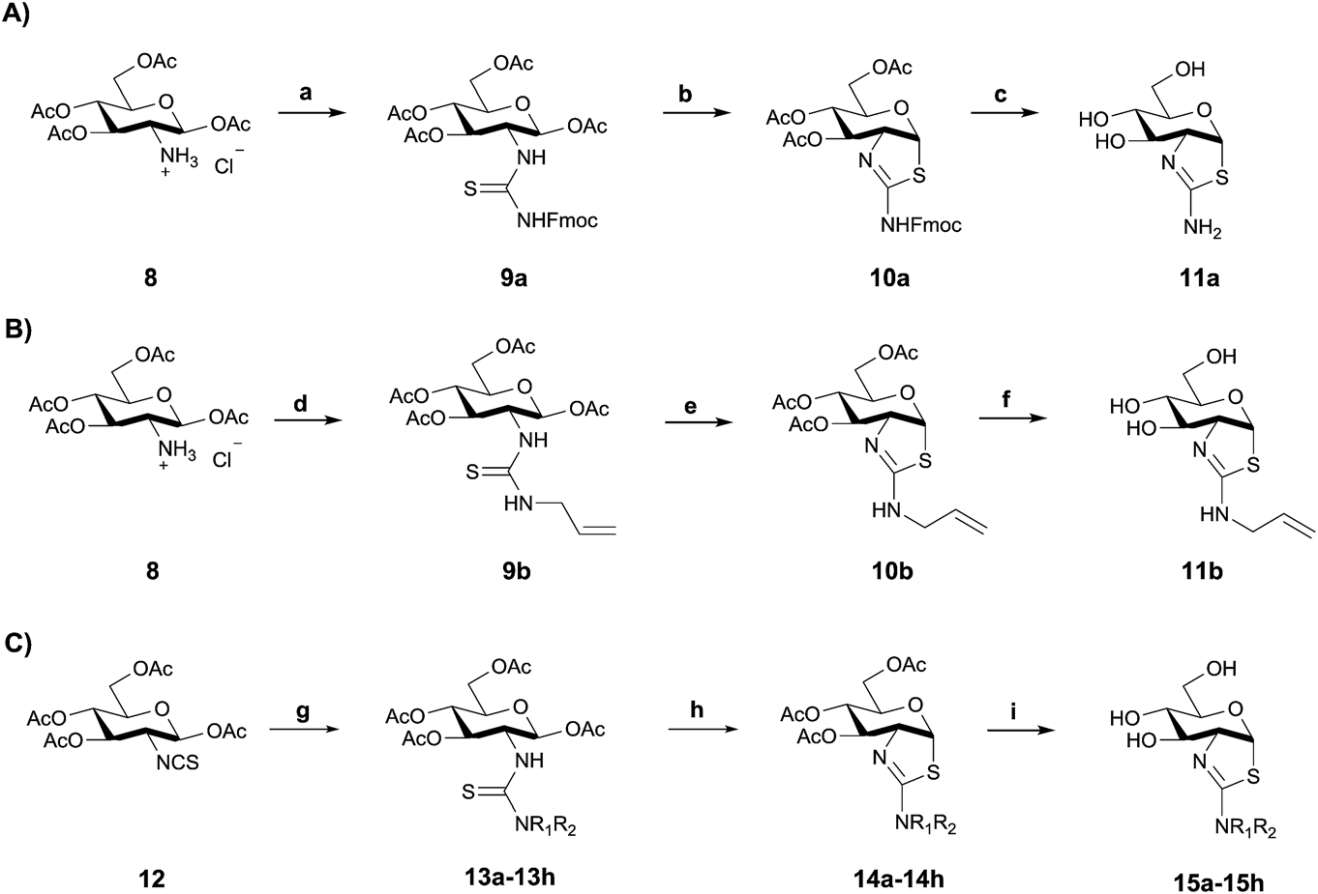
Synthesis of OGA inhibitors **11a–b** and **15a–h** from **8** and **12**, respectively. (A) **11a**: (a) 1. NEt_3_, DCM; 2. Fmoc-NCS, pyridine, NEt_3_; (b) SnCl_4_, pyridine, NEt_3_; (c) 1. (i) NaOMe, MeOH; (ii) AcOH; 2. piperidine, DMF; (B) **11b**: (d) allyl-NCS (2 eq.), NEt_3_ (2 eq.), CH_3_CN; (e) TFA (7.5 eq.), DCM; (f) K_2_CO_3_, MeOH; (C) **15a–h**: (g) NHR_1_R_2_·HCl (1.2 eq.), NEt_3_ (1.2 eq.), CH_3_CN; (h) TFA (7.5 eq.), DCM (i) K_2_CO_3_, MeOH; **13a–15a**: R_1_ = H, R_2_ = CH_3_; **13b–15b**: R_1_ = CH_3_, R_2_ = CH_3_; **13c–15c**: R_1_ = H, R_2_ = CH_2_CH_3_; **13d–15d**: R_1_ = H, R_2_ = (CH_2_)_2_CH_3_; **13e–15e**: R_1_ = H, R_2_ = (CH_2_)_3_CH_3_; **13f–15f**: R_1_ = H, R_2_ = (CH_2_)_2_F; **13g–15g**: R_1_ = H, R_2_ = CH_2_CHF_2_; **13h–15h**: R_1_ = H, R_2_ = CH_2_CF_3_.

Using three different approaches (Scheme 1) we synthesized a series of 2′-alkylaminothiazoline derivatives. Using the common intermediate hydrochloride salt of 1,3,4,6-tetra-*O*-acetyl-2-amino-2-deoxy-β-d-glucopyranose **8** (Scheme 1A and B), which was conveniently accessed in three steps,^44^ we prepared compounds **11a** and **11b** by reacting either *N*-fluorenylmethyloxycarbonyl (Fmoc)-protected isothiocyanate or *N*-allyl isothiocyanate with **8** in the presence of triethylamine to generate the respective thiourea intermediates **9a** and **9b**. Subsequent cyclization with excess SnCl_4_ followed by a two step deprotection using catalytic NaOMe in anhydrous methanol, and piperidine catalyzed removal of the Fmoc group, afforded analogue **11a**. Cyclization of thiourea **9b** with excess TFA, followed by a one step deprotection using catalytic K_2_CO_3_ in anhydrous methanol afforded inhibitor **11b**.

Inhibitors **15a–h** were synthesized using an alternate route from the common isothiocyanate intermediate 1,3,4,6-tetra-*O*-acetyl-2-deoxy-2-isothiocyanato-β-d-glucopyranose (Scheme 1C, **12**), which was prepared from **8***via* a biphasic reaction in H_2_O/DCM with thiophosgene.^44^ Reaction of isothiocyanate **12** with a series of alkylamines and dialkylamines, or their respective hydrochloride salts, yielded thioureas **13a–h**. Acid catalyzed cyclization of these thiourea-containing compounds using TFA provided protected aminothiazolines **14a–h**, which after deprotection with K_2_CO_3_ afforded **15a–h**.

We then determined the *K*_i_ values for inhibition of hOGA by compounds **11a–b** and **15a–h**. Using Michael–Menten kinetics we obtained the *K*_i_ values for the less potent inhibitors **15e–h**, and as expected, double-reciprocal Lineweaver–Burk plots revealing a competitive mode of inhibition (ESI Fig. S1†). For the increasingly tight binding inhibitors we used the non-linear fitting method described by Morrison,^45^ which can be used to determine *K*_i_ values which are comparable to the concentration of the enzyme being studied. Refinement of the Morrison approach by Copeland enables defining the *K*_i_ value for an inhibitor through the use of relative rates using a quadratic equation (eqn (1)).^46^ This approach depends on knowing the initial free enzyme and inhibitor concentrations without the assumption that the free inhibitor concentration is equal to the total inhibitor concentration.1
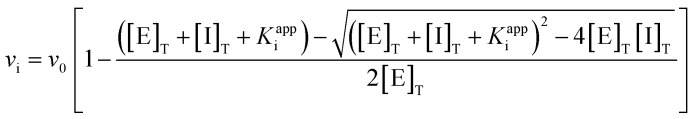



Further, *K*_i_ values can be accurately determined over a wide range of enzyme concentrations using this method,^47^ which enabled us to use hOGA concentrations that permit accurate initial rate determinations. We followed existing guidance^48^ to select inhibitor concentrations for our *K*_i_ value determinations and confirmed the accuracy of this method by showing the *K*_i_ values for inhibitor **11b** were in reasonable accord when using either the Michaelis–Menten or Morrison method (ESI Fig. S2†).

Using these methods we found the *K*_i_ values for inhibition of hOGA by compounds **11a–b** and **15a–h** ranged from the high sub-nanomolar to low nanomolar range (Table 1). Notably, we find that the *K*_i_ value for ThiamEt-G (Fig. 1 and 2) was 10-fold lower (*K*_i_ = 2.1 nM) than that previously determined^19^ using the Michaelis–Menten method (*K*_i_ = 21 nM). This makes Thiamet-G over 100-fold more potent than the isosteric NButGT (*K*_i_ = 230 nM). The most tight-binding compound with a *K*_i_ of 510 ± 50 pM (Fig. 2) is **15a** (ThiamMe-G, Scheme 1), which ranks this compound among the most potent glycoside hydrolase inhibitors known, as well as the most potent selective hOGA inhibitor reported. Interestingly, we note that there is only a slight decrease in potency for hOGA upon increasing the volume of the 2′-aminoalkyl substituent to the point where the alkyl group is a propyl (**15d**, *K*_i_ = 2 nM). A butyl chain, however, leads to a greater than 100-fold loss of potency (**15e**, *K*_i_ = 350 nM). Structures of bacterial OGA homologues, in which the active site residues are completely conserved with hOGA,^34^,^35^ show a discretely sized pocket having a volume that nicely accommodates the propyl substituent of **15d**.

**Table 1:** *K*_*i*_ selectivity ratios of inhibitors **11a** to **15h** for hOGA over hHexB and the p*K*_a_ values for **15c**, **15f–h**

Inhibitor	hOGA *K*_i_^*a*^ (nM)	hHexB *K*_i_^*b*^ (μM)	(hHexB/hOGA)^*c*^	p*K*_a_^*e*^	Fraction protonated at pH 7.4
**11a**: R_1_ = R_2_ = H	4.7 ± 0.3	5.0 ± 0.6^*d*^	1100		
**15a**: R_1_ = H, R_2_ = CH_3_	0.51 ± 0.05	1.7 ± 0.19^*d*^	3300		
**15b**: R_1_ = R_2_ = CH_3_	2.4 ± 0.2	13.0 ± 3.8	5400		
**15c**: R_1_ = H, R_2_ = CH_2_CH_3_	2.1 ± 0.3	740 ± 60 (ref. 19)	350 000	7.68	0.66
**11b**: R_1_ = H, R_2_ = CH_2_CHCH_2_	3.2 ± 0.4	2850 ± 570	950 000		
**15d**: R_1_ = H, R_2_= (CH_2_)_2_CH_3_	2.0 ± 0.2	3700 ± 670	1 850 000		
**15e**: R_1_ = H, R_2_= (CH_2_)_3_CH_3_	350 ± 90^*d*^	4800 ± 763	13 700		
**15f**: R_1_ = H, R_2_= (CH_2_)_2_F	15 ± 5^*d*^	180 ± 44	12 000	6.92	0.25
**15g**: R_1_ = H, R_2_ = CH_2_CHF_2_	60 ± 10^*d*^	150 ± 50	2500	6.18	0.06
**15h**: R_1_ = H, R_2_ = CH_2_CF_3_	1000 ± 200^*d*^	4200 ± 1525	4200	5.33	0.01

^*a*^Determined using the Morrison *K*_i_ fit if the values are below 5 mM.

^*b*^Determined using Dixon plot analysis.

^*c*^Selectivity ratios representing the favored selectivity for hOGA compared to hHexB.

^*d*^Determined using Michaelis–Menten inhibition analysis.

^*e*^See ESI for full details, p*K*_a_ (NButGT) = 4.65.

To assess the structural basis for this stepped decrease in potency observed on going to the 2′-aminobutylthiazoline **15e**, we determined the structure of *Bacteroides thetaiotaomicron*, a bacterial homolog (*Bt*GH84) of hOGA, in complex with the parent 2′-aminothiazoline **11a**, the tighter binding 2′-aminopropylenethiazoline **11b**, and the butyl derivative **15e**, which shows greatly diminished binding. In all three structures, the respective inhibitor binds in the active site in a conserved mode with an invariant hydrogen bond pattern for the aminothiazoline moiety (Fig. 3). The alkyl chain of the modified aminothiazolines points into the conserved hydrophobic pocket as shown for ThiamEt-G.^19^ An inspection of the binding pocket shows no further hydrophobic interactions in the case of the unsubstituted aminothiazoline **11a**, except a possible weak electrostatic interaction with C278 at the bottom of the pocket (Fig. 3A). Propylene derivative **11b** matches the size of the pocket, requiring only a minor adjustment of the C278 rotamer and showing favorable hydrophobic interactions with W337, T310, and Y282 (Fig. 3B). Even a small further extension of the alkyl chain, as in the butyl derivative **15e**, leads to steric clashes with multiple residues in the cavity, driving the side chain of C278 to adopt a different orientation. Furthermore, we observe the polypeptide main chain surrounding C278 is shifted away from the inhibitor. Thus, the size of the pocket disfavors substituents longer than three carbon units in chain length, and those inhibitors having such larger groups induce unfavorable conformational changes within the active site (Fig. 3C and D).

We next set out to assess the selectivity of this series of inhibitors for hOGA over the lysosomal β-hexosaminidases, which are comprised of combinations of α and β subunits that are products of the highly homologous HEXA and HEXB genes. Using purified human hexosaminidase B (hHexB) we determined the approximate *K*_i_ values for compounds **11a** to **15h** using Dixon plot analysis and found remarkably high inhibitor selectivities ranging from 1100- to 1 850 000-fold preference for hOGA (Table 1). We confirmed these Dixon plot analyses for the two most potent compounds, **11a** and **15a**, by determining full *K*_i_ values for their inhibition of hHexB (ESI Fig. S4†). Remarkably, inhibitors **11a** and **15a** still retain 1100 and 3300-fold selectivity for hOGA despite their similarity in size to NAG-thiazoline, which itself demonstrated no selectivity.^26^ Accordingly, the presence of the 2′-amino substituent, on its own, confers at least 1000-fold selectivity for hOGA over hHexB.

We also noted that the selectivity ratio for hOGA increases as the 2′-aminoalkyl chain length increases to the three-carbon propyl (**15d**) and propylene (**11b**) derivatives, but this trend reverses once the chain length increases further, as seen for the 2′-aminobutyl analogue (**15e**). Since the active site pocket for hHexB is more constrained in the vicinity of the acetamido group, these observations are consistent with structural observations of both bacterial hOGA homologues^34^ and hHexB.^43^ This suggests that once the 2′-substituent passes the volume that can be accommodated in the active site of hOGA, increases in bulk are even slightly more deleterious for hOGA as compared to hHexB.

### Preparation of 2′-alkylaminothiazoline OGA inhibitors for evaluating electronic effects in OGA inhibition

Notably, ThiamEt-G (*K*_i_ = 2.1 nM) binds over 100-fold more tightly than NButGT (*K*_i_ = 230 nM) and is 25-fold more selective for hOGA. Detailed mechanistic studies in combination with pH-rate profiles of wild-type and mutant hOGA revealed the key catalytic residue Asp^174^ in the OGA catalytic site (Asp^242^ in *Bt*GH84) acts as a general base to assist the attack of the substrate 2-acetamido group onto the anomeric center. The kinetic p*K*_a_ of this residue was determined to be 5.2 so that at physiological pH this residue is expected to be in its carboxylate form^33^ and therefore suitably ionized in the resting enzyme to facilitate catalysis. Given that aminothiazolines are known to be more basic than thiazolines, it was speculated that installation of the 2′-alkylamino group would increase the basicity of ThiamEt-G as compared to NButGT and thereby contribute to its enhanced potency relative to inhibitors bearing 2′-alkyl groups at physiological pH. Structural data of ThiamEt-G bound within the active site of *Bt*GH84 is consistent with this proposal, revealing that both the endo and exocyclic amines engaged Asp242 of *Bt*GH84.^19^

We set out to evaluate this proposal and assess the importance of the inhibitor p*K*_a_ on potency by studying a series of 2′-aminoethylthiazoline inhibitors with increasing fluorine substitution at the terminal methyl group (**15f–h**). Evaluation of the potency of these compounds revealed a progressive increase in *K*_i_ value upon increasing substitution with fluorine [*K*_i_ = 2.1 (CH_3_, **15c**) 15 (CH_2_F_,_**15f**), 60 (CHF_2_, **15g**) and 1000 (CF_3_, **15h**) nM] (Table 1). To clearly understand the relationship between inhibitor basicity and potency we used ^13^C NMR titration to determine the p*K*_a_ values of conjugate acids of these inhibitors (**15c**, **15f–h**). NMR methods are highly accurate and can be used to determine relative p*K*_a_ values between one or more compounds having an unknown p*K*_a_ and a reference compound having a well established p*K*_a_ value.^49^

Practically, two advantages of this relative measurement approach is that it does not require repeated pH measurements throughout the titration and it can be used to evaluate small changes in p*K*_a_ values. In this way, a non-linear plot of the difference between NMR resonance frequencies for a compound of interest and those for a standard, such as 3-nitrophenol, can be used to determine the ratio (*R*) of the acid dissociation constants between these two materials. A typical plot for the difference in ^13^C chemical shifts is shown in Fig. 4 (see ESI† methods for a full discussion).

To assess the extent to which inhibitor potency depends on its p*K*_a_ value, we plotted the p*K*_a_ value of inhibitors (**15c**, **15f–h** and NButGT, **4**) with the corresponding log *K*_i_, which are both free energy terms. The resulting linear free energy relationship (LFER) shows a linear correlation (*R*^2^ = 0.9876) with a slope of –1.12 ± 0.09 (Fig. 5), which is consistent with the p*K*_a_ value of inhibitors mostly dominating the effect of binding as compared to steric effects associated with increasing fluorine substitution. Notably, we also find that NButGT, which is isosteric to ThiamEt-G (**15c**), matches reasonably well within this correlation, supporting the electronic effects dominating this correlation. While the affinities of each protonation state of these inhibitors for hOGA (Fig. 5) cannot be readily determined because the enzyme itself has various ionization states, these data collectively suggest that the p*K*_a_ value of the inhibitor, either by favoring the protonated inhibitor form or by optimizing hydrogen bonding strength, plays a key role in binding of these 2′-aminothiazoline inhibitors.

### Assessment of 2′-aminothiazoline inhibitors as transition state analogs

The tight binding of these 2′-aminothiazoline inhibitors prompted us to consider their potency in the context of their size. One widely used parameter to understand the efficiency of binding as a function of molecular weight is to consider the ligand efficiency (LE) of a ligand. This measure provides the binding affinity of the compound as a measure of the number of heavy atoms. We calculate a remarkably high LE of 0.88 kcal per mol per heavy atom for compound **15a**. Such a LE is comparable to some of the highest ever observed LEs observed for compounds in the size range of between 10–50 heavy atoms^50^ and suggests to us that these compounds could well be TS analogues, as had been observed for the related thiazoline inhibitors^51^. Tight-binding inhibitors that bear resemblance to enzyme substrates or intermediates have often been considered to be TS analogues simply by virtue of their potency. However, because enzymes are thought to catalyze reactions by tightly binding the TS, for genuine TS analogues changes in free energies of binding of a series of TS analogues (log *K*_i_) should parallel changes in the free energies of a series of related transition states TS (log *k*_cat_/*K*_m_). Bartlett has formalized these concepts and methods to quantitatively assess whether compounds are TS analogues using LFERs.^52^,^53^ Using this method, genuine TS analogues yield plots of log *K*_m_/*k*_cat_ values, for a series of substrates having defined structural differences, *versus* log *K*_i_ values, for a series of inhibitors having the analogous structural changes, which show linear correlations having a slope of unity. Furthermore, log *K*_m_*versus* log *K*_i_ are not correlated for TS analogues but do correlate for substrate analogues.^52^

**Scheme 2:**
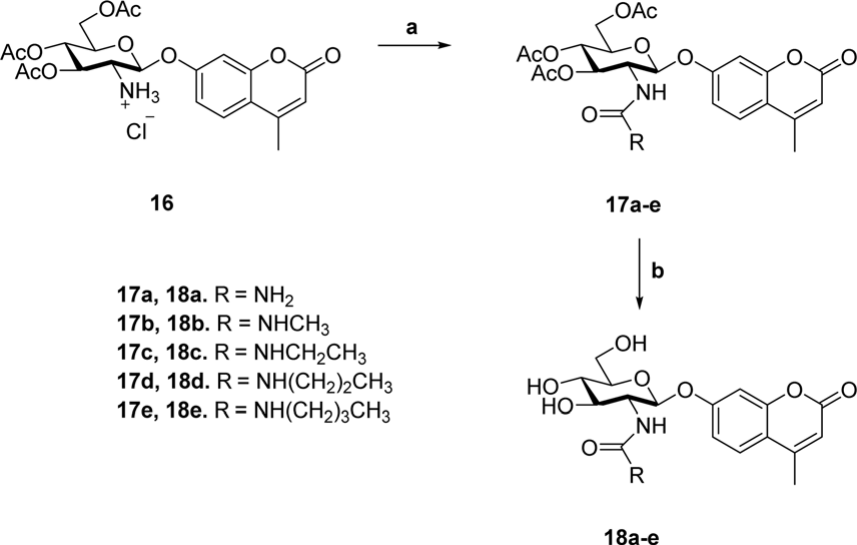
Synthesis of 4-methylumbelliferyl fluorogenic substrates **18a–e**. (a) R-NCO, NEt_3_, CH_3_CN; (b) (i) NaOMe, MeOH; (ii) Dowex 50-H^+^.

Previous studies showed that NAG-thiazoline analogues are TS analogues despite their obvious resemblance to the oxazoline intermediate, perhaps due to the longer C–S bonds altering the thiazoline ring to resemble a late TS.^51^ Given the greater than 100-fold increase in potency we observe for the 2′-aminothiazoline inhibitors over their thiazoline counterparts and their structural resemblance to the oxazoline intermediate found along the reaction coordinate of hOGA, we wanted to assess whether incorporation of the 2′-amino group benefitted binding through serendipitous interactions, or whether the presence of the charge included in this class of inhibitors also made them TS analogues. We therefore turned to using the Bartlett LFER approach. With the series of inhibitors in hand we synthesized a series of fluorogenic 4-methylumbelliferyl 2-deoxy-2-urea-β-d-glucopyranoside substrates (**18a–e**, Scheme 2) bearing *N*-alkyl substituents on the terminal urea nitrogen that correspond to those alkyl groups present on the series of 2′-aminothiazoline inhibitors (**11a**, **15a**, **15c–e**). We started from 4-methylumbelliferyl 2-amino-2-deoxy-β-d-glucopyranoside hydrochloride (**16**) as a common intermediate.^54^ Per-*O*-acetylated urea substrates **17a–e** were prepared by reacting **16** with the appropriate alkylisocyanate in the presence of triethylamine, followed by Zemplen de-*O*-acetylation. With this series of substrates (**18a–e**) in hand we determined the *k*_cat_/*K*_m_ values governing their hOGA catalyzed hydrolysis (ESI Table S1†). Plotting these data to assess TS analogy, we observe (Fig. 6) a clear correlation (*R*^2^ = 0.9950) with a slope of 1.08 ± 0.04 between log *K*_i_ values for the inhibitors *versus* the log *K*_m_/*k*_cat_ values for the corresponding series of urea substrates (Fig. 6A). In contrast, we find no correlation between log *K*_i_ values for the inhibitors and log *K*_m_ values for the series of substrates (Fig. 6B). These results indicate the 2′-aminothiazoline inhibitors are TS analogues for the hOGA catalyzed hydrolysis of urea substrates.

We recognized that our observations showed 2′-aminothiazoline inhibitors are TS analogues for the hOGA-catalyzed hydrolysis of unnatural urea substrates (**18a–e**), however, we were curious as to whether these inhibitors would also be TS analogues for the hOGA-catalyzed hydrolysis of the more natural *N*-acyl substrates. To address this question, we examined the correlation between log *K*_i_ values for the same series of 2′-aminothiazoline inhibitors and the series of *N*-acyl substrates (Fig. 6C) having the analogous structural changes for which *k*_cat_/*K*_m_ values are reported (Fig. 6D).^51^ We find a fair correlation (*R*^2^ = 0.9768) with a slope of 2.3 ± 0.3. For this analysis we excluded the 2-aminothiazoline (**11a**) because of its unexpectedly poor inhibition of hOGA (Table 1).

Notably, previous studies have shown *k*_cat_ and *k*_cat_/*K*_m_ values for the OGA-catalyzed hydrolysis of aryl 2-acetamido-2-deoxy-glucopyranosides, including **19a**, vary in according to the p*K*_a_ value of the phenolic leaving group.^55^ Additionally, such substrates bearing different sized *N*-acyl groups, including **19a–d** (ref. 51) coupled with the large ^α–D^(V)-KIE (*k*_H_/*k*_D_ = 1.14 ± 0.02) value observed for the OGA-catalyzed hydrolysis of *p*-nitrophenol 2-acetamido-2-deoxy-glucopyranoside^55^ all support *k*_cat_ and *k*_cat_/*K*_m_ values reflect a chemical step being rate limiting for human OGA, which supports the validity of this TS analogy study.

The steep slope observed for this series of 2-acyl substrates in correlation with the aminothiazoline inhibitors is surprising. However, slopes other than unity in Bartlett LFER plots are precedented though not often rationalized.^56^ Here, we interpret this steep slope as indicating that the TS for the hOGA catalyzed hydrolysis of *N*-acyl substrates bears less positive charge in the forming oxazoline ring system. This may arise because the amide proton is in flight in the TS, as compared to the *N*-urea substrates (Fig. 6E and F), which are expected to be more basic and therefore likely lead to the formation of 2′-amino-oxazolinium ion intermediates that retain their proton. Accordingly, the TS leading to such aminooxazolinium ion intermediates is expected to have more positive charge than the corresponding transition state leading to the oxazoline intermediate (Fig. 6E and F). In keeping with this proposal, it is notable that site-directed deletion of the side chain of Asp^174^, which is the catalytic general acid/base catalytic residue that interacts with the acetamido group of the substrate, leads to a similar drop of between 150 to 750-fold in second order rate constant^33^ as seen on going from *N*-acyl to *N*-urea substrates (250-fold). Accordingly, these data indicate that 2′-aminothiazoline inhibitors are TS analogues for hOGA, by virtue of both their shape and general charge distribution. However, the steep slope observed for the LFER between log *K*_m_/*k*_cat_ values observed for *N*-acyl substrates and the log *K*_i_ values seen for the 2′-aminothiazoline inhibitors suggests that this feature lends improved binding over the corresponding partial charge that likely develops for the TS found for the hOGA catalyzed processing of natural *N*-acyl-containing substrates. These data support a catalytic mechanism in which residue D174 of hOGA acts as a general acid/base catalytic residue rather than simply stabilizing an oxazolinium ion intermediate as proposed for GH20 β-hexosaminidases.^57^

## Conclusion

In summary, we describe a series of aminothiazoline inhibitors for human OGA having picomolar and low nanomolar *K*_i_ values. The great potency of this inhibitor family is in large part attributable to their p*K*_a_ values since a clear correlation was observed between the p*K*_a_ and log *K*_i_ of a series of these compounds. Structures of these inhibitors in complex with BtOGA reveal the molecular basis for the trends in observed inhibitor potencies and selectivities. Using quantitative methods we find that these 2′-aminothiazoline inhibitors are tight-binding TS analogues for hOGA. These inhibitors benefit from their formal positive charge at physiological pH, harnessing favorable interactions that are only partly realized within the transition state for the natural 2-acyl-containing substrates. These observations should permit the design of more potent and selective inhibitors, not only in this class of inhibitor but also using other inhibitor scaffolds. Finally, the great potencies and selectivities of these inhibitors reveal a series of useful tool compounds that can be used to manipulate hOGA activity *in vivo*.

## Supplementary Material

Supplementary information
