# Supplementary material for: Analysis of transition state mimicry by tight binding aminothiazoline inhibitors provides insight into catalysis by human O-GlcNAcase
Source: Chem Sci. 2016 Feb 15;7(6):3742–50. doi: 10.1039/c6sc00370b (PMC6008586; doi:10.1039/c6sc00370b)
Supplement: Supplementary file 1 [file SC-007-C6SC00370B-s001.pdf]

Electronic Supplementary Material (ESI) for Chemical Science.
This journal is © The Royal Society of Chemistry 2016

Electronic Supplementary Information

Analysis of transition state mimicry by tight binding aminothiazoline inhibitors provides insight into catalysis by human *O*-GlcNAcase.

*N. Cekic,[a,†] J. E. Heinonen,[a,†] K. A. Stubbs,[a,b] C. Roth,[c] Y. He,[c] A. J. Bennet,[a] E. J. McEachern,[a,‡] G. J. Davies,[c] D. J. Vocadlo[a,d,]**

[a] Department of Chemistry, Simon Fraser University, Burnaby, British Columbia, V5A 1S6, Canada

[b] School of Chemistry and Biochemistry, The University of Western Australia (M313), 35 Stirling Highway, Crawley WA 6009, Australia

[c] York Structural Biology Laboratory, Department of Chemistry, The University of York, YO10 5DD, U.K.,

[d] Department of Molecular Biology and Biochemistry, Simon Fraser University, Burnaby, British Columbia, V5A 1S6, Canada

*Correspondence: dvocadlo@sfu.ca

Experimental Section:

General Information. All dry solvents and buffer salts were used as purchased from Sigma-Aldrich and all reagents were utilized without further purification. The progress of all reactions was monitored on Merck pre-coated silica gel plates using combinations of ethyl acetate/n-hexane (EtOAc/Hexanes), ethyl acetate/dichloromethane (EtOAc/DCM), or methanol/dichloromethane (MeOH/DCM) solvent elution systems. Flash chromatography was performed under a positive pressure with Fisher Scientific silica gel (230-400 mesh) where spots were visualized by irradiation with ultraviolet light (254 nm), staining with $KMnO_4$ and charring with 10% ammonium molybdate in 2M H_2SO_4 upon heating. Proton (^{1}H) and carbon (^{13}C) NMR spectra were established on either a Bruker Avance 500 (500 MHz for ^{1}H, 125 MHz for ^{13}C, Bruker Avance 400 (400 MHz for ^{1}H, 101 MHz for ^{13}C), or Bruker Avance II 600 (600 MHz for ^{1}H, 151 MHz for ^{13}C) using $CDCl_3$, DMSO-d_6, CD_3OD, or D_2O as the sample solvent. Chemical shifts are given in parts per million (ppm) (δ relative to a residual solvent peak for ^{1}H and ^{13}C). High resolution mass spectrometry (HRMS) analysis was performed using a Bruker maXis TOF LC/MS/MS instrument.

Synthetic procedures. The per-acetylated glucosamine salt **8** was made using a known literature procedure by Gonzales *et al*[1] which described its conversion to the carbohydrate isothiocyanate intermediate **12** and its subsequent transformation to a series of carbohydrate amino thiazoline analogues.

1,3,4,6-*tetra-O*-acetyl-2-amino-2-deoxy-β-D-glucopyranose hydrochloride (8). ^{1}H-NMR (400 MHz, CD_3OD) δ (ppm) 5.87 (d, J = 8.8Hz, 1H), 5.37 (dd, J = 10.5, 9.2 Hz, 1H), 5.11 (dd, J = 10.0, 9.2 Hz, 1H), 4.33 (dd, J = 12.6, 4.6 Hz, 1H), 4.14 (dd, J = 12.6, 2.3 Hz, 1H), 4.04 (ddd, J = 10.1, 4.6, 2.3 Hz, 1H), 3.62 (dd, J = 10.5, 8.8 Hz, 1H), 2.22 (s, 3H), 2.12 (s, 3H), 2.06 (s, 3H), 2.05 (s, 3H); ^{13}C-NMR (125 MHz, H_2O+D_2O) δ (ppm) 173.53, 172.97, 172.64, 171.17, 90.41, 72.12, 70.87, 67.99, 61.42, 52.44, 20.21, 20.19, 20.08, 20.03; HRMS (m/z): [M+H]+ calculated for $C_{14}H_{22}NO_9$: 348.1295; found 348.1250; [M+Na]+ calculated for $C_{14}H_{21}NNaO_9$: 370.1114; found 370.1114; [M+K]+ calculated for $C_{14}H_{21}NKO_9$: 386.0853; found 386.0849.

Preparation of isothiocyanate intermediate 12. Synthesis was performed with minor modifications to the procedure described by Gonzalez *et al.*[1] After combining amine **8** (10.0g, 26.1 mmol), and $CaCO_3$ (7.9 g, 78.3 mmol) in a round bottom flask at room temperature, DCM and H_2O (17 mL each) were added. Thiophosgene (3.0 mL, 39.2 mmol) was then added drop-wise, followed by further addition of DCM and H_2O (13 mL each). The reaction was monitored by TLC and stirred at room temperature (~ 3 hours). The aqueous layer of the mixture was extracted twice with DCM. The organic layers were collected, dried over $MgSO_4$, filtered and concentrated to afford the crude compound **12** as a sticky dark yellow solid (8.73g, 86%). The product was used with no further purification necessary.

1,3,4,6-*tetra-O*-acetyl-2-deoxy-2-isothiocyanato-β-D-glucopyranose (12). ^{1}H-NMR (400 MHz, $CDCl_3$) δ (ppm) 5.69 (d, J = 8.6 Hz, 1H), 5.29 – 5.18 (t, 1H), 5.04 – 4.94 (t, 1H), 4.27 (dd, J = 12.4, 4.3 Hz, 1H), 4.13 – 4.03 (m, 1H), 3.96 (dd, J = 10.3, 8.6 Hz, 1H), 3.88 – 3.81 (m, 1H), 2.17 (s, 3H), 2.08 (s, 3H), 2.05 (s, 3H), 2.01 (s, 3H); ^{13}C-NMR (100 MHz, $CDCl_3$) δ (ppm) 170.45, 169.58, 169.48, 168.49, 142.07, 91.72, 72.93, 72.54, 67.46, 61.29, 59.40, 20.83, 20.65, 20.62, 20.50; HRMS (m/z): [M+Na]+ calculated for $C_{15}H_{19}NNaO_9S$: 412.0678; found 412.0677; [M+K]+ calculated for $C_{15}H_{19}KNO_9S$: 428.0418; found 428.0414.

Synthesis of thiourea 9a from amine 8. To a stirred solution of **8** (250 mg, 0.65 mmol) in DCM was added triethylamine (0.9 mL, 0.65 mmol). The solution was diluted with 20 mL of saturated $NaHCO_3$, and the resulting mixture extracted with DCM (3 x 10 mL). The combined organic layers were dried with Na_2SO_4 and concentrated to give 220 mg of 1,3,4,6-*tetra-O*-acetyl-2-amino-2-deoxy-β-D-glucopyranose which was used without purification. 1,3,4,6-*tetra-O*-acetyl-2-amino-2-deoxy-β-D-glucopyranose (220 mg) was then dissolved in 5 mL of pyridine and 9-fluorenylmethoxycarbonyl isothiocyanate (Fmoc) (180 mg, 0.65 mmol) and triethyl amine (0.02 mL) were added. The resulting mixture was stirred at room temperature for 16 hours. The solution was concentrated and the residue diluted with DCM (20 mL) and $NaHCO_3$ (20 mL), and subsequently extracted with DCM (3 x 10 mL). The combined organic extracts were dried with Na_2SO_4 and concentrated. Flash chromatography was performed with 75% EtOAc:hexanes to yield **9a** as a white foam (360 mg, 89% yield over two steps).

1,3,4,6-*tetra-O*-acetyl-2-deoxy-2-[[[(2-fluoren-9-yl methoxycarbonyl)amino]thioxomethyl]amino]-β-D-glucopyranose (9a). ^{1}H NMR (500 MHz, $CDCl_3$) δ 7.79 (d, *J* = 7.7 Hz, 2H), 7.56 (dq, *J* = 7.6, 0.8 Hz, 2H), 7.44 (td, *J* = 7.5, 1.0 Hz, 2H), 7.34 (dd, *J* = 7.5, 1.1 Hz, 2H), 5.84 (d, *J* = 8.2 Hz, 1H), 5.24 (dt, *J* = 38.6, 9.1 Hz, 2H), 5.07 (q, *J* = 9.3 Hz, 1H), 4.54 (d, *J* = 6.6 Hz, 2H), 4.31 (ddd, *J* = 12.2, 7.5, 4.6 Hz, 1H), 4.24 (t, *J* = 6.6 Hz, 1H), 4.16 (dd, *J* = 12.4, 2.6 Hz, 1H), 3.86 (ddd, *J* = 9.4, 4.7, 2.7 Hz, 1H), 2.13 (d, *J* = 0.5 Hz, 3H), 2.11 (d, *J* = 0.5 Hz, 3H), 2.06 (d, *J* = 0.5 Hz, 3H), 2.05 (d, *J* = 0.5 Hz, 3H). ^{13}C NMR (150 MHz, $CDCl_3$) δ 180.22, 170.72, 170.46, 169.34, 169.24, 152.16, 142.80, 141.37, 129.06, 128.25, 128.15, 127.83, 127.30, 125.32, 124.96, 124.84, 120.29, 120.09, 92.21, 72.87, 72.21, 68.40, 67.47, 61.65, 57.59, 46.49, 21.05, 20.77, 20.72, 20.62.

Synthesis of thiourea 9b from amine 8. 1 g (2.6 mmol) of **8** was dissolved in CH_3CN. Triethylamine (0.7 mL, 5.2 mmol) was added, followed by allyl isothiocyanate (0.5 mL, 5.2 mmol). The reaction mixture was stirred at room temperature for ~2 hours, followed by work-up with a minimal amount of saturated $NaHCO_3$ after completion. The aqueous layer formed was extracted twice with DCM and the organic layers were then combined, dried over $MgSO_4$, filtered and concentrated. The crude product was purified by silica flash chromatography (1:1 EtOAc/Hexanes), affording a solid, white product (70%, 785 mg).

1,3,4,6-*tetra-O*-acetyl-2-deoxy-2-[[[(2-prop-1-ene)amino]thioxomethyl]amino]-β-D-glucopyranose (9b). ^{1}H NMR (500 MHz, $CDCl_3$) δ 6.34 (s, 1H), 6.21 (s, 1H), 5.91 – 5.82 (m, 1H), 5.75 (dd, J = 8.5, 1.3 Hz, 1H), 5.32 – 5.14 (m, 5H), 4.29 (ddd, J = 12.5, 4.7, 1.9 Hz, 1H), 4.19 – 4.07 (m, 2H), 3.87 (ddd, J = 9.7, 4.7, 2.3 Hz, 1H), 2.14 (s, 3H), 2.11 (s, 3H), 2.09 (s, 3H), 2.06 (s, 3H), 1.98 – 1.87 (m, 1H); ^{13}C-NMR (100 MHz, $CDCl_3$) δ (ppm) 183.51, 171.5, 171.2, 170.68, 169.27, 136.36, 117.43, 92.95, 72.93, 72.90, 67.64, 61.62, 57.77, 53.74, 21.04, 20.82, 20.74, 20.59; HRMS (m/z): [M + H]+ calculated for $C_{18}H_{27}N_2O_9S$: 447.1437 (M+H); found 447.1430; [M + Na]+ calculated for $C_{18}H_{26}N_2NaO_9S$: 469.1251; found 469.1251; [M+K]+ calculated for $C_{18}H_{26}KN_2O_9S$: 485.0996; found 485.1005.

General synthesis of thioureas 13a-c, 13f-h. Isothiocyanate **12** (1 eq) was dissolved in CH_3CN followed by addition of the desired amine hydrochloride salt (1.2-2.0 eq), then drop-wise addition of triethylamine (2.0 eq). The reaction mixture was stirred for ~4 hours by monitoring by TLC. Upon completion, it was washed with a minimal amount of saturated $NaHCO_3$. The aqueous layer was extracted twice with DCM and the organic layers were combined, dried over $MgSO_4$, filtered and concentrated. The crude product was purified by silica flash chromatography (1:1 EtOAc/hexanes or 75% EtOAc in hexanes) to afford the pure product.

General synthesis of thioureas 13d-e. Isothiocyanate **12** (1 eq) was dissolved in DCM, followed by drop-wise addition of the desired amine (1.05 eq). The reaction mixture was stirred for ~4 hours by monitoring by TLC. Upon completion, it was washed with a minimal amount of saturated $NaHCO_3$. The aqueous layer was extracted twice with DCM and the organic layers were combined, dried over $MgSO_4$, filtered and concentrated. The crude product was purified by silica flash chromatography (1:1 EtOAc/hexanes or 75% EtOAc in hexanes) to afford the pure product. Products were isolated in yields ranging from 60 to 87%.

1,3,4,6-*tetra-O*-acetyl-2-deoxy-2-[(aminomethyl)thioxomethyl]amino]-β-D-glucopyranose (13a): ^{1}H NMR (400 MHz, $CDCl_3$) δ 6.56 (s, 1H), 6.41 (s, 1H), 5.78 (d, J = 8.4 Hz, 1H), 5.27 (t, J = 9.6 Hz, 1H), 5.13 (t, J = 9.6 Hz, 1H), 4.24 (dd, J = 12.5, 4.6 Hz, 1H), 4.10 (dt, J = 14.3, 4.6 Hz, 2H), 3.85 (ddd, J = 9.9, 4.5, 2.3 Hz, 1H), 2.93 (s, 3H), 2.09 (s, 3H), 2.06 (s, 3H), 2.03 (s, 3H), 2.02 (s, 3H); ^{13}C NMR (101 MHz, $CDCl_3$) δ 171.52, 170.75, 169.55, 169.36, 92.85, 73.14, 72.73, 67.90, 61.76, 57.63, 20.98, 20.78, 20.69, 20.55. HRMS (m/z): [M+H] + calculated for $C_{16}H_{25}N_2O_9S$: 421.1281 found 421.1227; [M+Na]+ calculated for $C_{16}H_{24}N_2NaO_9S$: 443.1134; found 443.1098; [M+K]+ calculated for $C_{16}H_{24}KN_2O_9S$: 459.0840; found 459.0840.

1,3,4,6-*tetra-O*-acetyl-2-deoxy-2[(dimethylamino)thioxomethyl]amino]-β-D-glucopyranose (13b): ^{1}H NMR (500 MHz, $CDCl_3$) δ 5.74 (t, J = 9.7 Hz, 1H), 5.70 (d, J = 9.2 Hz, 1H), 5.36 – 5.23 (m, 1H), 5.23 – 5.13 (m, 2H), 4.21 (dt, J = 15.7, 7.9 Hz, 1H), 4.12 – 4.09 (m, 1H), 3.80 (ddd, J = 9.1, 4.5, 2.2 Hz, 1H), 3.17 (s, 6H), 2.07 (s, 3H), 2.06 (s, 3H), 2.01 (d, J = 3.4 Hz, 3H), 2.00 (s, 3H). ^{13}C-NMR (125 MHz, $CDCl_3$) δ (ppm) 182.29, 171.83, 170.68, 169.94, 169.06, 93.28, 73.24, 72.99, 67.52, 61.71, 58.46, 40.63, 21.06, 20.78, 20.69, 20.53; HRMS (m/z): [M + H]+ calculated for $C_{17}H_{26}N_2O_9S$: 489.1149; found 489.1152; [M + Na]+ calculated for $C_{17}H_{26}N_2O_9S$: 511.0969; found 511.0970.

1,3,4,6-*tetra-O*-acetyl-2-deoxy-2-[[[(2-propyl)amino]thioxomethyl]amino]-β-D-glucopyranose (13d): ^{1}H NMR (400 MHz, $CDCl_3$) δ 6.22 (s, 1H), 5.98 (d, J = 26.1 Hz, 1H), 5.76 (d, J = 8.5 Hz, 1H), 5.23 – 5.20 (m, 2H), 4.30 (dd, J = 12.5, 4.6 Hz, 1H), 4.27 – 4.21 (m, 1H), 4.20 – 4.10 (m, 1H), 3.87 (ddt, J = 6.8, 4.5, 2.3 Hz, 1H), 3.46 – 3.28 (m, 2H), 2.16 (s, 3H), 2.12 (s, 3H), 2.09 (s, 3H), 2.07 (s, 3H), 1.62 (qd, J = 7.3, 2.0 Hz, 2H), 0.98 (t, 3H). ^{13}C NMR (101 MHz, $CDCl_3$) δ 171.09, 170.95, 170.73, 169.27, 99.98, 93.09, 72.99, 67.60, 62.10, 61.68, 21.04, 20.93, 20.81, 20.75, 20.65, 20.59, 11.30. HRMS (m/z): [M + H]+ calculated for $C_{18}H_{29}N_2O_9S$: 449.1594; found 449.1593.

1,3,4,6-*tetra-O*-acetyl-2-deoxy-2-[[[(2-butyl)amino]thioxomethyl]amino]-β-D-glucopyranose (13e): ^{1}H NMR (400 MHz, $CDCl_3$) δ 6.38 (s, 1H), 5.76 (d, *J* = 8.5 Hz, 1H), 5.36 – 5.07 (m, 2H), 4.30 – 4.16 (m, 2H), 4.15 – 4.09 (m, 1H), 3.85 (ddd, *J* = 9.7, 4.6, 2.3 Hz, 1H), 3.58 – 3.13 (bs, 1H), 2.11 (s, 3H), 2.08 (s, 3H), 2.03 (s, 3H), 2.02 (s, 3H), 1.56 – 1.48 (m, 2H), 1.38 – 1.30 (m, 2H), 0.91 (t, *J* = 7.3 Hz, 3H). ^{13}C-NMR (100 MHz, $CDCl_3$) δ (ppm) 171.60, 171.00, 169.39, 169.27, 93.07, 72.97, 68.10, 67.62, 62.10, 61.69, 30.90, 21.15, 20.94, 20.86, 20.71, 20.12, 20.09, 13.80; HRMS (m/z): [M + H] + calculated for $C_{19}H_{31}N_2O_9S$: 463.1750; found 463.1737.

1,3,4,6-*tetra-O*-acetyl-2-deoxy-2-[[[(2-fluoroethyl)amino]thioxomethyl]amino-β-D-glucopyranose (13f): ^{1}H-NMR (500 MHz, $CDCl_3$) δ (ppm) 6.43 (s, 1H), 5.99 (d, J = 7.6 Hz, 1H), 5.64 (d, J = 8.6 Hz, 1H), 5.20 – 5.11 (m, 2H), 4.70 – 4.50 (m, 2H), 4.31 (dd, J = 12.5, 4.5 Hz, 1H), 4.14 – 4.13 (m, 1H), 3.82 (ddd, J = 9.4, 4.5, 2.3 Hz, 2H), 2.14 (s, 3H), 2.10 (s, 3H), 2.09 (s, 3H), 2.04 (s, 3H); ^{13}C-NMR (125 MHz, $CDCl_3$) δ (ppm): 184.30, 171.94, 171.62, 171.05, 169.76, 93.10,

82.77 (d, J = 166.3 Hz), 73.30, 72.91, 68.18, 61.93, 57.79, 45.57, 21.17, 21.02, 20.99, 20.84; HRMS (m/z): [M+H]+ calculated for $C_{17}H_{26}FN_2O_9S$: 453.1343; found 453.1342.

1,3,4,6-*tetra-O*-acetyl-2-deoxy-2-[[[(2,2-difluoroethyl)amino]thioxomethyl]amino-β-D-glucopyranose (13g): ^{1}H-NMR (400 MHz, $CDCl_3$) δ 6.55 (s, 2H), 5.99 (t, J = 56.1 Hz, 1H), 5.74 (d, J = 8.6 Hz, 1H), 5.32 – 5.21 (m, 1H), 5.11 (t, J = 9.6 Hz, 1H), 4.27 (dd, J = 12.5, 4.4 Hz, 1H), 4.14 (d, J = 6.5 Hz, 1H), 4.09 (td, J = 7.2, 1.2 Hz, 2H), 3.88 (ddd, J = 9.8, 4.2, 2.0 Hz, 3H), 2.11 (s, 3H), 2.08 (s, 3H), 2.06 (s, 3H), 2.04 (s, 3H); ^{13}C-NMR (101 MHz, $CDCl_3$) δ (ppm) 184.72, 171.57, 171.54, 170.91, 169.61, 113.54 (t, J =241.5 Hz, 1C), 92.65, 72.95, 72.57, 68.05, 61.78, 57.51, 46.42 (t, J=27.2 Hz) 20.77, 20.62, 20.60, 20.48; HRMS (m/z): [M+H]+ calculated for $C_{17}H_{25}F_2N_2O_9S$: 471.1249; found 471.1242; [M+Na]+ calculated for $C_{17}H_{25}F_2NaN_2O_9S$: 493.1068; found 493.1065.

1,3,4,6-*tetra-O*-acetyl-2-deoxy-2-[[[(2,2,2 trifluoroethyl)amino]thioxomethyl]amino-β-D-glucopyranose (13h): ^{1}H-NMR (400 MHz, $CDCl_3$) δ (ppm) 6.62 – 6.49 (m, 2H), 5.79 (d, J = 8.5 Hz, 1H), 5.30 (t, J = 9.8 Hz, 1H), 5.16 (t, J = 9.6 Hz, 1H), 4.55 – 4.40 (m, 1H), 4.31 (dd, J = 12.4, 4.7 Hz, 2H), 4.22 – 4.11 (m, 2H), 3.94 (ddd, J = 9.8, 4.5, 2.2 Hz, 1H), 2.14 (s, 3H), 2.13 (s, 3H), 2.10 (s, 3H), 2.08 (s, 3H); ^{13}C-NMR (125 MHz, $CDCl_3$) δ (ppm) 184.83, 171.76, 171.63, 170.88, 169.62, 124.01 (q, J = 279.2 Hz), 92.44, 72.77, 72.42, 68.04, 61.66, 57.52, 45.23, 20.59, 20.52, 20.46, 20.36; HRMS (m/z): [M+H]+ calculated for $C_{17}H_{23}F_3N_2O_9S$: 489.1155; found 489.1153; [M+Na]+ calculated for $C_{17}H_{23}F_3NaN_2O_9S$: 511.0974; found 511.0970.

Synthesis of per-acetylated thiazoline 10a. Thiourea **9a** (200 mg, 0.32 mmol) was dissolved in 4 mL of DCM and $SnCl_4$ (0.5 mL, 4.0 mmol) was then added. The resulting mixture was stirred at room temperature for 16 hours. The solution was diluted with 20 mL of saturated $NaHCO_3$ and the mixture was then extracted with DCM (3 x 10 mL). The combined organic extracts were dried with Na_2SO_4 and concentrated. Silica flash chromatography was used to purify the crude mixture with 75% EtOAc/hexanes to yield **10a** as a white foam (125 mg, 65% yield).

3,4,6-*tri-O*-acetyl-1,2-dideoxy-2'[(-[[[(2-fluoren-9-yl methoxycarbonyl)amino]thioxomethyl]amino]-α-D-glucopyranoso[2,1-d]-Δ2'-thiazoline (10a): ^{1}H NMR (500 MHz, $CDCl_3$) δ 7.80 (dd, *J* = 7.6, 2.7 Hz, 2H), 7.59 (dd, *J* = 7.6, 4.3 Hz, 2H), 7.45 – 7.40 (m, 2H), 7.34 (dtd, *J* = 15.0, 7.5, 1.1 Hz, 2H), 6.01 (d, *J* = 6.9 Hz, 1H), 5.24 (t, *J* = 4.7 Hz, 1H), 4.95 (dd, *J* = 9.6, 4.2 Hz, 1H), 4.74 (s, 1H), 4.55 (dd, *J* = 10.8, 6.0 Hz, 1H), 4.29 – 4.21 (m, 2H), 4.14 – 4.12 (m, 1H), 3.81 (s, 1H), 3.70 (s, 1H), 2.12 (s, 3H), 2.04 (s, 3H), 1.89 (s, 3H). ^{13}C NMR (125 MHz, $CDCl_3$) δ 20.62, 20.71, 21.35, 46.78, 57.91, 63.70, 67.92, 69.46, 71.88, 72.52, 90.23, 120.71, 120.89, 123.83, 124.04, 125.15, 127.76, 127.97, 129.61, 129.72, 130.08, 141.31, 142.86, 152.11, 161.43, 169.11, 169.63, 170.23.

General synthesis of per-acetylated thiazolines 10b and 14a-h. Thiourea derivatives **9b, 13a-13h** (1 eq) were each dissolved in DCM under argon, after which trifluoroacetic acid (7.5 eq) was added drop-wise. The reaction was stirred under an atmosphere of argon overnight (~10 hours) until judged complete by TLC analysis. The mixture was washed twice with saturated $NaHCO_3$, followed by extraction of the aqueous layers 3 times with DCM. The organic layers were combined, dried over $MgSO_4$, filtered and concentrated to obtain the crude product which was then purified using silica flash chromatography (generally 1:1 EtOAc/hexanes or 75% EtOAc/hexanes). Products were isolated in yields ranging from 55 to 84%.

3,4,6-*tri-O*-acetyl-1,2-dideoxy-2'[(2-prop-1-ene)amino]-α-D-glucopyranoso[2,1-d]-Δ2'-thiazoline (10b): ^{1}H NMR (400 MHz, $CDCl_3$) δ 6.21 (d, J = 6.5 Hz, 1H), 5.90 (ddt, J = 16.9, 10.6, 5.5 Hz, 1H), 5.38 (dd, J = 3.9, 2.8 Hz, 1H), 5.17 (ddd, J = 13.7, 11.5, 1.3 Hz, 2H), 4.94 – 4.89 (m, 1H), 4.36 – 4.31 (m, 1H), 4.13 – 4.07 (m, 3H), 3.97 – 3.89 (m, 1H), 3.83 (ddd, J = 11.4, 9.2, 5.0 Hz, 2H), 2.09 (s, 3H), 2.06 (s, 3H), 2.05 (s, 3H).; ^{13}C-NMR (125 MHz, $CDCl_3$) 170.36, 169.38, 169.18, 159.98, 133.93, 116.06, 89.30, 72.25, 71.40, 68.88, 68.12, 62.97, 46.51, 20.71, 20.58, 20.49. HRMS (m/z): [M + H]+ calculated for $C_{16}H_{23}N_2O_7S$: 387.1226 found 387.1221; [M+Na]+ calculated for $C_{16}H_{22}NaN_2O_7S$: 409.1045; found 409.1038.

3,4,6,-*tri-O*-acetyl-1,2-dideoxy-2'-aminomethyl-α-D-glucopyranoso[2,1-d]-Δ2'-thiazoline (14a): ^{1}H-NMR (400 MHz, $CDCl_3$) δ (ppm) 6.25 (d, J = 6.5 Hz, 1H), 5.43 (dd, J = 4.1, 3.0 Hz, 1H), 4.95 (ddd, J = 9.6, 2.8, 0.9 Hz, 1H), 4.37 (dd, J = 6.0, 4.5 Hz, 1H), 4.17-4.14 (m, 2H), 3.90-3.84 (m, 1H), 2.94 (s, 3H), 2.12 (s, 3H), 2.09 (s, 3H), 2.08 (s, 3H); ^{13}C-NMR (101 MHz, $CDCl_3$) δ (ppm) 170.65, 169.67, 169.49, 161.36, 89.77, 72.40, 71.95, 69.09, 68.61, 63.15, 31.11, 20.99, 20.88, 20.77; HRMS (m/z): [M + H]+ calculated for $C_{14}H_{21}N_2O_7S$: 361.1069; found 361.1064; [M+Na]+ calculated for $C_{14}H_{20}NaN_2O_7S$: 383.0889; found 383.0882; [M+K]+ calculated for $C_{14}H_{20}KN_2O_7S$: 399.0628; found 399.0620.

3,4,6,-*tri-O*-acetyl-1,2-dideoxy-2'-dimethylamino-α-D-glucopyranoso[2,1-d]-Δ2'- thiazoline (14b): ^{1}H-NMR (400 MHz, $CDCl_3$) δ (ppm) 6.24 (d, J = 6.6 Hz, 1H), 5.41 (dd, J = 4.3, 3.4 Hz, 1H), 4.95 (ddd, J = 9.6, 3.3, 0.9 Hz, 1H), 4.35 (dd, J = 6.1, 4.9 Hz, 1H), 4.15, (dd, J = 8.5, 5.8 Hz, 2H), 3.89 (ddd, J = 9.1, 5.1, 3.1 Hz, 1H), 3.01 (s, 6H), 2.11 (s, 3H), 2.09 (s, 3H), 2.07 (s, 3H); ^{13}C-NMR (101 MHz, $CDCl_3$) δ (ppm) 170.63, 169.62, 169.51, 162.50, 90.17, 72.84, 72.30, 69.10, 68.53, 63.11, 39.90, 21.00, 20.86, 20.76; HRMS (m/z): [M + H]+ calculated for $C_{15}H_{23}N_2O_7S$: 375.1226; found 375.1221; [M + Na]+ calculated for $C_{15}H_{22}NaN_2O_7S$: 397.1045; found 397.1037.

3,4,6,-*tri*-*O*-acetyl-1,2-dideoxy-2'[(2-propyl)amino]-α-D-glucopyranoso[2,1-d]-Δ2'-thiazoline (14d): ^{1}H NMR (400 MHz, $CDCl_3$) δ 6.28 (d, J = 6.5 Hz, 1H), 5.43 (dd, J = 4.3, 3.0 Hz, 1H), 4.98 (ddd, J = 9.4, 3.0, 1.0 Hz, 1H), 4.40 (ddd, J = 6.5, 4.3, 1.1 Hz, 1H), 4.21 – 4.15 (m, 2H), 3.90 (dt, J = 8.9, 4.2 Hz, 1H), 3.27 (ddt, J = 27.4, 13.1, 6.7 Hz, 2H), 2.15 (s, 3H), 2.12 (s, 3H), 2.11 (s, 3H), 1.71 – 1.61 (m, 2H), 0.99 (t, J = 7.4 Hz, 3H). ^{13}C NMR (101 MHz, $CDCl_3$) δ 170.53, 169.68, 169.44, 162.21, 87.67, 70.81, 69.56, 68.11, 66.18, 62.84, 29.70, 22.68, 20.90, 20.85, 20.73, 11.24. HRMS (m/z): [M + H]+ calculated for $C_{16}H_{25}N_2O_7S$: 389.1382 (M+H); found 389.1383; [M + Na]+ calculated for $C_{16}H_{24}NaN_2O_7S$: 411.1202; found 411.1199.

3,4,6,-*tri*-*O*-acetyl-1,2-dideoxy-2'[(2-butyl)amino]-α-D-glucopyranoso[2,1-d]-Δ2'-thiazoline (14e): ^{1}H NMR (500 MHz, $CDCl_3$) δ 6.29 (d, *J* = 6.5 Hz, 1H), 5.42 (dd, *J* = 4.4, 3.2 Hz, 1H), 5.01 – 4.91 (m, 1H), 4.46 – 4.33 (m, 2H), 4.19 (dd, *J* = 4.2, 2.8 Hz, 2H), 3.91 (ddd, *J* = 8.9, 5.2, 3.3 Hz, 1H), 3.41 – 3.25 (m, 2H), 2.15 (s, 3H), 2.12 (s, 3H), 2.11 (s, 3H), 1.62 (p, *J* = 7.2 Hz, 2H), 1.43 – 1.36 (m, 2H), 0.96 (t, *J* = 7.4 Hz, 3H). ^{13}C NMR (101 MHz, $CDCl_3$) δ 170.50, 169.85, 169.60, 162.30, 92.67, 71.75, 69.26, 68.33, 66.21, 62.08, 31.60, 20.93, 20.82, 20.66, 19.95, 16.88, 13.67. δ (ppm); HRMS (m/z): [M + H]+ calculated for $C_{17}H_{27}N_2O_7S$: 403.1539; found 403.1535.

3,4,6-*tri*-*O*-acetyl-1,2-dideoxy-2'-[(2-fluoroethyl)amino]-α-D-glucopyranoso[2,1-d]-Δ2'-thiazoline (14f): ^{1}H-NMR (400 MHz, $CDCl_3$) δ (ppm) 6.25 (d, J = 6.5 Hz, 1H), 5.40 (dd, *J* = 3.9, 2.6 Hz, 1H), 4.97 – 4.93 (m, 1H), 4.71 – 4.44 (m, 2H), 4.37 – 4.33 (m, 1H), 4.16 – 4.11 (m, 2H), 3.85 – 3.78 (m, 1H), 3.73 – 3.46 (m, 2H), 2.11 (s, 3H), 2.08 (s, 3H), 2.06 (s, 3H); ^{13}C-NMR (101 MHz, $CDCl_3$) 170.75, 169.68, 169.59, 159.80, 90.02, 82.04 (d, *J* = 166.8 Hz), 72.73, 71.78, 69.17, 68.62, 63.35, 44.59 (d, *J* = 20.2 Hz), 21.08, 20.90, 20.85; LRMS (m/z): [M + H]+ calculated for $C_{15}H_{22}FN_2O_7S$: 393.1132; found 393.1122.

3,4,6-*tri*-*O*-acetyl-1,2-dideoxy-2'-[(2,2-difluoroethyl)amino]-α-D-glucopyranoso[2,1-d]-Δ2'-thiazoline (14g): ^{1}H NMR (400 MHz, $CDCl_3$) δ 6.25 (d, *J* = 6.5 Hz, 1H), 6.02 (tdd, *J* = 56.6, 5.0, 3.5 Hz, 1H), 5.37 (dd, *J* = 3.9, 2.5 Hz, 1H), 4.93 (ddd, *J* = 9.4, 2.4, 1.1 Hz, 1H), 4.34 (ddd, *J* = 6.5, 4.0, 1.1 Hz, 1H), 4.14 – 4.11 (m, 2H), 3.81 – 3.66 (m, 2H), 3.59 – 3.45 (m, 1H), 2.10 (s, 3H), 2.06 (s, 3H), 2.05 (s, 3H). ^{13}C-NMR (101 MHz, $CDCl_3$) δ (ppm) 170.66, 169.56, 169.52, 159.57, 113.37 (t, *J* = 241.4 Hz), 90.31, 72.37, 71.44, 68.99, 68.55, 63.29, 46.10 (t, *J* = 27.2 Hz), 20.96, 20.73, 20.72; HRMS (m/z): [M+H]+ calculated for $C_{15}H_{21}F_2N_2O_7S$: 411.1038; found 411.1033; [M+Na]+ calculated for $C_{15}H_{20}F_2NaN_2O_7S$: 433.0857; found 433.0850.

3,4,6-*tri*-*O*-acetyl-1,2-dideoxy-2'-[(2,2,2-trifluoroethyl)amino]-α-D-glucopyranoso[2,1-d]-Δ2'-thiazoline (14h): ^{1}H NMR (500 MHz, $CDCl_3$) δ 6.22 (d, *J* = 6.6 Hz, 1H), 5.34 (dd, *J* = 3.7, 2.3 Hz, 1H), 4.91 – 4.84 (m, 1H), 4.33 – 4.28 (m, 1H), 4.09 – 3.99 (m, 3H), 3.80 (td, *J* = 9.0, 6.3 Hz, 1H), 3.76 – 3.72 (m, 1H), 2.07 (s, 3H), 2.03 (s, 3H), 2.01 (s, 3H). ^{13}C-NMR (126 MHz, $CDCl_3$) δ (ppm) 170.59, 169.70, 169.40, 159.58, 125.19 (q, *J* = 279.2 Hz), 89.61, 71.59, 71.19, 68.82, 68.19, 63.10, 44.98, 20.74, 20.54, 20.50; HRMS (m/z): [M+H]+ calculated for $C_{17}H_{20}F_3N_2O_9S$: 429.0943; found 429.0941; [M+Na]+ calculated for $C_{17}H_{19}F_3NaN_2O_9S$: 451.0763; found 451.0756.

Deprotection of amino thiazoline 10a. Per-acetylated intermediate **10a** (114 mg, 0.20 mmol) was dissolved in 2 mL of MeOH, followed by the addition of NaOMe (14 mg, 0.25 mmol). The reaction was then stirred at room temperature for 2 hours, and subsequently quenched by the addition of AcOH. Concentration gave a colorless oil which was dissolved in 3 mL of pyridine and then 0.6 mL of piperidine was added. The resulting mixture was then stirred at room temperature for 2 hours, concentrated, and any remaining piperidine was then co-evaporated with pyridine. The resulting mixture was triturated with EtOAc to yield inhibitor **11a** (38 mg, 80% yield).

1,2-dideoxy-2'-amino-α-D-glucopyranoso[2,1-d]-Δ2'-thiazoline (11a): ^{1}H NMR (500 MHz, CD_3OD) δ 6.31 (d, *J* = 6.4 Hz, 1H), 4.04 (t, *J* = 6.1 Hz, 1H), 3.91 (t, *J* = 5.3 Hz, 1H), 3.78 (dd, *J* = 11.7, 2.1 Hz, 1H), 3.67 – 3.57 (m, 2H), 3.47 (dd, *J* = 9.0, 5.0 Hz, 1H). ^{13}C NMR (125 MHz, CD_3OD) δ 173.50, 89.62, 77.75, 74.92, 69.74, 65.15, 63.26. Anal. Calcd. for $C_7H_{12}N_2O_4S$: C, 38.17; H, 5.49; N, 12.72; Found: C, 38.05; H, 5.37; N, 12.66.

General procedure for the deprotection of amino thiazolines 10b and 14a-h. A catalytic amount of K_2CO_3 (~5% w/v) was added to a solution of each per-acetylated 2'-aminothiazoline (**10b, 14a-h**) in anhydrous methanol. The reaction was stirred at room temperature under an atmosphere of argon for ~1-2 hours until judged complete by TLC analysis. The solution was filtered, and the solvent evaporated to yield the crude product which was then purified by silica flash chromatography (10-18% MeOH/DCM) to yield the final product. Products were isolated in yields ranging from 61-86%.

1,2-dideoxy-2'-[(2-prop-1-ene)amino]-α-D-glucopyranoso[2,1-d]-Δ2'-thiazoline (11b): ^{1}H NMR (500 MHz, CD_3OD) δ 6.33 (d, *J* = 6.3 Hz, 1H), 5.92 (ddt, *J* = 15.7, 10.6, 5.4 Hz, 1H), 5.27 – 5.20 (m, 1H), 5.18 – 5.10 (m, 1H), 4.08 (t, J = 6.1 Hz, 1H), 3.96 – 3.92 (m, 1H), 3.91 – 3.86 (m, 2H), 3.80 (dd, *J* = 11.7, 2.0 Hz, 1H), 3.69 – 3.60 (m, 2H), 3.49 (dd, *J* = 8.9, 5.3 Hz, 1H); ^{13}C-NMR (125 MHz, CD_3OD) δ (ppm) 162.15, 134.33, 114.87, 89.45, 75.07, 74.10, 73.83, 69.77, 61.87, 45.85; HRMS (m/z): [M+H]+ calculated for $C_{10}H_{17}N_2O_4S$: 261.0909; found 261.0908; [M+Na]+ calculated for $C_{10}H_{16}NaN_2O_4S$: 283.0728; found 283.0727.

1,2-dideoxy-2'-aminomethyl-α-D-glucopyranoso[2,1-d]-Δ2'-thiazoline (15a): ^{1}H-NMR (400 MHz, D_2O) δ (ppm) 6.21 (d, *J* = 7.7 Hz, 1H), 4.11 (t, *J* = 5.9 Hz, 1H), 3.96 (t, *J* = 5.0 Hz, 1H), 3.73 (t, *J* = 7.7 Hz, 1H), 3.58 (dt, *J* = 10.0, 7.1 Hz, 2H), 3.49 (dd, *J* = 9.0, 4.5 Hz, 1H), 2.74 (s, 3H); ^{13}C-NMR (101 MHz, D_2O) δ (ppm) 163.75, 88.54, 74.25, 73.64, 73.28, 69.31, 61.42, 29.87 HRMS (m/z): [M+H]+ calculated for $C_8H_{15}N_2O_4S$: 235.0753; found 235.0750; [M+Na]+ calculated for $C_8H_{14}NaN_2O_4S$: 257.0572; found 257.0570.

1,2-dideoxy-2'-dimethylamino-α-D-glucopyranoso [2,1-d]-Δ2'-thiazoline (15b): ^{1}H-NMR (400 MHz, CD_3OD) δ (ppm) 6.39 (d, *J* = 6.5 Hz, 1H), 4.09 (t, *J* = 6.3 Hz, 1H), 3.90 (t, *J* = 5.9 Hz, 1H), 3.84-3.78 (m, 1H), 3.72-3.61 (m, 2H), 3.52-3.45 (m, 1H), 3.06 (s, 6H); ^{13}C-NMR (125 MHz, CD_3OD) δ (ppm) 164.30, 90.59, 75.02, 74.84. 74.45, 69.72, 61.81, 38.67; HRMS (m/z): [M + H]+ calculated for $C_9H_{17}N_2O_4S$: 249.0909; found 249.0908; [M+Na]+ calculated for $C_9H_{16}NaN_2O_4S$: 271.0728; found 271.0725.

1,2-dideoxy-2'-[(2-ethyl)amino]-α-D-glucopyranoso [2,1-d]-Δ2'-thiazoline (15c) (ThiamEtG): Our characterization data matched what was established by Yuzwa *et al.*[2]

1,2-dideoxy-2'-[(2-propyl)amino]-α-D-glucopyranoso [2,1-d]-Δ2'-thiazoline (15d): ^{1}H-NMR (500 MHz, CD_3OD) δ (ppm) 6.58 (d, *J* = 6.5 Hz, 1H), 4.13 (t, *J* = 6.6 Hz, 1H), 3.91-3.79 (m, 2H), 3.76-3.63 (m, 2H), 3.50 (dd, *J* = 8.8, 6.9 Hz, 1H), 1.70-1.62 (m, 2H), 0.98 (t, *J* = 7.4 Hz, 3H); ^{13}C-NMR (125 MHz, $(CD_3)_2CO$) δ (ppm) 182.84, 93.02, 79.42, 75.18, 68.88, 64.80, 63.16, 45.96, 21.08, 10.75; HRMS (m/z): [M+H]+ calculated for $C_{10}H_{19}N_2O_4S$: 263.1066; found 263.1063; [M+Na]+ calculated for $C_{10}H_{18}NaN_2O_4S$: 285.0885; found 285.0876.

1,2-dideoxy-2'-[(2-butyl)amino]-α-D-glucopyranoso [2,1-d]-Δ2'-thiazoline (15e): ^{1}H NMR (500 MHz, CD_3OD) δ 6.34 (d, *J* = 6.4 Hz, 1H), 4.07 (t, *J* =6.2 Hz, 1H), 3.92 (t, *J* = 5.8 Hz, 1H), 3.85 – 3.77 (m, 1H), 3.71 – 3.62 (m, 2H), 3.52 – 3.46 (m, 1H), 3.31 – 3.22 (m, 2H), 1.57 (p, *J* = 7.2 Hz, 2H), 1.41 (dt, *J* = 15.0, 7.4 Hz, 2H), 0.97 (t, *J* = 7.4 Hz, 3H); ^{13}C-NMR (125 MHz, CD_3OD, δ (ppm) 163.07, 89.08, 75.17, 74.17, 72.75, 69.55, 61.73, 43.59, 31.00, 19.69, 12.72.; HRMS (m/z): [M+H]+ calculated for $C_{11}H_{20}N_2O_4S$: 277.1222 (M+H); found 277.1167; [M+Na]+ calculated for $C_{11}H_{20}NaN_2O_4S$: 299.1041; found 299.1040.

1,2-dideoxy-2'-[(2-fluoroethyl)amino]-α-D-glucopyranoso[2,1-d]-Δ2'-thiazoline (15f): ^{1}H-NMR (400 MHz, CD_3OD) δ (ppm) 6.34 (d, *J* = 6.4 Hz, 1H), 4.60-4.49 (m, 1H), 4.48-4.37 (m, 1H), 4.05 (t, *J* = 6.2 Hz, 1H), 3.88 (t, *J* = 5.8 Hz, 1H), 3.76 (dd, *J* = 11.7, 2.1 Hz, 1H), 3.66-3.43 (m, 5H); ^{13}C-NMR (100 MHz, CD_3OD) δ (ppm) 163.45, 89.20, 81.57 (d, *J* = 167.4 Hz), 75.23, 73.93, 72.20, 69.40, 61.63, 44.16 (d, *J* = 20.3 Hz); HRMS (m/z): [M+H]+ calculated for $C_9H_{16}N_2O_4SF$: 267.0815; found 267.0822.

1,2-dideoxy-2'-[(2,2-difluoroethyl)amino]-α-D-glucopyranoso[2,1-d]-Δ2'-thiazoline (15g): ^{1}H NMR (500 MHz, CD_3OD) δ 6.29 (d, *J* = 6.4 Hz, 1H), 5.96 (tt, *J* = 56.3, 4.3 Hz, 1H), 4.04 (t, *J* = 6.1 Hz, 1H), 3.89 (t, *J* = 5.4 Hz, 1H), 3.74 (dd, *J* = 11.9, 2.3 Hz, 1H), 3.64 – 3.41 (m, 5H). ^{13}C-NMR (125 MHz, D_2O) δ (ppm) 162.48, 113.81 (t, *J* = 239.6 Hz), 88.11, 73.79, 72.58, 68.78, 61.00, 48.39, 44.95 (t, *J* = 25.0 Hz); HRMS (m/z): [M+H]+ calculated for $C_9H_{15}N_2O_4SF_2$: 285.0715; found 285.0717; [M+Na]+ calculated for $C_9H_{14}NaN_2O_4SF_2$: 307.0540; found 307.0536.

1,2-dideoxy-2'-[(2,2,2-trifluoroethyl)amino]-α-D-glucopyranoso[2,1-d]-Δ2'-thiazoline (15h): ^{1}H-NMR (500 MHz, CD_3OD) δ (ppm) 6.34 (d, *J* = 6.4 Hz, 1H), 4.04-3.99 (m, 1H), 3.98-3.85 (m, 3H), 3.78 (dd, *J* = 11.9, 2.2 Hz, 1H), 3.65 (dd, *J* = 11.9, 6.1 Hz, 1H), 3.59 (ddd, *J* = 8.5, 6.2, 2.1 Hz, 1H), 3.47 (dd, *J* = 9.1, 3.5 Hz, 1H); ^{13}C NMR (151 MHz, D_2O) δ 162.61, 123.97 (q, *J* = 278.5 Hz), 88.09, 73.84, 72.63, 68.68, 60.91, 44.10; HRMS (m/z): [M+H]+ calculated for $C_9H_{14}N_2O_4SF_3$: 303.0626; found 303.0622; [M+Na]+ calculated for $C_9H_{13}NaN_2O_4SF_3$: 325.0446; found 325.0440.

General procedure for the synthesis of 4-methylumbelliferyl 2-deoxy-2-acetamido-D-glucopyranosides. 4-Methylumbelliferyl 2-amino-2-deoxy-β-D-glucopyranoside hydrochloride (**16**) was synthesized according to the procedure described by Roeser and Leger, and was used without further purification.[3] To a solution of the hydrochloride salt **16** (250 mg, 0.5 mmol) in CH_3CN (5 ml) was added triethylamine (2.0 eq.) and the appropriate alkyl isocyanate (2.0 eq. [for **17a**, TMSNCO was used]) at 0° C and the solution left to stir at room temperature (3 h). The reaction was then diluted with EtOAc (25 ml) and washed with water (25 ml), $NaHCO_3$ (25 ml), brine (10 ml), dried (MgSO4), filtered and concentrated. The resultant solids were then deprotected using the procedure of Macauley *et al*[4] to give the corresponding triols **17a-e** in yields ranging from 49% - 63% over two steps.

General procedure for the synthesis of de-protected substrates 18a-e. All substrates were deprotected by following the procedure described for the deprotection of 4-methylumbelliferyl substrates synthesized by Macauley *et al.*[5]

4-Methylumbelliferyl-2-[(amino)oxoamino]-2-deoxy-β-D-glucopyranose (18a): ^{1}H NMR (600 MHz, DMSO-d_6) δ 8.60 (s, 1H), 7.71 (dd, *J* = 8.8, 3.6 Hz, 1H), 7.03 – 6.94 (m, 2H), 6.74 (s, 1H), 6.26 (d, *J* = 1.6 Hz, 1H), 6.04 (d, *J* = 8.2 Hz, 1H), 5.51 (s, 1H), 5.34 (d, *J* = 8.4 Hz, 1H), 5.21 (d, *J* = 8.2 Hz, 1H), 4.64 (s, 1H), 3.73 (d, *J* = 11.1 Hz, 1H), 3.66 (q, *J* = 9.2 Hz, 1H), 3.47 (q, *J* = 8.1, 5.8 Hz, 3H), 3.20 (q, *J* = 9.4 Hz, 1H), 2.41 (dd, *J* = 2.5, 1.3 Hz, 3H). ^{13}C NMR (151 MHz, DMSO-d_6) δ 160.16, 160.01, 158.86, 154.38, 153.25, 126.50, 114.23, 113.54, 111.77, 103.17, 98.99, 77.14, 74.69, 70.47,

60.64, 55.67, 18.11. HRMS (m/z): [M+H]+ calculated for $C_{17}H_{21}N_2O_8$: 381.1298; found 381.1293; [M+Na]+ calculated for $C_{17}H_{20}N_2NaO_8$: 403.1117; found 403.1111.

4-Methylumbelliferyl-2-[(aminomethyl)oxomethyl]amino]-2-deoxy-2-β-D-glucopyranose (18b): ^{1}H NMR (600 MHz, DMSO-d_6) δ 7.71 (d, *J* = 8.8 Hz, 1H), 7.02 – 6.94 (m, 2H), 6.26 (d, *J* = 1.5 Hz, 1H), 5.96 (d, *J* = 8.4 Hz, 1H), 5.81 (d, *J* = 4.9 Hz, 1H), 5.20 (d, *J* = 8.3 Hz, 1H), 5.11 (dd, *J* = 17.7, 5.3 Hz, 2H), 4.62 (t, *J* = 5.8 Hz, 1H), 3.73 (dd, *J* = 11.4, 5.4 Hz, 1H), 3.54 – 3.38 (m, 3H), 3.20 – 3.16 (m, 2H), 2.55 (d, *J* = 4.6 Hz, 3H), 2.41 (d, *J* = 1.3 Hz, 3H). ^{13}C NMR (151 MHz, DMSO-d_6) δ 160.16, 160.04, 158.72, 154.38, 153.27, 126.43, 114.12, 113.54, 111.72, 103.16, 99.03, 77.20, 74.70, 70.45, 60.70, 56.34, 26.42, 18.11. HRMS (m/z): [M+H]+ calculated for $C_{18}H_{23}N_2O_8$: 395.1454; found 395.1449; [M+Na]+ calculated for $C_{18}H_{22}N_2NaO_8$: 417.1274; found 417.1269.

4-Methylumbelliferyl-2-[(aminoethyl)oxoethyl]amino]-2-deoxy-β-D-glucopyranose (18c): ^{1}H NMR (600 MHz, DMSO-d_6) δ 7.71 (d, *J* = 8.8 Hz, 1H), 7.00 – 6.95 (m, 2H), 6.26 (d, *J* = 1.5 Hz, 1H), 5.90 – 5.86 (m, 2H), 5.21 – 5.09 (m, 3H), 4.62 (t, *J* = 5.8 Hz, 1H), 3.73 (dd, *J* = 11.7, 5.1 Hz, 1H), 3.52 – 3.45 (m, 2H), 3.41 (dd, *J* = 9.5, 5.8 Hz, 1H), 3.24 – 3.15 (m, 1H), 3.02 (dd, J = 7.5, 6.0 Hz, 2H), 2.63 (d, *J* = 2.4 Hz, 1H), 2.42 – 2.39 (m, 3H), 0.99 (td, *J* = 7.2, 1.3 Hz, 3H). ^{13}C NMR (151 MHz, DMSO-d_6) δ 160.19, 160.04, 158.07, 154.38, 153.28, 126.43, 114.28, 113.67, 111.47, 103.09, 99.09, 77.21, 74.65, 70.47, 60.71, 56.26, 34.13, 18.11, 15.61. HRMS (m/z): [M+H]+ calculated for $C_{19}H_{25}N_2O_8$: 409.1608; found 409.1611; [M+Na]+ calculated for $C_{18}H_{22}N_2NaO_8$: 431.1430; found 431.1425.

4-Methylumbelliferyl-2-[(aminopropyl)oxopropyl]amino]-2-deoxy-β-D-glucopyranose (18d): ^{1}H NMR (600 MHz, DMSO-d_6) δ 7.71 (dd, *J* = 8.9, 1.4 Hz, 1H), 7.02 – 6.94 (m, 2H), 6.26 (t, *J* = 1.3 Hz, 1H), 5.94 – 5.85 (m, 2H), 5.20 – 5.09 (m, 3H), 4.63 (t, *J* = 5.7 Hz, 1H), 3.76 – 3.70 (m, 1H), 3.55 – 3.45 (m, 2H), 3.42 (tt, *J* = 8.1, 4.6 Hz, 2H), 3.19 (s, 1H), 2.95 (ddd, *J* = 13.0, 10.4, 6.8 Hz, 2H), 2.41 (t, *J* = 1.4 Hz, 3H), 1.37 (q, *J* = 7.3 Hz, 2H), 0.82 (td, *J* = 7.4, 1.6 Hz, 3H). ^{13}C NMR (151 MHz, DMSO-d_6) δ 160.17, 160.04, 158.19, 154.38, 153.25, 126.42, 114.12, 113.51, 111.71, 103.17, 99.23, 77.21, 74.64, 70.48, 60.72, 56.27, 41.12, 23.15, 18.11, 11.31. HRMS (m/z): [M+H]+ calculated for $C_{20}H_{27}N_2O_8$:423.1767; found 423.1769; [M+Na]+ calculated for $C_{20}H_{26}N_2NaO_8$: 445.1587; found 445.1586.

4-Methylumbelliferyl-2-[(aminobutyl)oxobutyl]amino]-2-deoxy-β-D-glucopyranose (18e): ^{1}H NMR (600 MHz, DMSO-d_6) δ 7.71 (d, *J* = 8.8 Hz, 1H), 7.00 – 6.94 (m, 2H), 6.26 (d, *J* = 1.3 Hz, 1H), 5.90 – 5.84 (m, 2H), 5.20 (d, *J* = 8.3 Hz, 1H), 5.12 (dd, *J* = 21.0, 5.3 Hz, 2H), 4.63 (t, *J* = 5.7 Hz, 1H), 3.73 (dd, *J* = 11.6, 5.1 Hz, 1H), 3.54 – 3.45 (m, 2H), 3.19 (td, *J* = 9.1, 5.1 Hz, 1H), 2.99 (dp, *J* = 19.3, 6.5 Hz, 2H), 2.62 (d, *J* = 1.9 Hz, 1H), 2.41 (d, *J* = 1.3 Hz, 3H), 1.30 (dp, *J* = 47.8, 7.1 Hz, 4H), 0.85 (dd, *J* = 7.8, 6.8 Hz, 3H). ^{13}C NMR (151 MHz, DMSO-d_6) δ 160.21, 160.03, 158.16, 154.37, 153.27, 126.40, 114.20, 113.54, 111.73, 102.89, 99.24, 77.21, 74.60, 70.48, 60.72, 56.29, 38.96, 32.13, 19.48, 18.06, 13.71. HRMS (m/z): [M+H]+ calculated for $C_{21}H_{29}N_2O_8$: 437.1924; found 437.1921; [M+Na]+ calculated for $C_{21}H_{28}N_2NaO_8$: 459.1743; found 459.1738.

Biological evaluation: Gene expression and protein purification. hOGA was expressed and purified according to the previously described procedure by Macauley *et al.*[5] hOGA was dialyzed twice against PBS buffer after purification and its concentration determined using the $Nanodrop_{2000}$. Human β-hexosaminidase was obtained from Micheal Tropak at the Hospital for Sick Children in Toronto, without further purification.

Kinetic analysis of hOGA. All assays were carried out in 96 well plates (Thermoscientific, FloroNunc, lot # 139825) in PBS pH 7.4 buffer (0.03% BSA) in triplicate at 37°C for 20 minutes. A continuous assay procedure was performed in which the reactions were initiated by the addition of 4-MUGlcNAc substrate (50 µL or 100 µL via a multi-channel pipette). The total assay volume was 150 µL for the K_i determination or 200 µL for the K_m/V_{max} determination. The progress at the end of the reaction was determined by assessing the extent of 4-methylumbelliferone liberation as determined by fluorescence measurements using a BioTek Synergy Plate Reader. The excitation and emission were 350 and 445 nm, respectively. The amount of fluorogenic substrate liberated was quantitatively assessed by using a standard curve for 4-methylumbelliferone under identical buffer conditions. A total of 6-8 inhibitor concentrations were tested, ranging from 1/5 to 5 times the K_i value. The hOGA concentrations in the assays ranged from 0.8 nM to 20 nM, depending on whether the K_i being determined was through classic Michaelis-Menten methods or the Morrison K_i fit. For full K_i analyses, 5 substrate concentrations were used ranging from 1/5 to 5 times the K_m value. For the Morrison K_i analyses, only one substrate concentration was used (at the K_m) since the method of inhibition was already known from analysis using the Line-weaver Burke plots. The representative hOGA K_i plots are summarized in Supplemental Figures S1, S2 and S3. Kinetic analysis of substrates **18a-e** were performed in triplicate using pH 7.4 PBS buffer (0.03% BSA) containing 2.5% DMF, with varying enzyme concentrations. The Michaelis-Menten curves for substrates **18a-e** with hOGA are summarized in Supplemental Figure S6 and the kinetic parameters are described in Supplemental Table S1.

Kinetic analysis of β-hexosaminidase. All assays were carried out in 96 well plates (Thermoscientific, FloroNunc, lot # 139825) at pH 4.25 (50 mM citrate, 100 mM NaCl, 0.03% BSA) at 37°C in triplicate for 20 minutes. Enzymatic reactions were triggered by addition of substrate (20 µL or 15 µL) in a total volume of 45 or 40 µL. Reactions were quenched by the addition of a 4-fold excess (160 or 180 µL) of quenching buffer (200 mM glycine, pH 10.75) in order to

detect the fluorescent signal. The progress at the end of the reaction was determined by assessing the extent of 4-methylumbelliferone liberation as determined by fluorescence measurements using a BioTek Synergy Plate Reader. The excitation and emission were 350 and 445 nm, respectively. A total of 6-8 inhibitor concentrations were tested, ranging from 1/5 to 5 times the K_i value. The hHexB concentrations in the assays ranged from 2 to 5 nM and the K_i values were assessed by linear regression of data from Dixon plot analysis. The representative Dixon plots are summarized in Supplemental Figure S5. Full K_i values were verified for **11a** and **15a** using the same conditions mentioned, except with 5 substrate concentrations ranging from 1/5 to 5 times the K_m value.

p*K*$_a$ determination using ^{13}C NMR. All titrations were carried out on the Bruker AVANCE II 600 MHz NMR spectrometer. The samples prepared for each titration contained 5 µL of 1,4-dioxane as an internal standard, an equal mol amount of inhibitor and 3-nitrophenol or 3.4-dinitrophenol (depending on the availability of each compound) in 500 µL of H_2O and 75 µL of D_2O. Previous studies by Perrin *et al* have reported no differences in chemical shifts for varying the compound concentration in the titration assay.[6] Each sample was made basic with 25-70 µL of 2M NaOH, and prior to titration, it was ensured that there was no change in chemical shift with further addition of base in 5 µL increments, in order to accurately establish the δ_d for each compound. The basic sample was then titrated with 10 µL of 0.5 M (**15f-15h, NButGT**) or 1 M HCl (**15c**) until there was no further change in chemical shift, indicating that the end point of the titration was reached. The point where the chemical shift stopped changing was the recorded δ_p for each compound. The relative pK_a (ΔpK_a) was then determined by non-linear fitting of data to Equation S1, which provides the value of R (R = K_a^x/K_a^r) and describes the relationship between the difference (Δ) in chemical shifts (δ) between each compound of unknown pK_a (x) and the reference compound with known pK_a (r). δ_p and δ_d represent the chemical shifts of the protonated and de-protonated species of each compound, where Δ_d expresses the difference between the de-protonated species ($\delta_d^x - \delta_d^r$), Δ^x represents the difference between the deprotonated and protonated species of x ($\delta_d^x - \delta_p^x$), and Δ^r for that of the reference compound ($\Delta^r = \delta_d^r - \delta_p^r$). The fractional protonation of the reference compound is represented by n and is equal to $(\delta - \delta_d)/(\delta_p - \delta_d)$.[7]

$$\Delta = \Delta_d + \frac{Rn(\Delta^x)}{Rn - n + 1} - n(\Delta^r) \qquad \textbf{(S1)}$$

The reference compound for titration of **15c-15g** was 3-nitrophenol (pK_a = 8.42)[8] and for NButGT and **15h** was 3,4-dinitrophenol (pK_a = 5.42). Both phenols have a single site of protonation in the pH range of interest and have ^{13}C resonances well separated from the resonances observed for the inhibitors. We observed the 2′-carbon of the aminothiazoline system and the C-1 carbon of 3-nitrophenol since these are most proximal to the ionizable center. Fitting of the resulting data to Equation S1 (Figure 5 (ThiamEt-G), Supplemental Figure S7) enabled us to obtain values of R for each compound and, by comparison to the reference compound, absolute pK_a values (Table 1). The observed pK_a values range from 7.68 to 4.65 and fall in the expected order with the conjugate acid of the trifluoromethyl-containing analogue (**15h**) being the most acidic of the aminothiazoline inhibitors. Supplemental Table S2 summarizes the specific chemical shifts used to determine the parameters outlined in Supplemental Table S4.

Structural analysis of *Bacteroides thetaiotaomicron* GH84 (BtGH84). Expression and purification of BtGH84 was carried out as described previously.[9] Crystals of the apo form were grown in sitting drop format, mixing 0.5 µl protein solution (12mg/ml) with 0.5 µl reservoir solution (0.1 M Imidazole pH 8, 3 % (w/v) trimethylamine N-oxide dehydrate, 10% (w/v) PEG 8000 15 % ethanediol).

For soaking experiments the ligand was dissolved in H_2O/DMSO (95%/5% (v/v)) at a concentration of 100 mM. Crystals of *apo*-BtGH84 were soaked in a solution containing the reservoir compounds with 2 % elevated PEG concentration, 25 % ethanediol as cryoprotectant and the respective inhibitor at a concentration of 10 mM. Subsequently, crystals were mounted using a Nylon fibre loop (Hampton Research) and flash frozen in liquid nitrogen. Data were collected at the Diamond light source beamline I04 at 100 K, using a Pilatus 6M-F detector or at the ESRF ID14.1 beamline, equipped with an ADSC Q210 CCD detector. The relevant data collections statistics can be found in Supplemental Table S3.

Data were processed using XDS[10] or Mosflm/SCALA[11, 12]. The indexing was chosen to be consistent with previous datasets using Pointless[13] part of the CCP4 software suite[14]. Initial electron density maps were obtained by direct refinement with an unliganded structure. Further model building and refinement was carried out by alternating cycles or reciprocal refinement using Refmac[15] or phenix refine[16] followed by manual rebuilding in COOT[17]. A model of the ligand and the respective library for refinement was generated in Jligand[18]. The ligand was included in the model followed by further rounds of refinement till convergence. The quality of the structure was evaluated using Molprobity[19] part of the Phenix software suite[16]. Figures were prepared using CCP4MG[20].

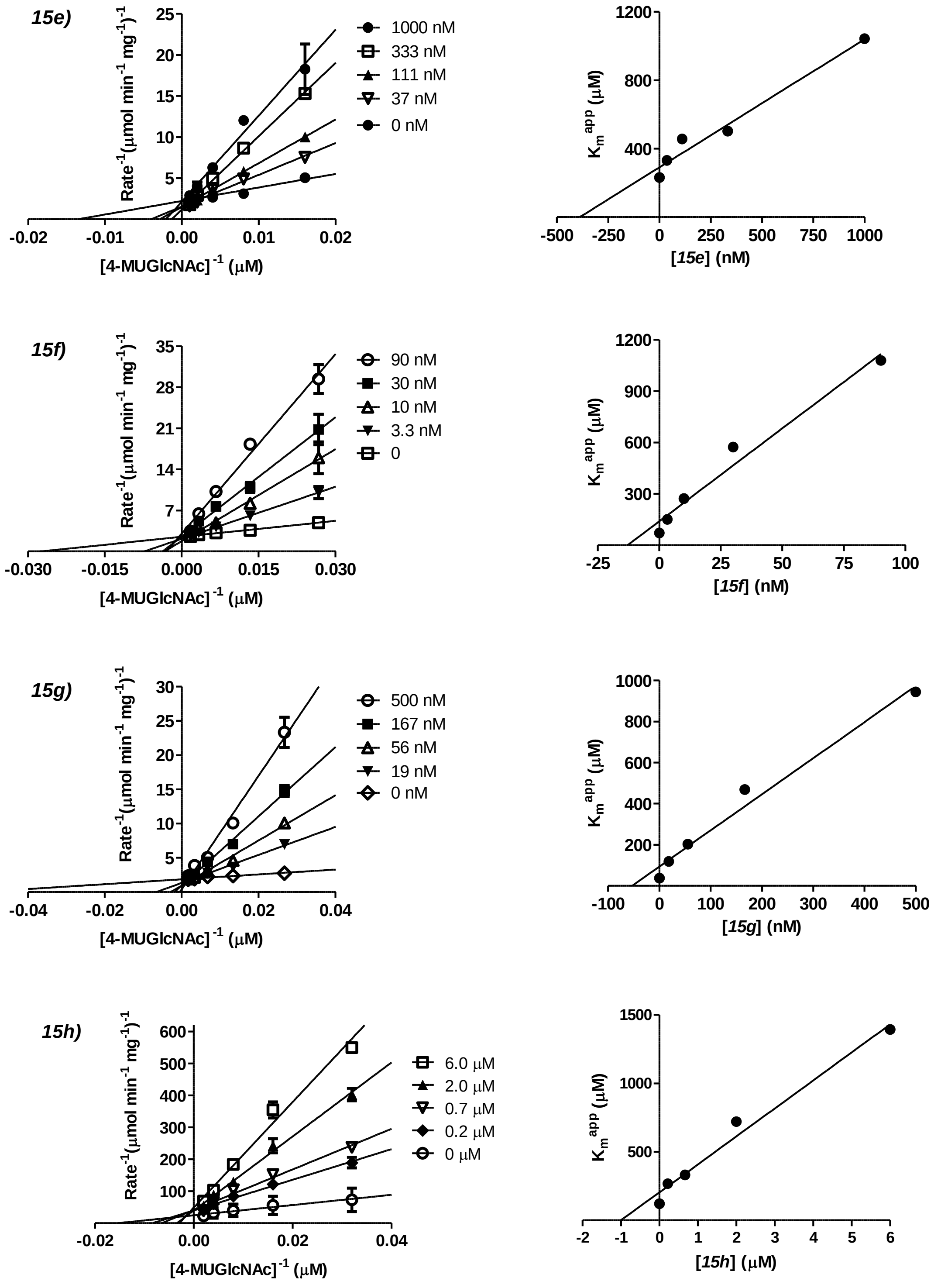

Figure S1. Lineweaver-Burke plots for inhibition of hOGA by compounds ***15e-15h*** (left panels) and the K_m^{app} vs. [I] plots for each inhibitor (right panels). The inhibitor concentrations used for each experiment are indicated in the legend of the K_i plots and the [hOGA] used in all cases was 1 nM.

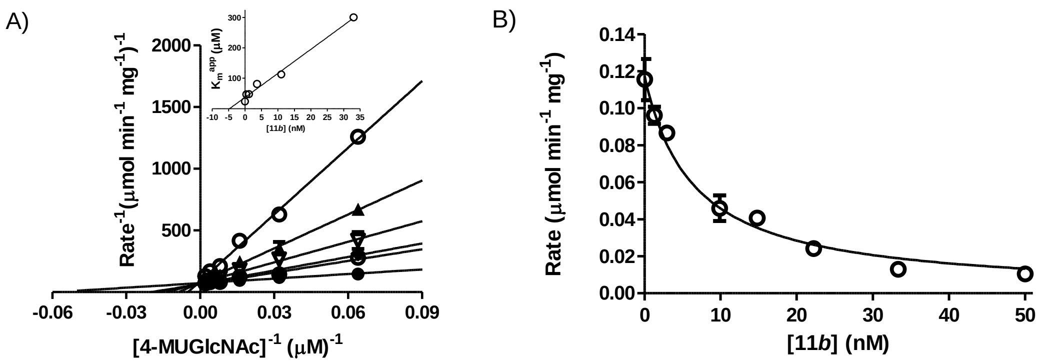

Figure S2. Comparison of K_i values determined for compound **11b** with hOGA using the Michaelis-Menten and Morrison approaches. A) Michaelis-Menten analysis of the inhibition of hOGA-catalyzed hydrolysis of 4-methylumbelliferyl 2-acetamido-2-deoxy-β-D-glucopyranoside (4-MUGlcNAc) reveals a pattern of competitive inhibition with a $K_i = 2.3 \pm 0.3$. The concentrations of **11b** used in the assays were 33.0, 11.0, 3.7, 1.2, 0.4 and 0.0 nM, and that of hOGA was 0.8 nM. **Inset:** graphical determination of the K_i value obtained by plotting K_m apparent (K_m^{app}) values against the concentration of **11b** affords a K_i value of 4.7 nM. B) Determination of the K_i value using the Morrison approach ($K_i = 3.2 \pm 0.4$). Concentrations of 11b started at 50 to 0.17 nM and were diluted by 1.5-fold. The substrate concentration used was the same as the K_m value determined for the hOGA enzyme batch (60 μM) and the concentration of hOGA used in the assay was 10 nM.

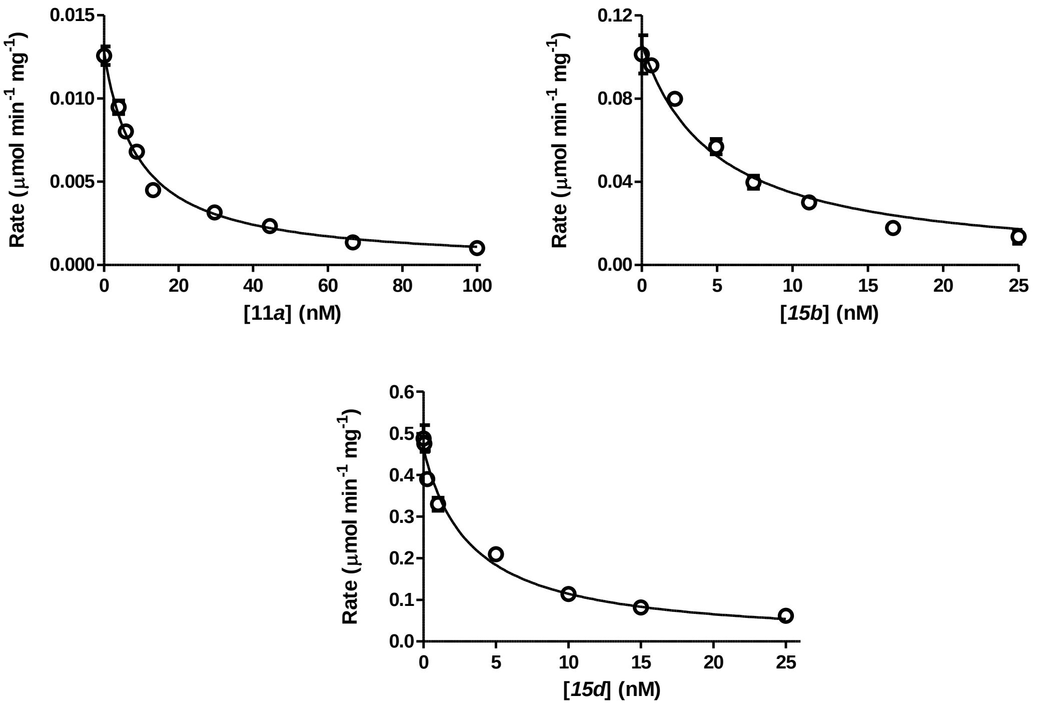

Figure S3. Morrison K_i plots for compounds ***11a, 15b*** and ***15d***. ***11a***: [hOGA] = 15 nM, [4-MUGlcNAc] = 170 μM; ***15b***: [hOGA] = 10 nM, [4-MUGlcNAc] = 60 μM; ***15d***: [hOGA] = 10 nM, [4-MUGlcNAc] = 180 μM.

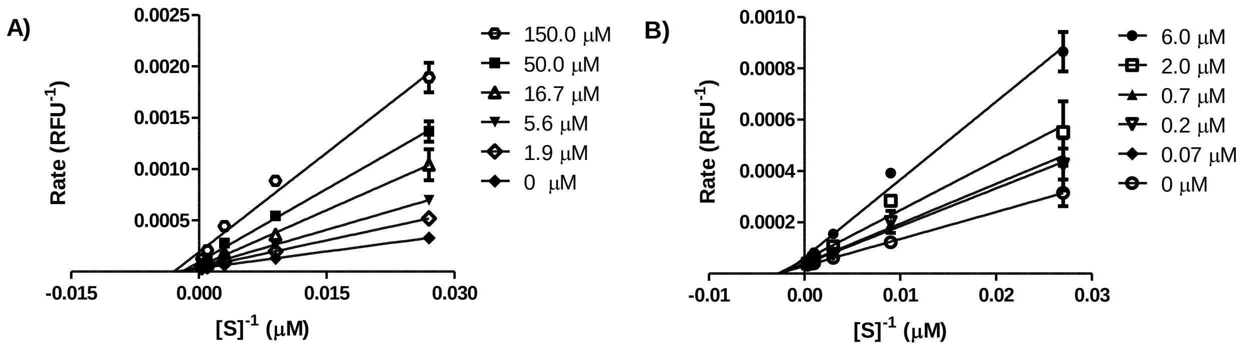

Figure S4. Lineweaver-Burke plots for inhibition of hHexB by compounds ***11a*** (panel A) and ***15a*** (panel B) using 4-MUGlcNAc as the substrate. Inhibitor concentrations are presented in each graph.

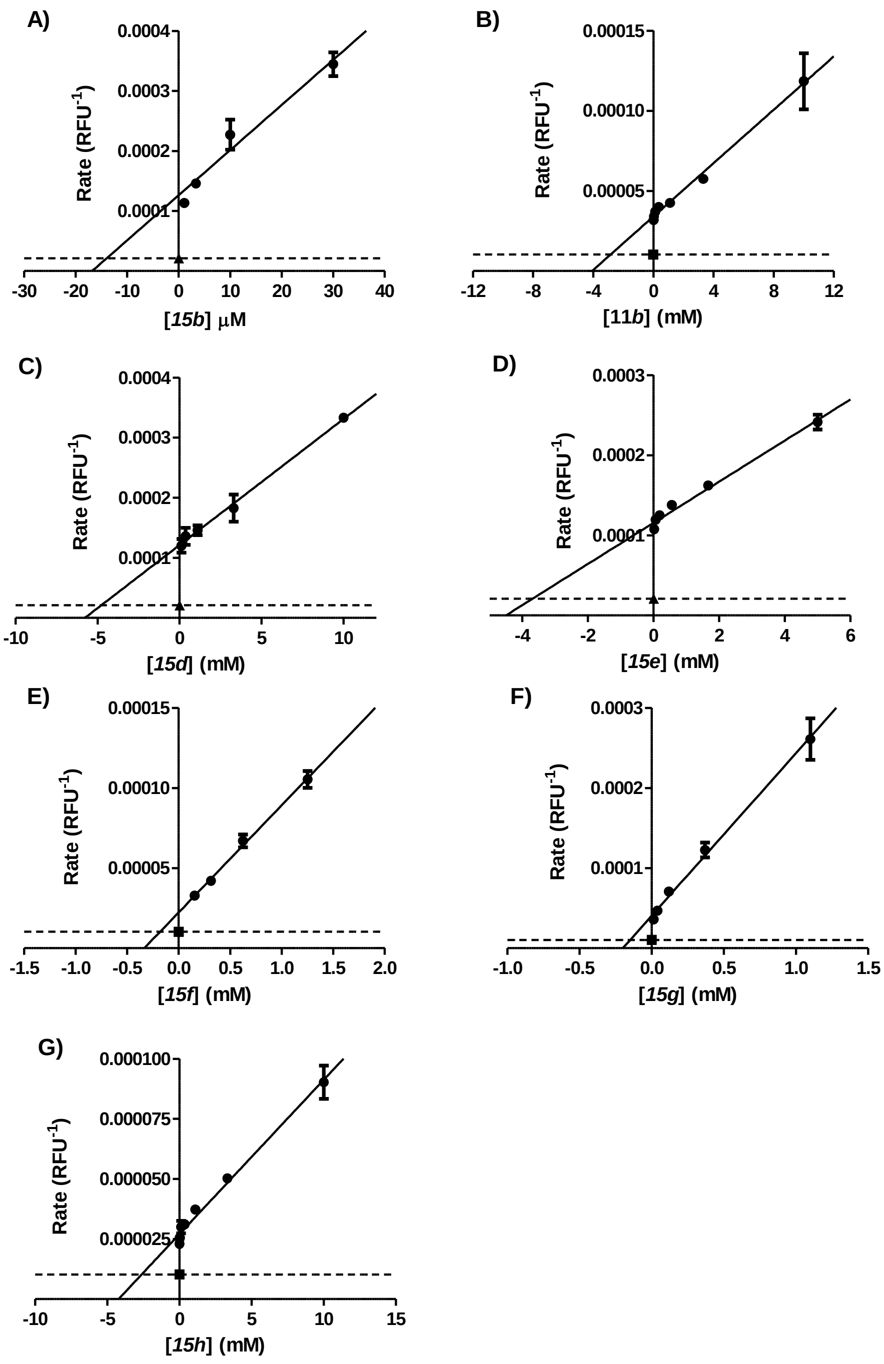

Figure S5. Dixon plot analysis of inhibition of hHexB by compounds ***11b***, ***15d-15h*** using 4-MUGlcNAc as a substrate. A) [E] = 5 nM, [S] = 230 µM; B) [E] = 5 nM, [S] = 500 µM; C) [E] = 2 nM, [S] = 60 µM; D) [E] = 2nM, [S] = 60 µM; E) [E] = 5 nM, [S] = 220 µM; F) [hHexB] = 5 nM, [4-MUGlcNAc] = 220 µM; G) [E] = 5 nM, [S] = 220 µM. Inhibitors were serially diluted 3-fold from the highest concentration. The K_i values were determined by assessing the point of intersection between the $1/V_{max}$ (dotted) line and the linear regression curve.

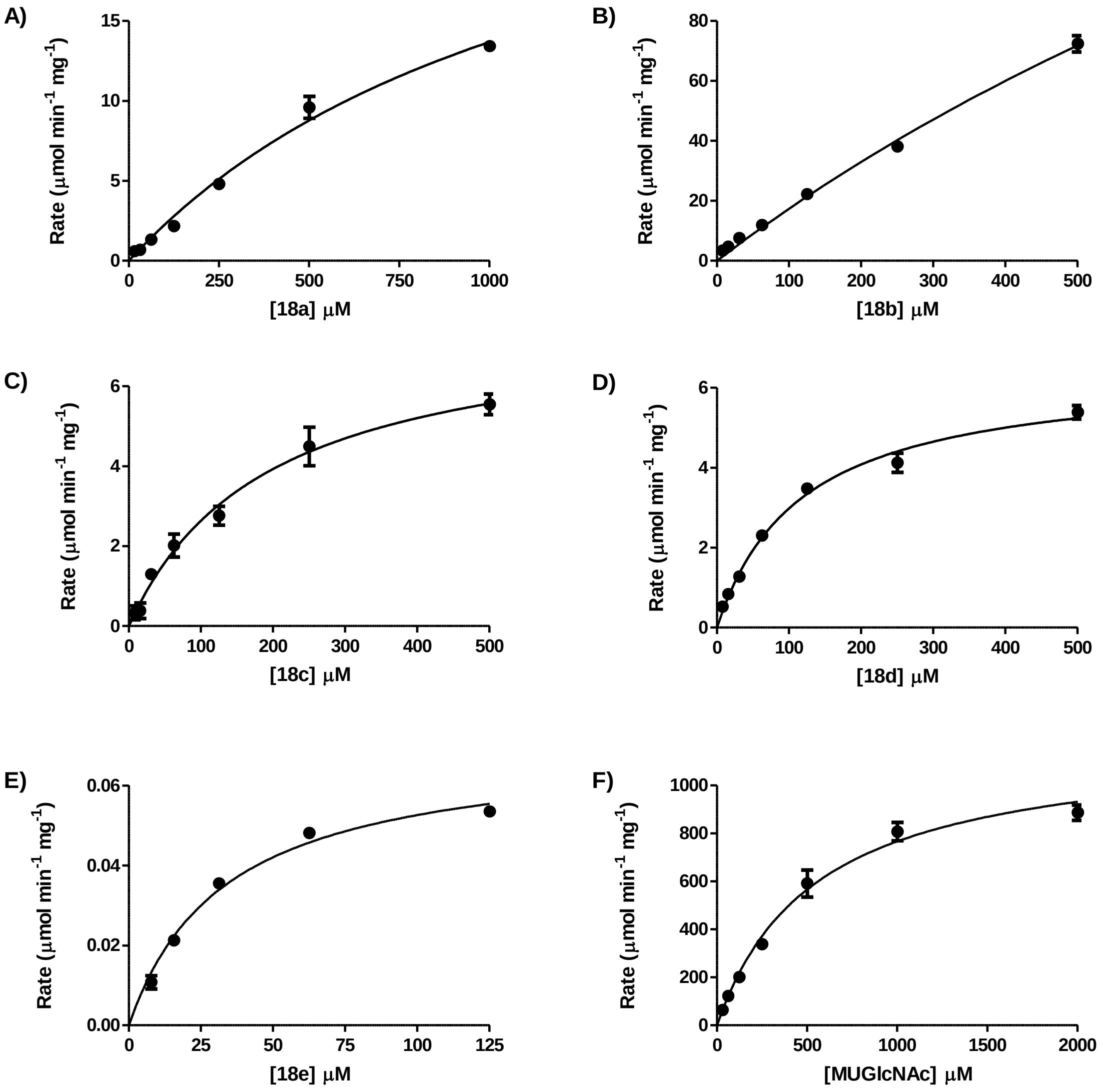

Figure S6. Michaelis-Menten plots obtained for urea substrates **18a-18e** and 4-MUGlcNAc with hOGA. All assays were performed in triplicate using pH 7.4 PBS buffer (0.03% BSA) containing 2.5% DMF. A) [E] = 0.1 μM B) [E] = 0.1 μM C) [E] = 0.2 μM D) [E] = 0.5 μM E) [E] = 12 μM F) [E] = 0.010 μM.

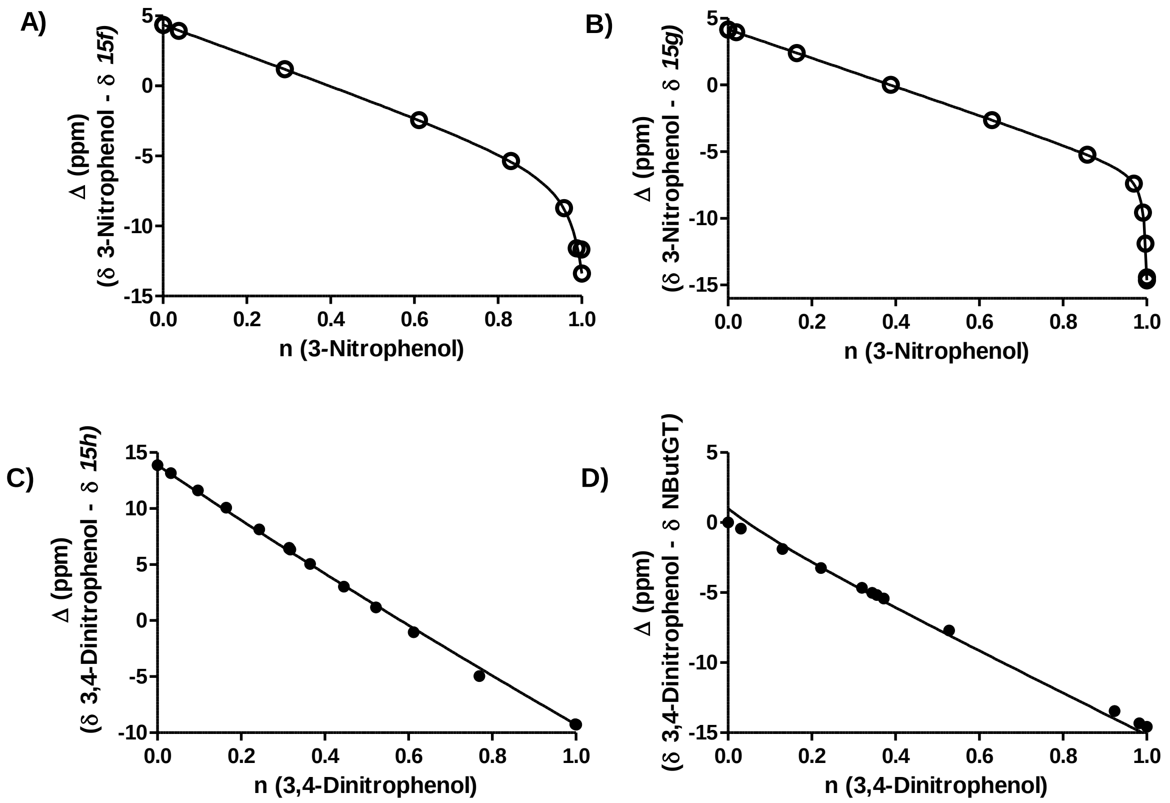

Figure S7. The difference between the chemical shifts of the thiazoline carbon resonances (Δ ppm) for ***15f*** **(A)** and ***15g*** **(B)** as a function of the fractional protonation (n) of 3-nitrophenol (pK_a = 8.42) and ***15h*** **(C)** and **NButGT (D)** as a function of the fractional protonation (n) of 3,4-dinitrophenol (pK_a = 5.42); In all cases, n = $(\delta\text{-}\delta_d)/(\delta_p\text{-}\delta_d)$ and the data was fit to Equation 3. **A)** R ($K_a^{15f}/K_a^{3\text{-nitrophenol}}$) = 0.0316 ± 0.0063; R^2 = 0.9955; **B)** R ($K_a^{15g}/K_a^{3\text{-nitrophenol}}$) = 0.0058 ± 0.0002; R^2 = 0.9999; **C)** ($K_a^{15h}/K_a^{3,4\text{-dinitrophenol}}$) = 1.213 ± 0.049; R^2 = 0.9991; **D)** ($K_a^{NButGT}/K_a^{3,4\text{-dinitrophenol}}$) = 5.474 ± 3.281; R^2 = 0.9903. Values determined using data from Tables S2 and S4.

Table S1. Summary of parameters used to determine the correlation between K_m/k_{cat} and K_i for the substrates and inhibitors with parallel structural alterations.

Compound	K_m (μM)	k_{cat}/K_m ($\mu M^{-1}min^{-1}$)	log K_m/k_{cat}	K_i (μM)	log K_i
**18a**: R=NH_2	[a]1265 ± 259	[b]1.75×10^{-3}	2.76	$4.7 \pm 3.0 \times 10^{-3}$	-2.32
**18b**: R=$NHCH_3$	[a]1825 ± 542	[b]1.36×10^{-2}	1.86	$5.1 \pm 0.5 \times 10^{-4}$	-3.30
**18c**: R=$NHCH_2CH_3$	85 ± 20	[b]3.49×10^{-3}	2.46	$2.1 \pm 0.3 \times 10^{-3}$	-2.68
**18d**: R=$NH(CH_2)_2CH_3$	120 ± 11	[b]2.55×10^{-3}	2.59	$2.0 \pm 0.2 \times 10^{-3}$	-2.69
**18e**: R=$NH(CH_2)_3CH_3$	30 ± 3	[b]3.01×10^{-5}	4.52	$3.5 \pm 0.9 \times 10^{-1}$	-0.46
**19a**: R=CH_3	---	[c]7.69×10^{-3}	2.11	---	---
**19b**: R=CH_2CH_3	---	[c]6.67×10^{-3}	2.18	---	---
**19c**: R=$(CH_2)_2CH_3$	---	[c]5.26×10^{-3}	2.28	---	---
**19d**: R=$(CH_2)_3CH_3$	---	[c]5.56×10^{-4}	3.25	---	---

[a] Values were estimated by non-linear regression of the Michealis-Menten data since saturation of hOGA with these substrates was not observed.

[b] Values of k_{cat}/K_m were determined from the linear regression of the second-order region of the Michealis-Menten plot.

[c] Values used were previously determined by Whitworth et al[77] and used for a comparison between the thiazoline and aminothiazoline series of inhibitors as transition state analogues.

Table S2. ^{13}C chemical shifts (δ, ppm) for compounds ***15c-15h*** and **NButGT** which were used to measure the parameters necessary for ΔpK_a determination.

[a]**15c**		[a]**15f**		[a]**15g**		[b]**15h**		[b]**NButGT**	
N-**C**-N	**C**-O	N-**C**-N	**C**-O	N-**C**-N	**C**-O	N-**C**-N	**C**-O	C-**C**-N	**C**-O
162.77	167.24	162.89	167.25	163.11	167.26	163.21	177.08	176.08	177.08
162.83	166.65	162.90	167.25	163.11	167.25	163.46	176.62	176.09	176.63
162.94	165.66	162.90	167.24	163.11	167.25	164.63	174.70	176.10	175.18
163.04	164.85	162.92	166.85	163.11	167.05	166.03	172.51	176.12	173.84
163.19	163.94	162.97	164.16	163.12	165.51	166.13	172.47	176.15	172.42
163.31	163.31	163.20	160.75	163.12	163.12	167.58	170.62	176.16	171.90
163.49	162.48	163.78	158.42	163.18	160.55	169.24	168.20	176.16	172.06
163.69	161.61	165.79	157.07	163.35	158.12	170.86	165.92	176.18	171.66
163.95	161.09	168.34	156.75	164.34	156.94	171.85	162.60	176.25	169.38
164.22	160.69	168.31	156.63	166.28	156.71	171.86	162.57	177.79	163.62
164.50	159.99	170.01	156.62	168.54	156.64	171.86	162.57	177.11	162.76
164.90	159.36			171.02	156.61	171.86	162.57	177.65	162.50
165.02	159.23			171.26	156.61			177.65	162.50
165.50	158.82								
166.19	158.12								
166.76	157.76								
167.20	157.48								
167.74	157.22								
168.34	157.10								
168.93	156.77								

[a] The chemical shifts which were directly compared were those of the amino-thiazoline carbon (N-**C**-N) for inhibitors ***15c-15g*** and those of the **C**-O carbon of 3-nitrophenol. ***15c*:** 0.161 mmol of each compound titrated with 1M HCl in 10 μL increments (20 injections); ***15f*:** 0.0262 mmol (11 injections), ***15g:*** 0.0302 mmol (13 injections).

[b] The chemical shifts which were directly compared were those of the amino-thiazoline carbon (N-**C**-N) for inhibitors ***15h*** and **NButGT** and those of the **C**-O carbon of 3,4-dinitrophenol. ***15h:*** 0.0397 mmol (12 injections) of each compound, titrated with 0.5 M HCl in 10 μL increments. **NButGT:** 0.0728 mmol of each compound titrated with 0.5 M HCl in 10 μL increments up to 80 μL, then 20 μL increments after that, up to 140 μL (13 injections).

Table S3. Data collections statistics for X-ray structures of **11a**, **11b** and **15e** with **BtGH84**.

	**11a**	**11b**	**15e**
**pdb-ID**	5fky	5fl1	5fl0
x-ray-source	ESRF-ID14-1	Diamond-I04	Diamond-I04
wavelength	0.93400	0.97949	0.97625
resolution range	33.8-1.80	49.08-1.95	44.96-1.95
space group	P1	$P22_12_1$	$P22_12_1$
unit cell Å	a=51.4 Å<br>b=93.7 Å<br>c=99.0 Å<br>α=104.07<br>β=94.18<br>γ=102.97°	a=51.5 Å<br>b=162.1 Å<br>c=223.2 Å<br>α=β=γ=90°	a=51.5 Å<br>b=162.1 Å<br>C=223.6 Å<br>α=β=γ=90°
completeness [%]	95.8(91.9)	99.8(96.9)	99.9(100)
R_{merge}	0.080(0.424)	0.129(1.724)	0.097(1.860)
R_{meas}	0.113(0.599)	0.135(1.801)	0.105(2.013)
R_{pim}	0.080(0.424)	0.037(0.512)	0.040(0.762)
I/sig(I)	9.4(1.9)	15.7(1.8)	12.2(1.2)
multiplicity	2.0(2.0)	13.2(12.0)	6.7(6.9)
Wilson B-Factor [Å²]	15.6	30.3	35.0
solvent content [%]	54.8	55.3	56.7
**Refinement**			
R_{work}/R_{free} [%]	21.1/24.9	19.3/22.2	19.9/22.7
average B-Factor			
protein	25.59	48.8	52.35
water	28.43	41.3	45.18
ligand	10.2	29.5	38.80
r.m.s.d.			
bond length [Å]	0.017	0.008	0.007
bond angle [°]	1.698	0.897	0.835
Ramachandran plot			
favored/allowed/ disallowed [%]	96.8/3.2/0.0	95.6/4.3/0.1	96.9/3.0/0.1

Values in parentheses refer to the highest resolution shell

Table S4. R, ΔpK_a, absolute pK_a, and fitting parameters determined from each titration for compounds **15c**, **15f**, and **15g** (3-Nitrophenol as the reference compound) and **15h** and NButGT (3,4-dinitrophenol as a reference compound).

Inhibitor	[a]Δ_d (ppm)	[b]Δ^r (ppm)	[c]Δ^x (ppm)	R (K_a^x/K_a^r)	[d]Δ pK_a	[e] pK_a
**15c**	4.47	10.47	-6.16	0.1823 ± 0.0042	-0.74	7.68
**15f**	4.36	10.63	-7.12	0.0316 ± 0.0063	-1.50	6.92
**15g**	4.15	10.65	-8.15	0.0058 ± 0.0002	-2.24	6.18
**15h**	13.87	14.51	-8.65	1.213 ± 0.049	0.08	5.33
**NButGT**	1.00	14.58	-1.57	5.47 ± 3.28	0.73	4.65

[a] The difference between the ^{13}C chemical shift of the deprotonated inhibitor (2'-aminothiazoline carbon) and 3-nitrophenol (C-O carbon) for **15c-15g** and 3,4-dinitrophenol for 15h and NButGT (Δd = δdx-δdr); Determined for each point during the course of the titration.

[b] The chemical shift difference between the de-protonated and protonated 3-nitrophenol C-O (15c-15g) and for that of 3,4-dinitrophenol (15h and NButGT) (Δr = δdr- δpr).

[c] The chemical shift difference between the de-protonated and protonated thiazoline for each inhibitor (Δx = δdx - δpx).

[d] Δ p*K*a = log R. [e] p*K*a = 8.42 - Δ p*K*a for 15c-15g and 5.42 - Δp*K*a for 15h and NButGT.

^{1}H and ^{13}C spectra for 2'-aminothiazoline inhibitors and urea substrates:

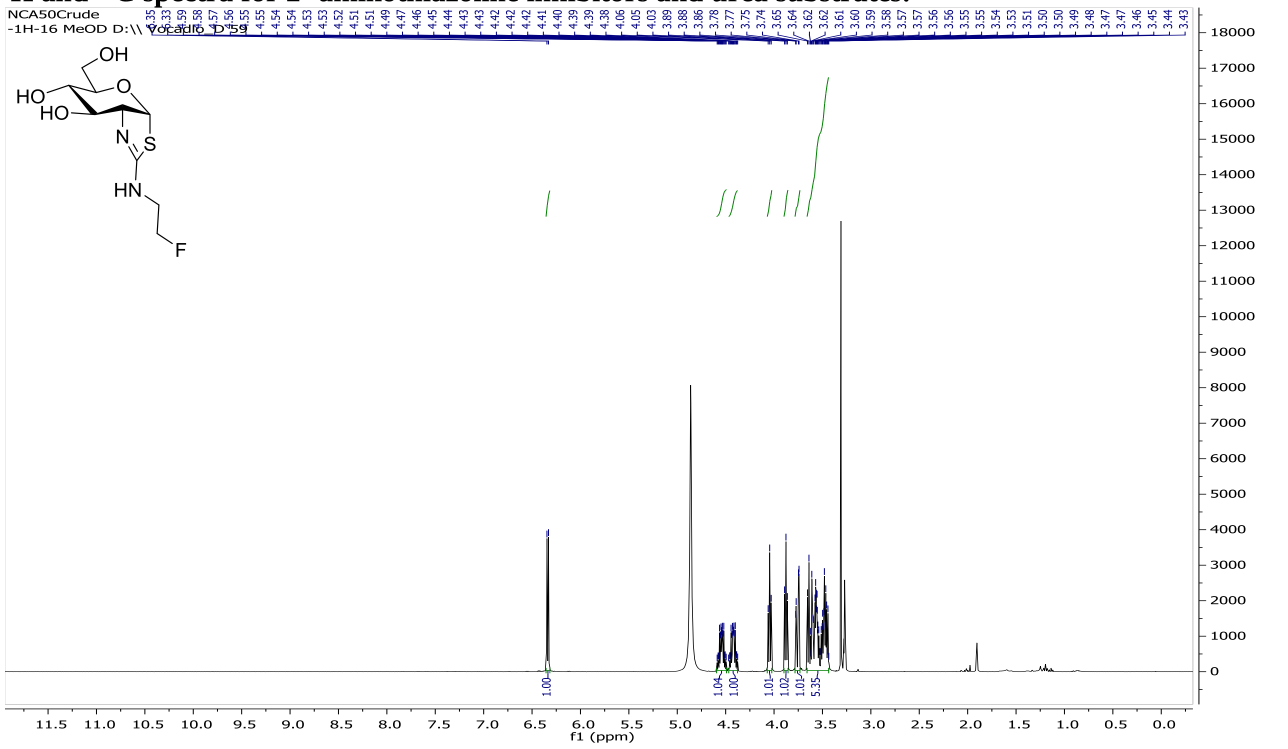

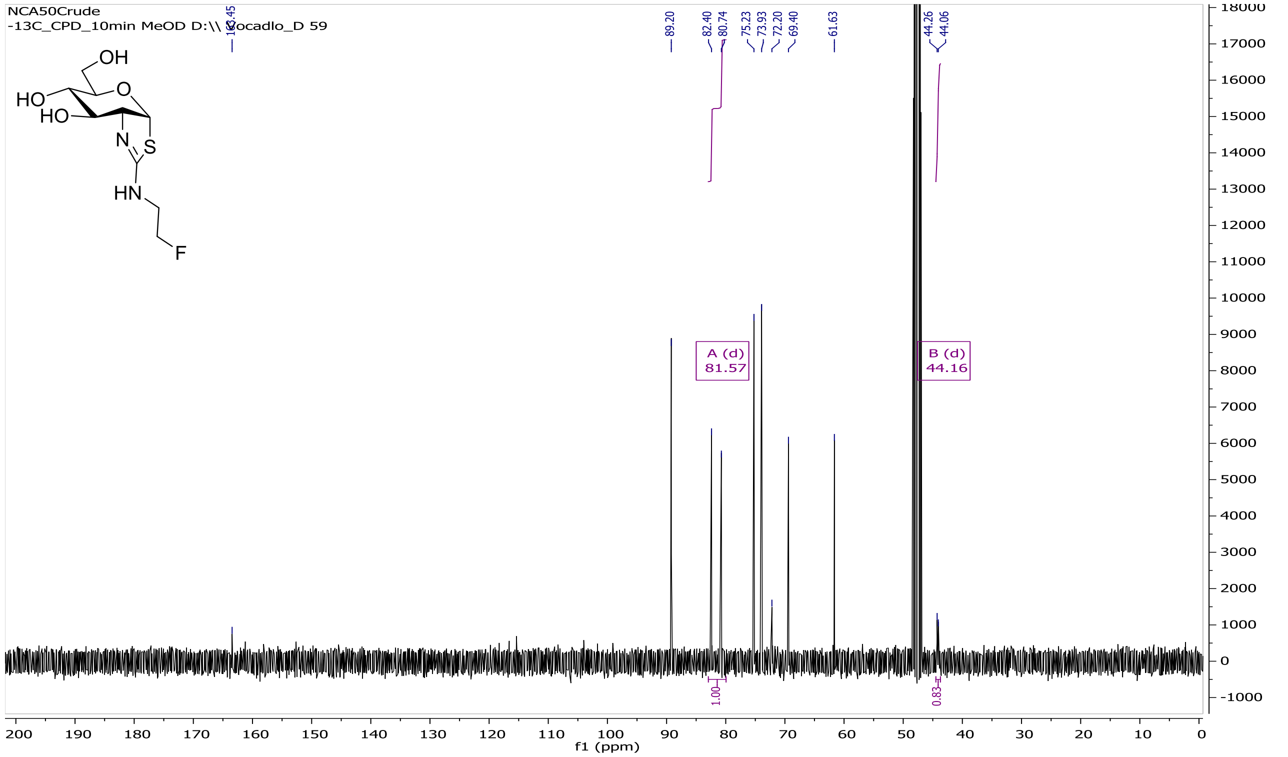

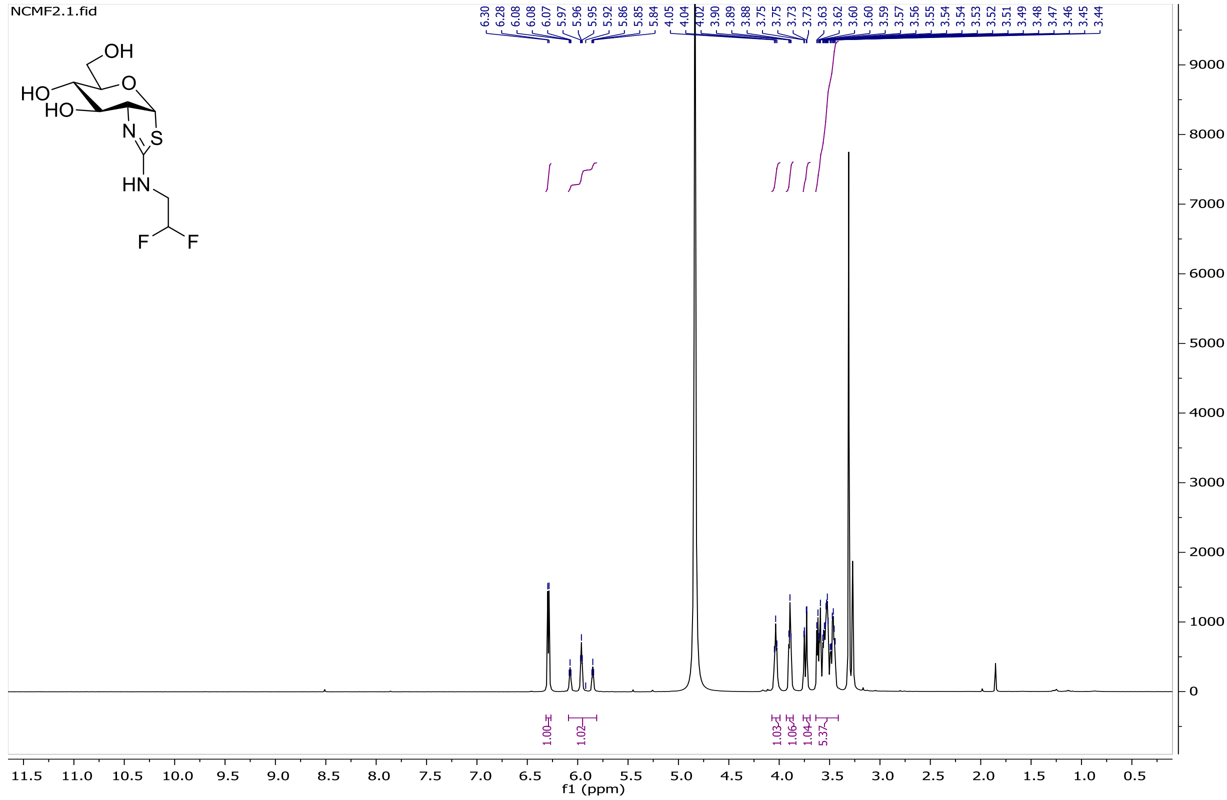

NCMF2.1.fid
f1 (ppm)

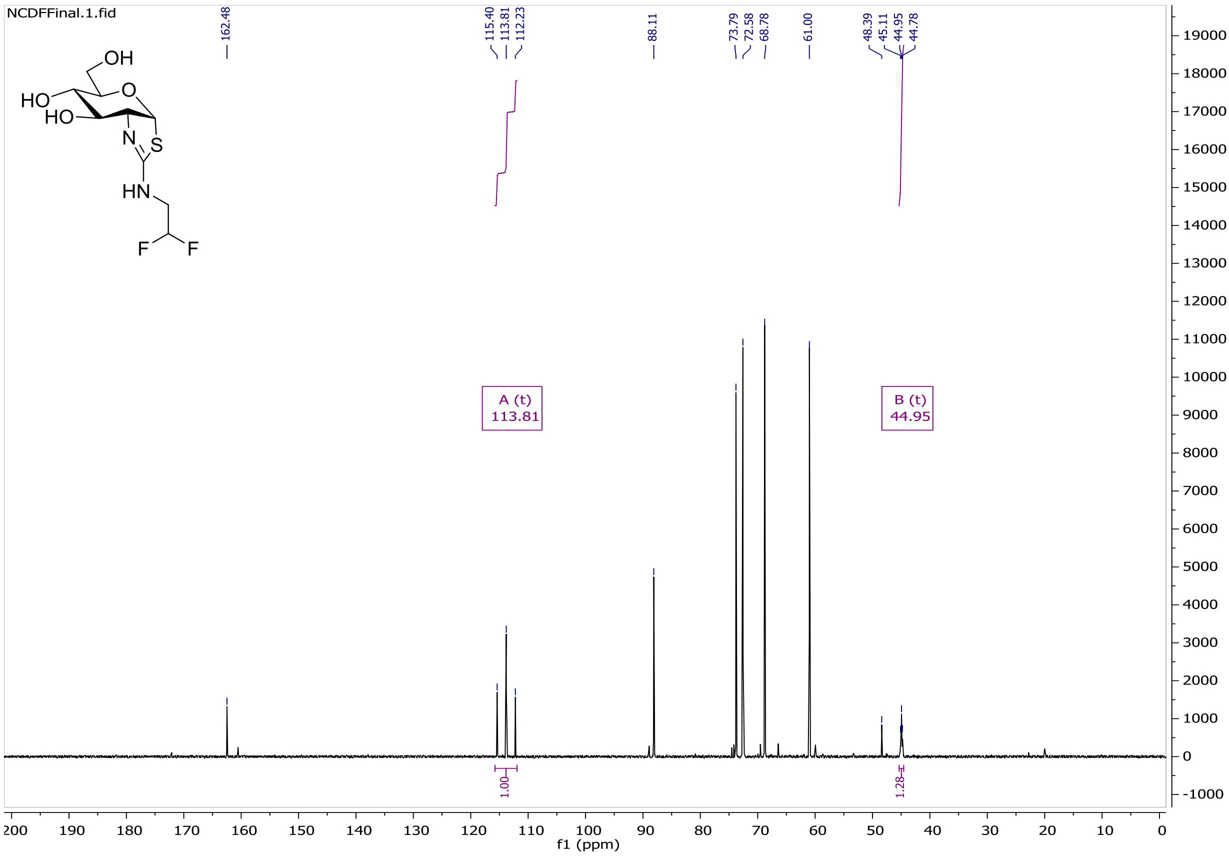

NCDFFinal.1.fid
A (t)
113.81
B (t)
44.95
f1 (ppm)

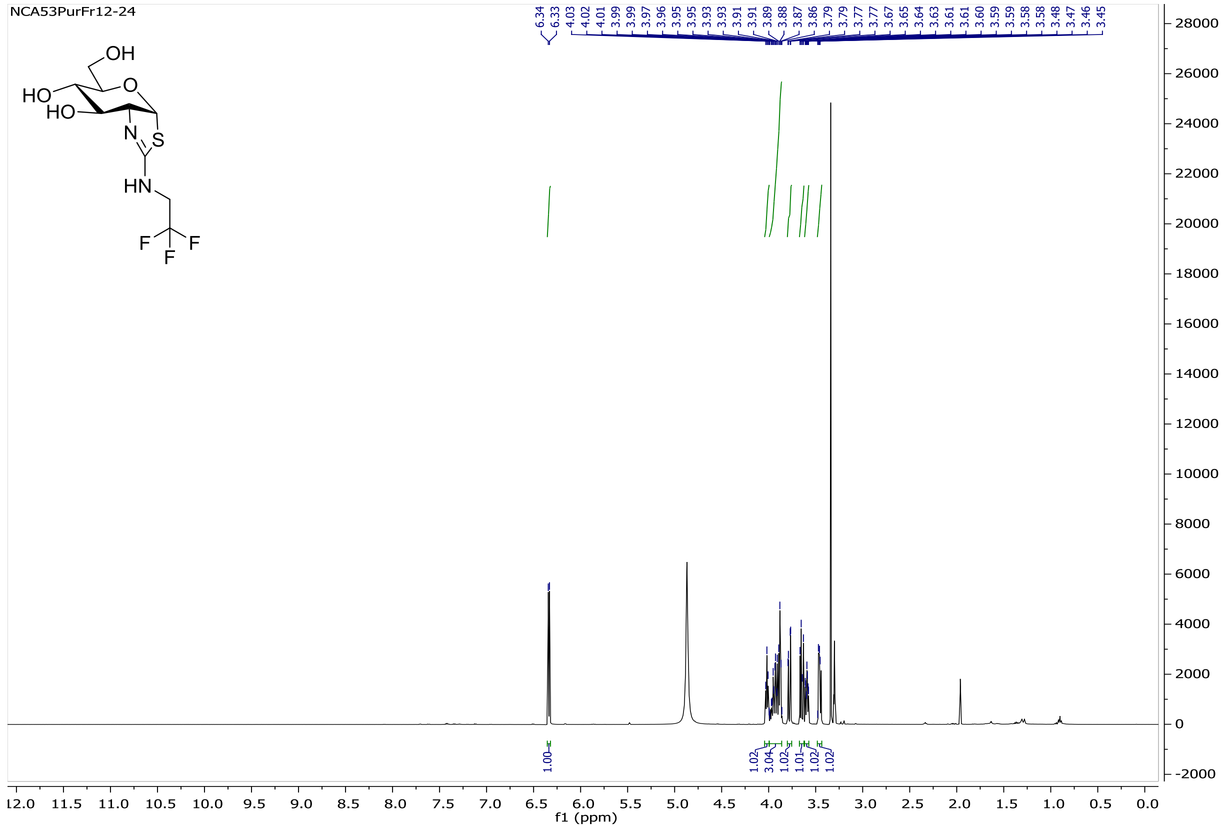

NCA53PurFr12-24
f1 (ppm)

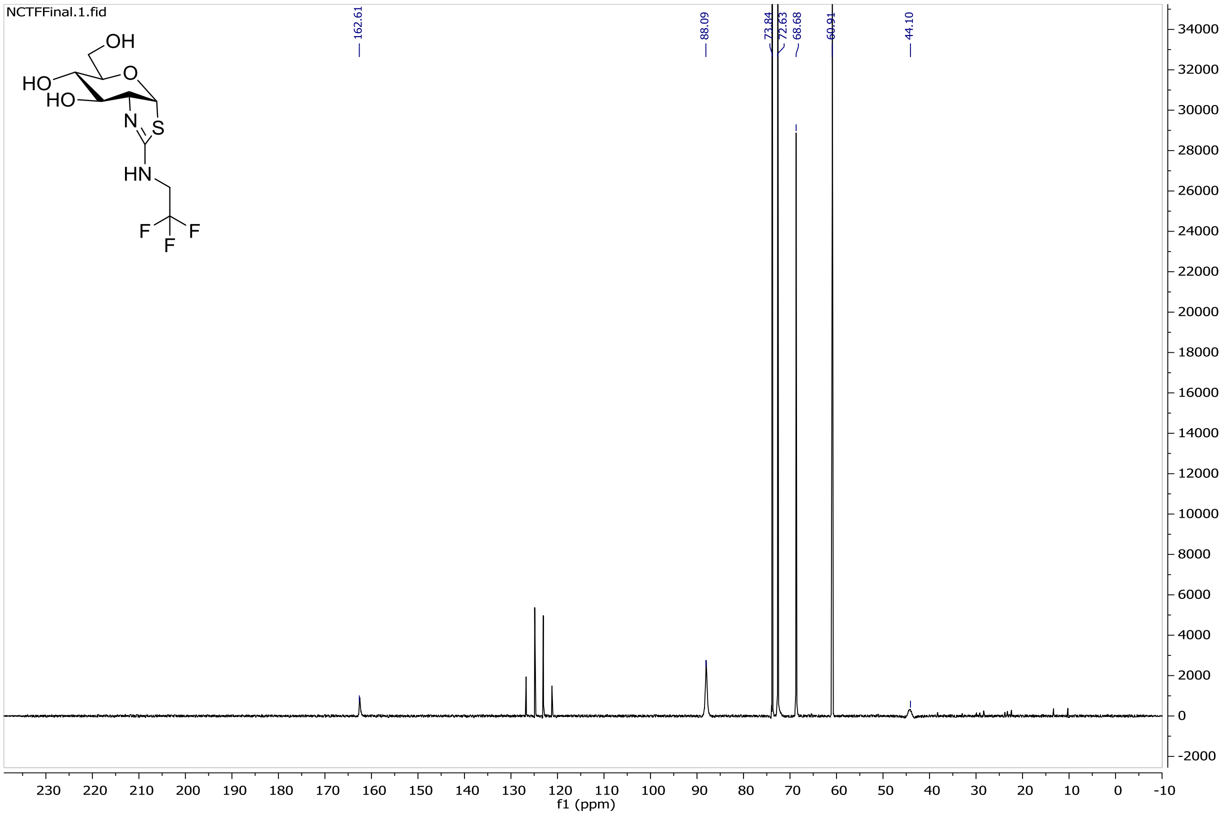

NCTFFinal.1.fid
162.61
88.09
73.84
72.63
68.68
60.91
44.10
f1 (ppm)

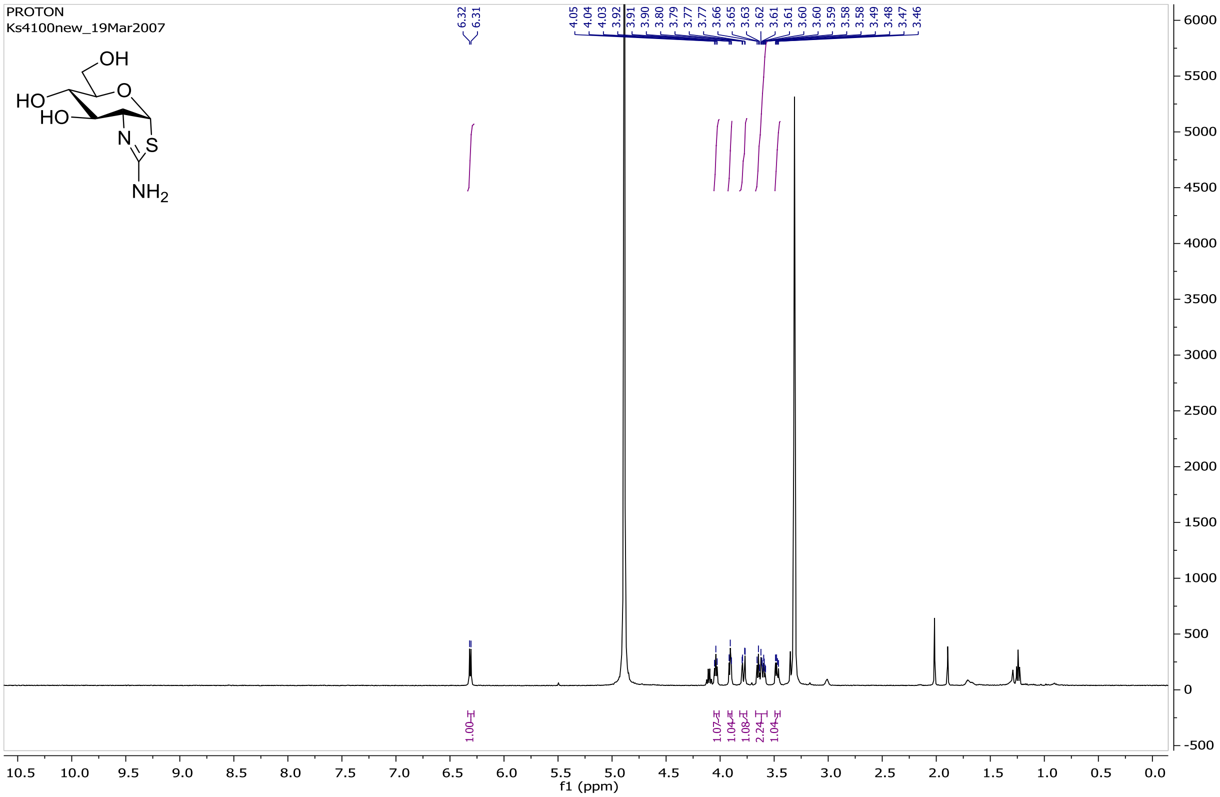
PROTON
Ks4100new_19Mar2007
OH
HO
HO
O
N
S
NH2
6.32
6.31
4.05
4.04
4.03
3.92
3.91
3.90
3.80
3.79
3.77
3.77
3.66
3.65
3.63
3.62
3.61
3.61
3.60
3.60
3.59
3.58
3.58
3.49
3.48
3.47
3.46
1.00
1.07
1.04
1.08
2.24
1.04
f1 (ppm)

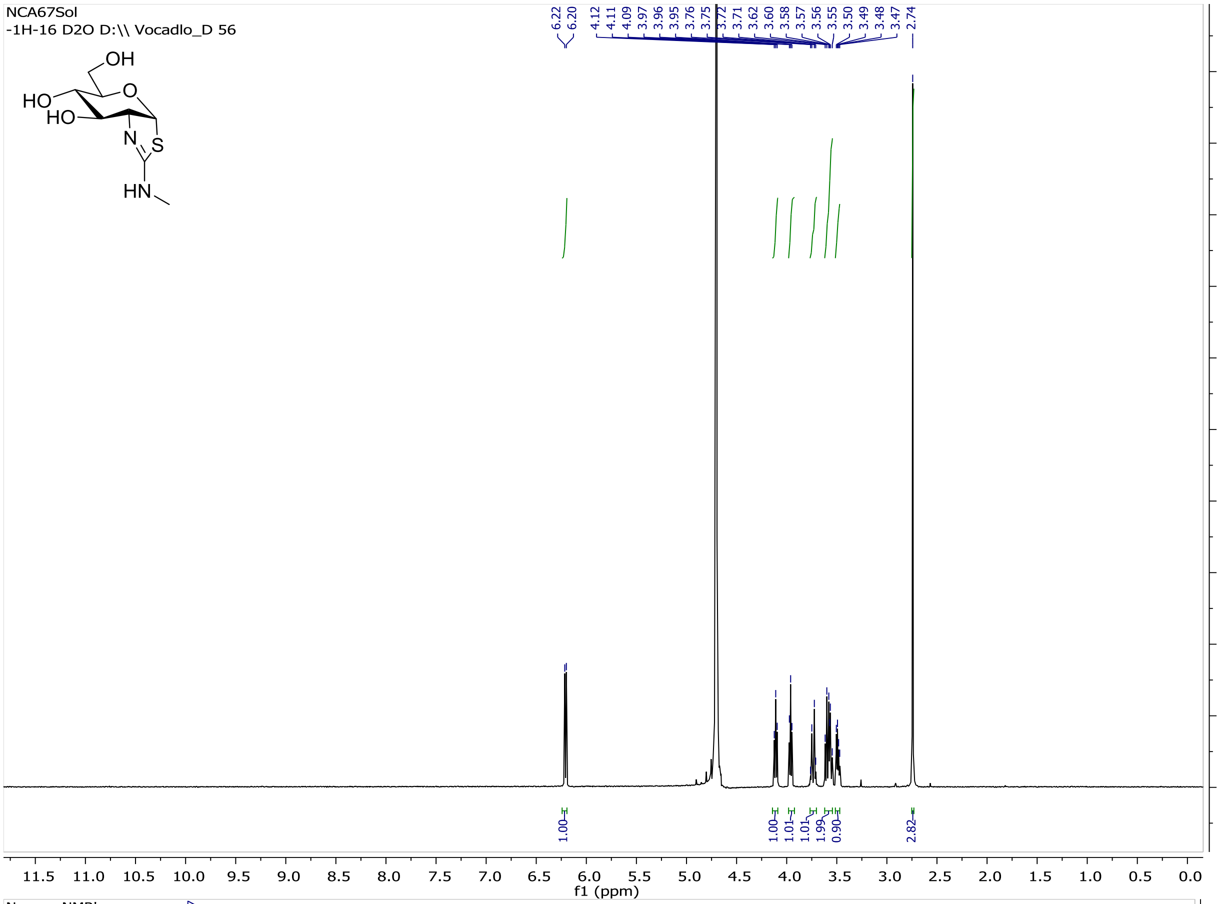
NCA67Sol
-1H-16 D2O D:\\ Vocadlo_D 56
OH
HO
HO
O
N
S
HN
6.22
6.20
4.12
4.11
4.09
3.97
3.96
3.95
3.76
3.75
3.72
3.71
3.62
3.60
3.58
3.57
3.56
3.55
3.50
3.49
3.48
3.47
2.74
1.00
1.00
1.01
1.01
1.99
0.90
2.82
f1 (ppm)

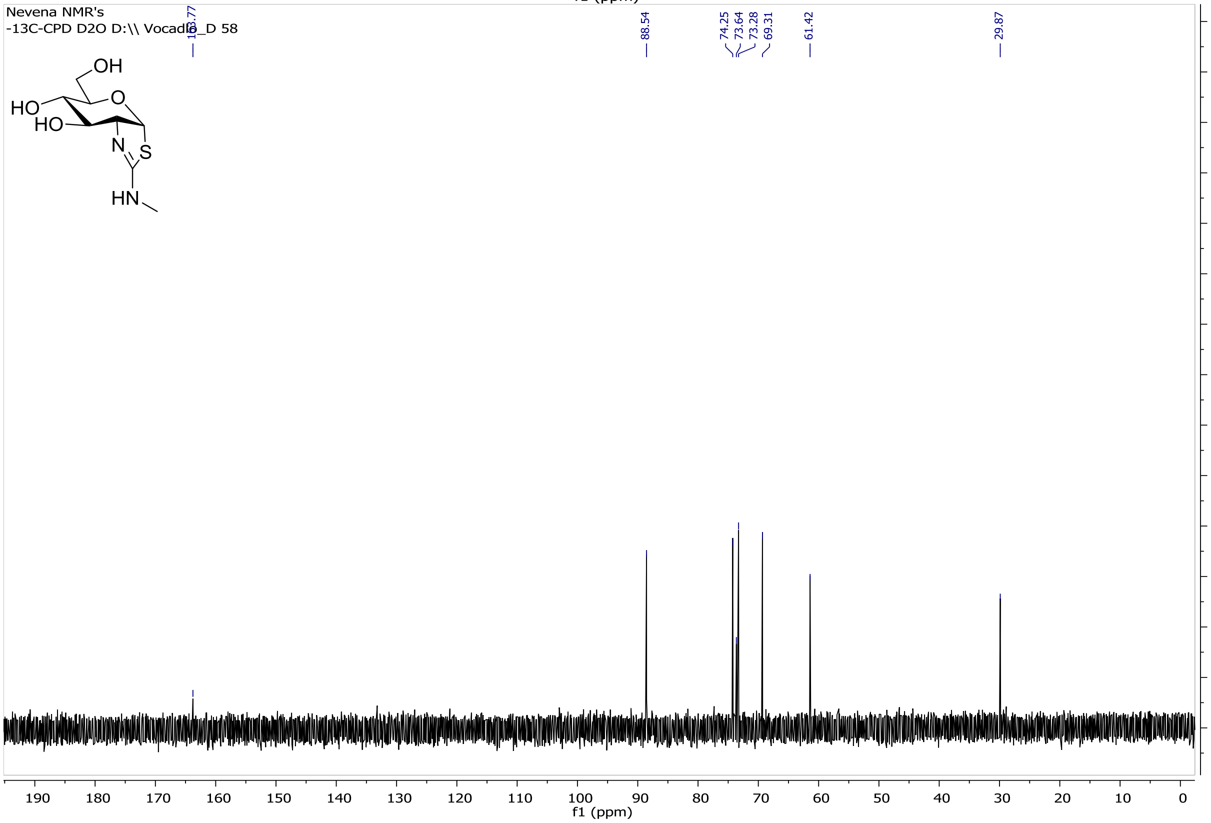
Nevena NMR's
-13C-CPD D2O D:\\ Vocadlo_D 58
88.54
74.25
73.64
73.28
69.31
61.42
29.87
OH
HO
HO
O
N
S
HN
f1 (ppm)

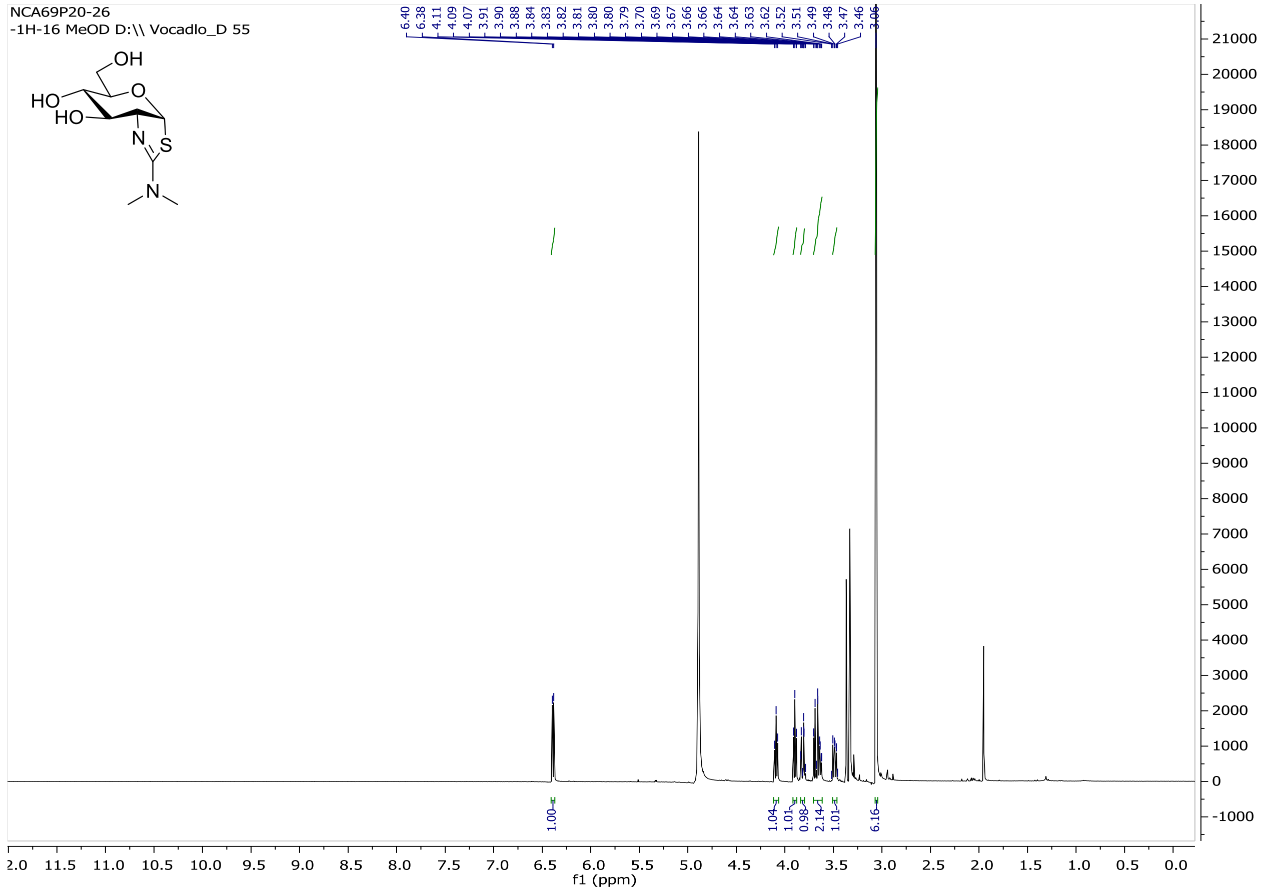
NCA69P20-26
-1H-16 MeOD D:\\ Vocadlo_D 55
6.40
6.38
4.11
4.09
4.07
3.91
3.90
3.88
3.84
3.83
3.82
3.81
3.80
3.80
3.79
3.70
3.69
3.67
3.66
3.66
3.64
3.64
3.63
3.62
3.52
3.51
3.49
3.48
3.47
3.46
3.06
OH
HO
HO
O
N
S
N
1.00
1.04
1.01
0.98
2.14
1.01
6.16
f1 (ppm)

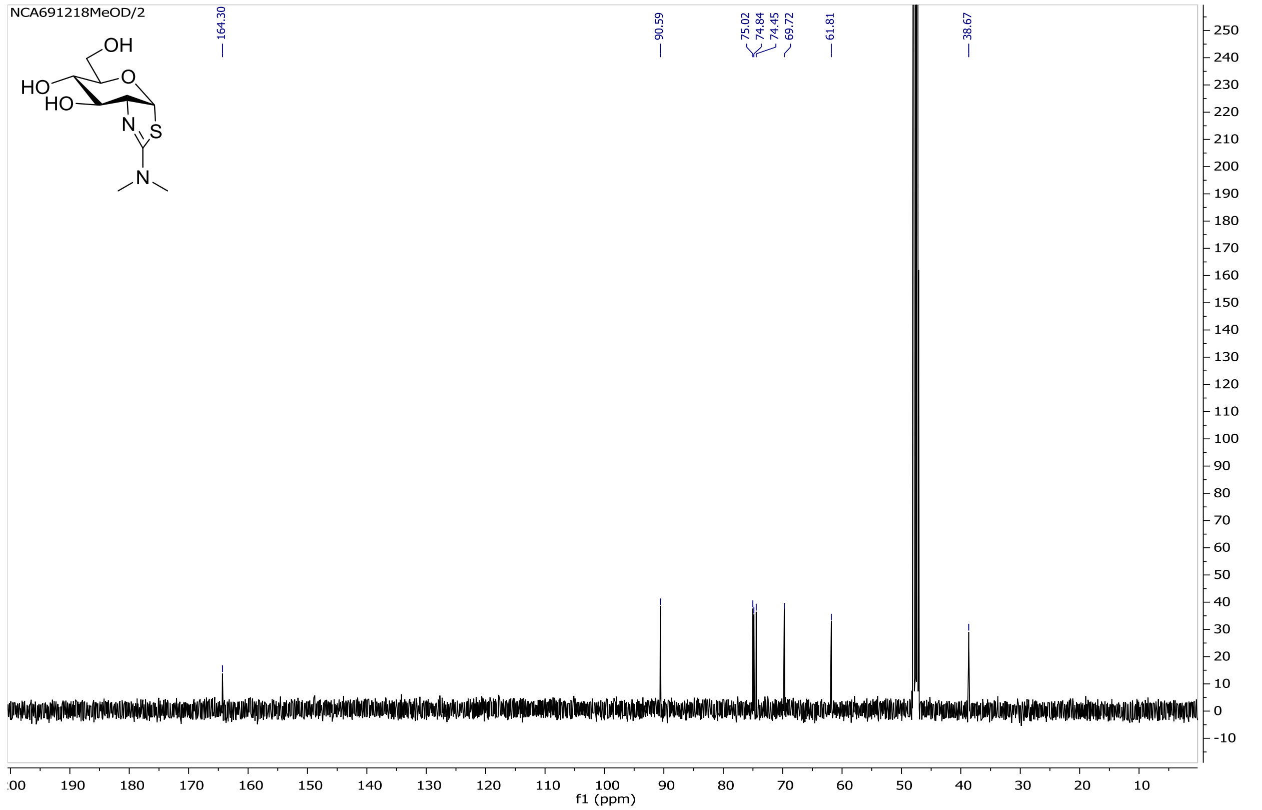
NCA691218MeOD/2
164.30
90.59
75.02
74.84
74.45
69.72
61.81
38.67
OH
HO
HO
O
N
S
N
f1 (ppm)

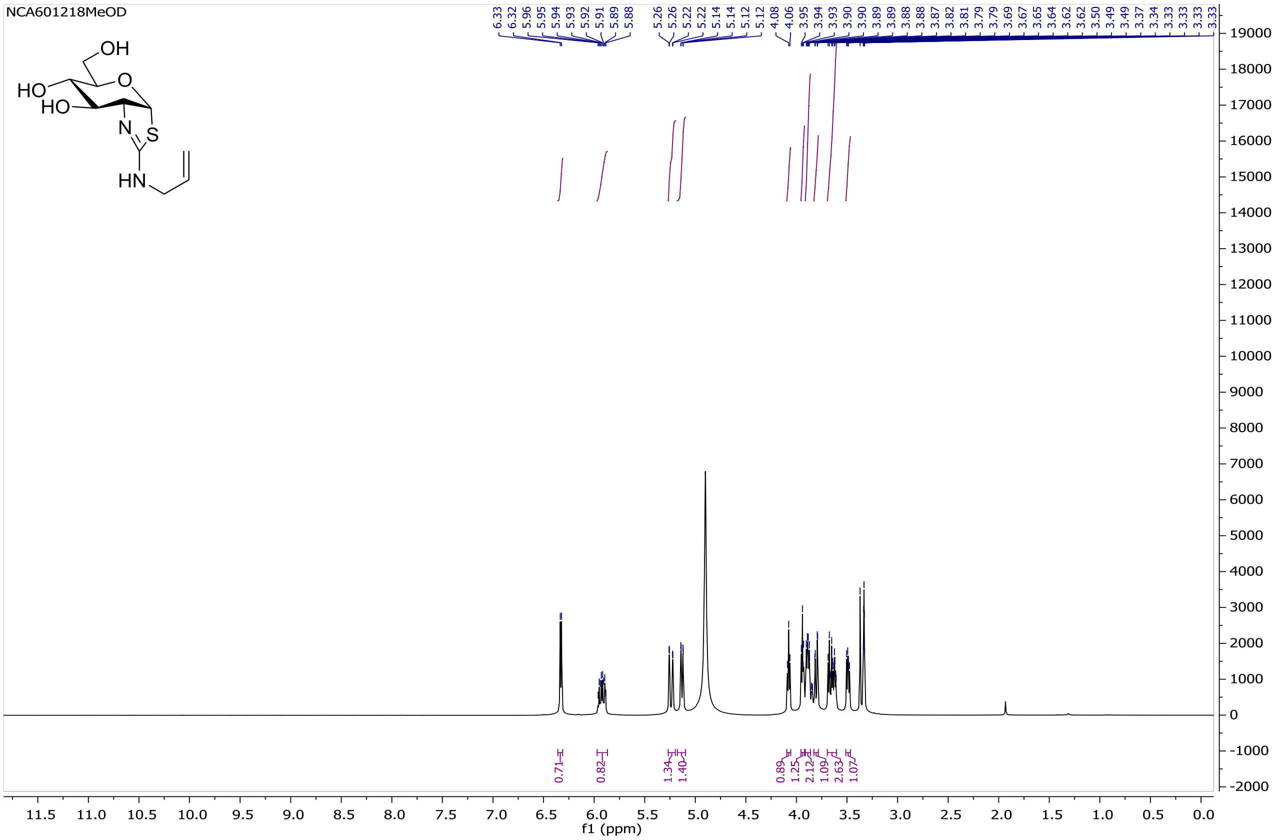
NCA601218MeOD
OH
HO
O
HO
N
S
HN
f1 (ppm)

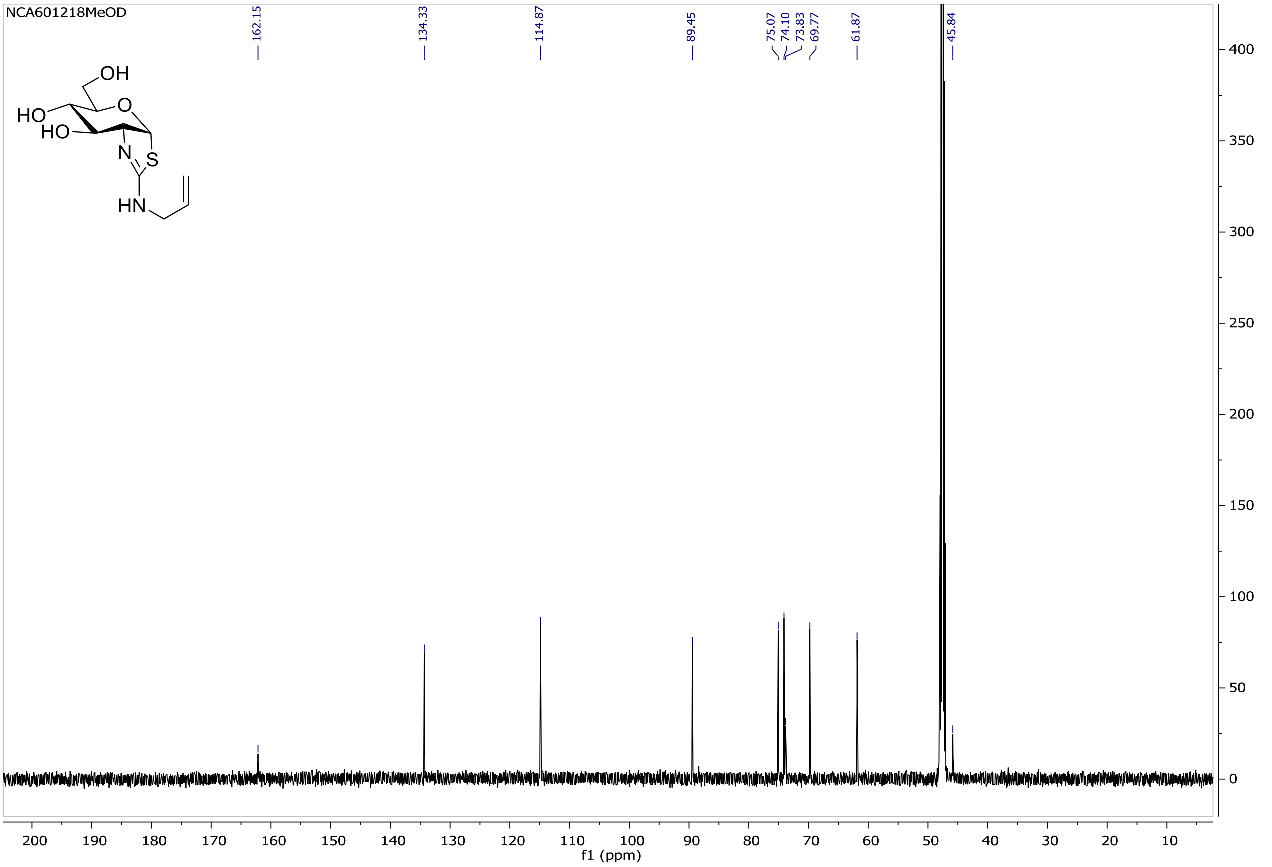
NCA601218MeOD
162.15
134.33
114.87
89.45
75.07
74.10
73.83
69.77
61.87
45.84
OH
HO
O
HO
N
S
HN
f1 (ppm)

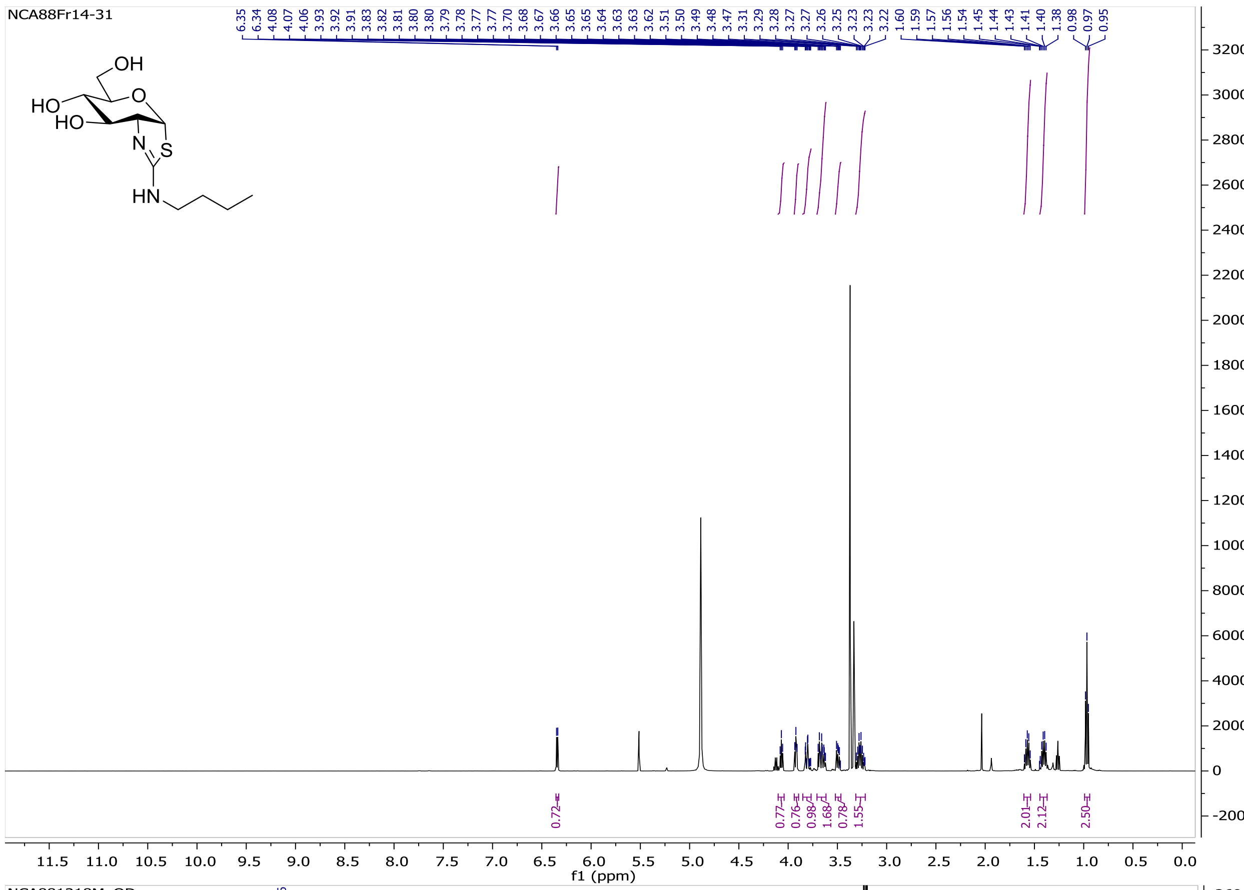
NCA88Fr14-31
6.35 6.34 4.08 4.07 4.06 3.93 3.92 3.91 3.83 3.82 3.81 3.80 3.79 3.78 3.77 3.70 3.68 3.67 3.66 3.65 3.65 3.64 3.63 3.63 3.62 3.51 3.50 3.49 3.48 3.47 3.31 3.29 3.28 3.27 3.27 3.26 3.25 3.23 3.23 3.22 1.60 1.59 1.57 1.56 1.54 1.45 1.44 1.43 1.41 1.40 1.38 0.98 0.97 0.95
OH
HO
HO
O
N
S
HN
0.72
0.77 0.76 0.98 1.68 0.78 1.55
2.01 2.12
2.50
f1 (ppm)

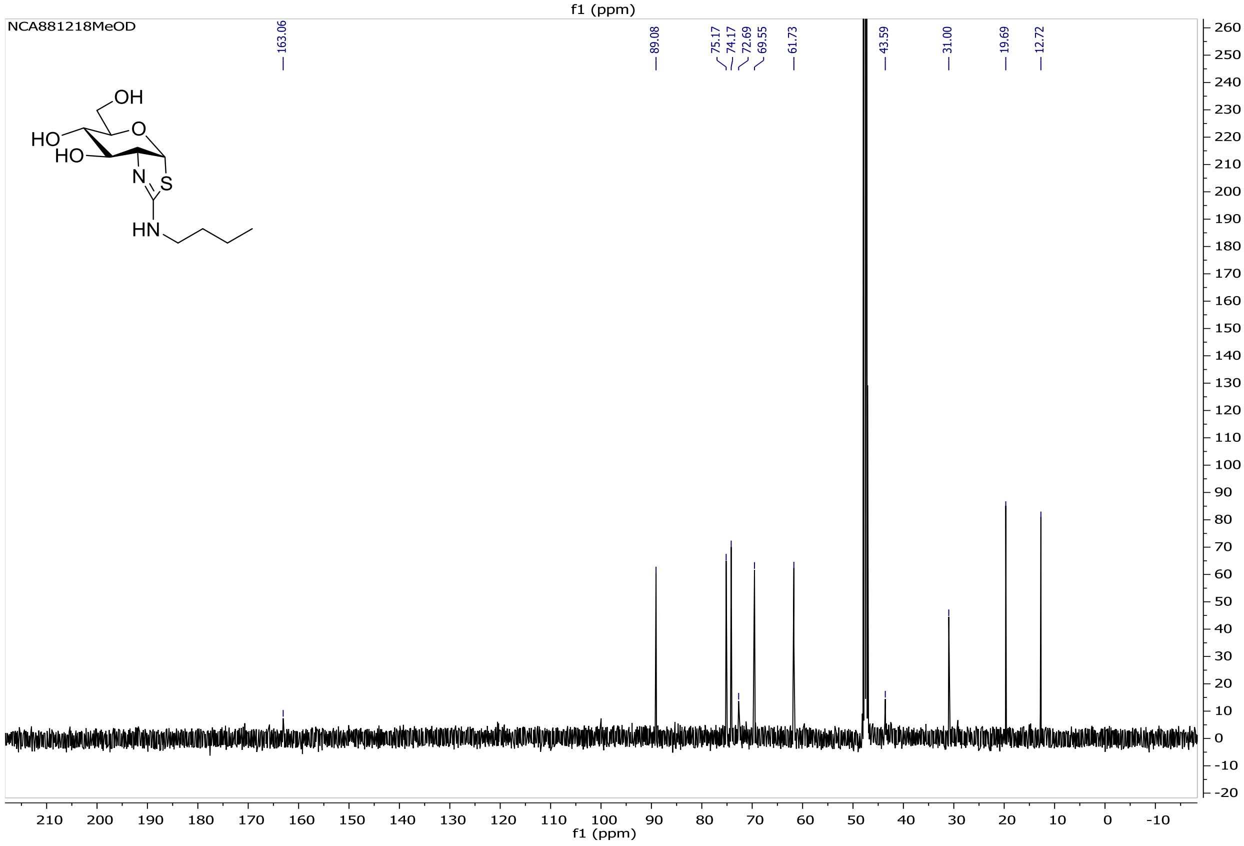
NCA881218MeOD
163.06
89.08
75.17 74.17 72.69 69.55
61.73
43.59
31.00
19.69
12.72
OH
HO
HO
O
N
S
HN
f1 (ppm)

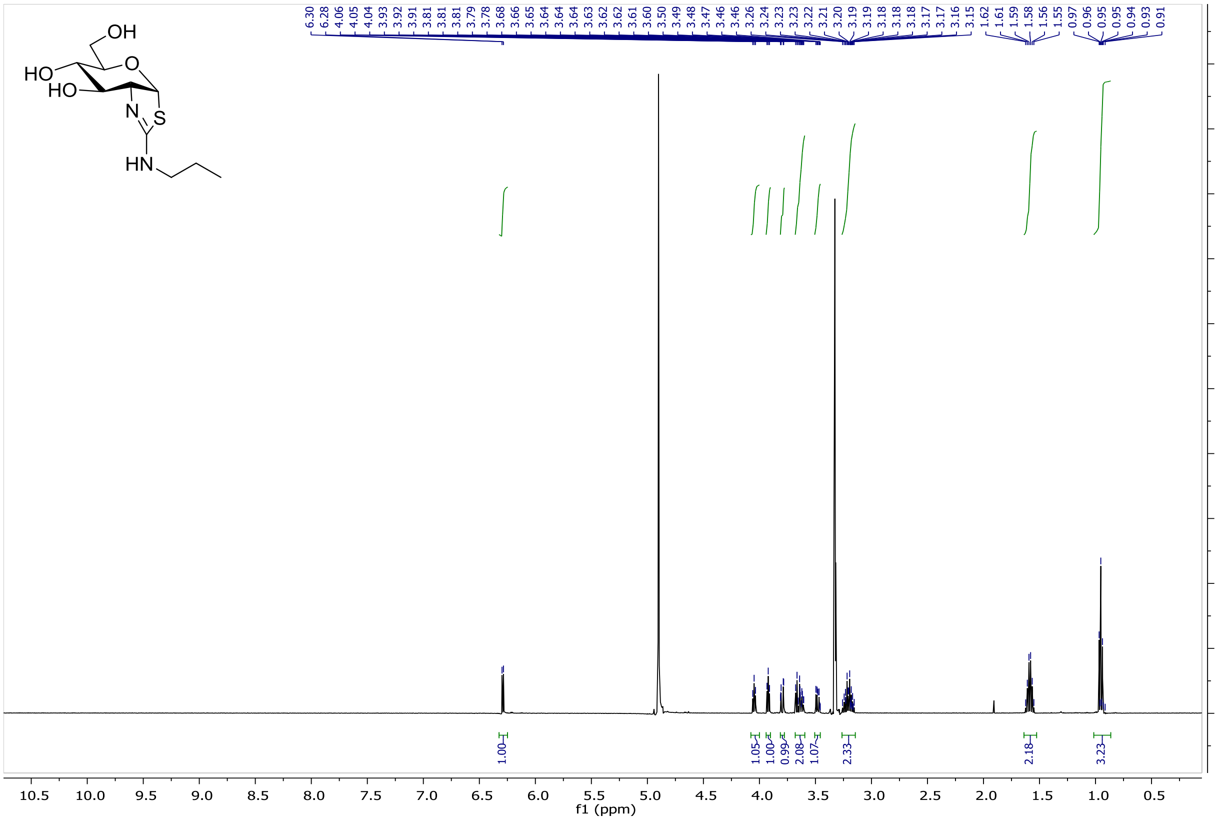

OH
HO
HO
O
N
S
HN
6.30 6.28 4.06 4.05 4.04 3.93 3.92 3.91 3.81 3.81 3.81 3.79 3.78 3.68 3.66 3.65 3.64 3.64 3.64 3.63 3.62 3.62 3.61 3.60 3.50 3.49 3.48 3.47 3.46 3.26 3.24 3.23 3.23 3.22 3.21 3.20 3.19 3.19 3.18 3.18 3.18 3.17 3.17 3.16 3.15 1.62 1.61 1.59 1.58 1.56 1.55 0.97 0.96 0.95 0.95 0.94 0.93 0.91
1.00
1.05
1.00
0.99
2.08
1.07
2.33
2.18
3.23
f1 (ppm)

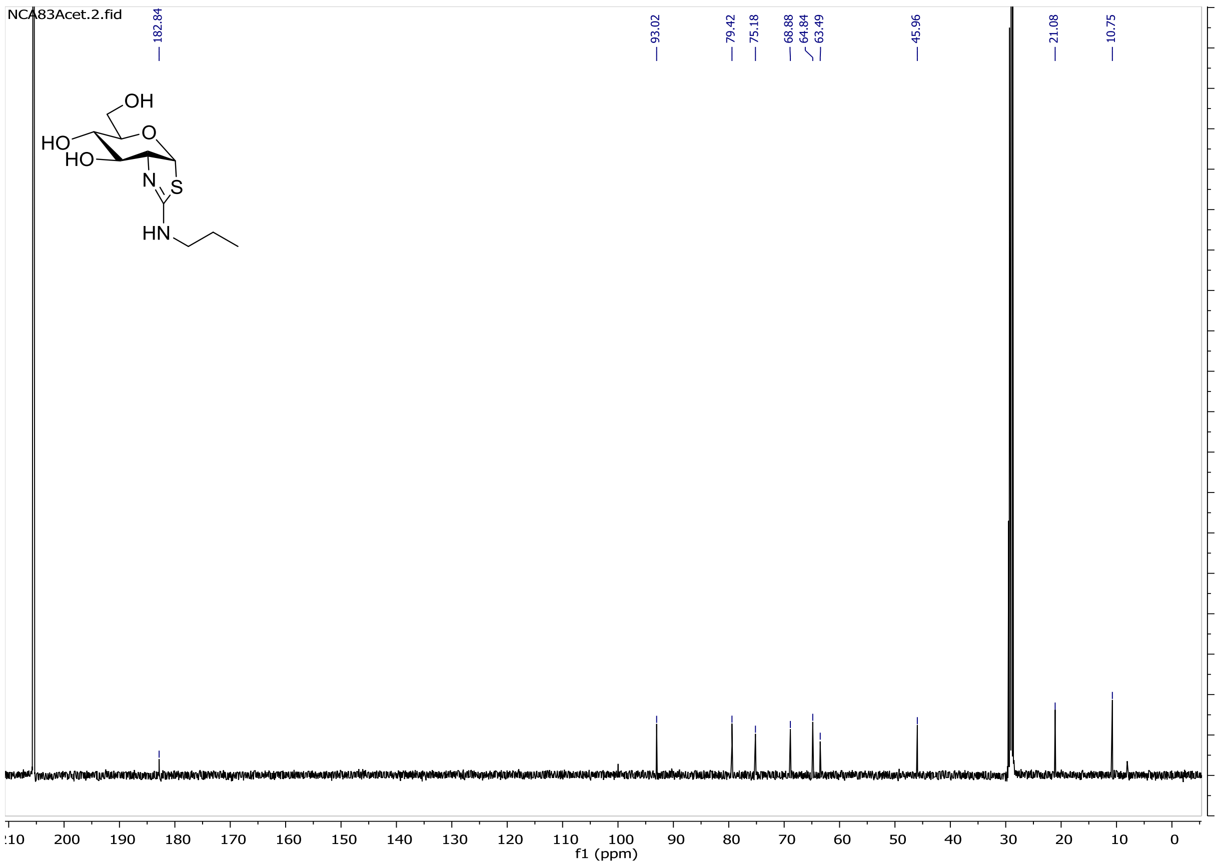

NCA83Acet.2.fid
182.84
93.02
79.42
75.18
68.88
64.84
63.49
45.96
21.08
10.75
OH
HO
HO
O
N
S
HN
f1 (ppm)

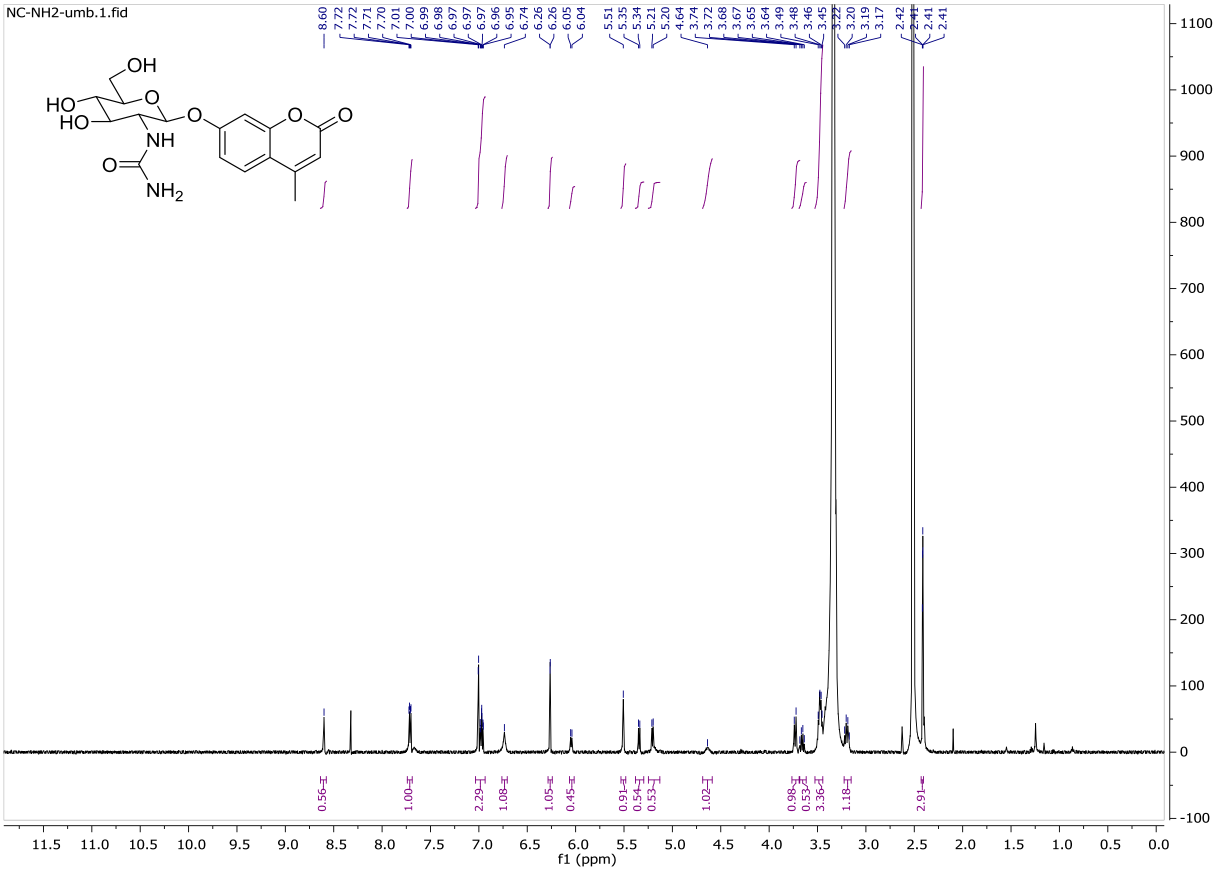
NC-NH2-umb.1.fid
8.60 7.72 7.72 7.71 7.70 7.01 7.00 6.99 6.98 6.97 6.97 6.97 6.96 6.95 6.74 6.26 6.26 6.05 6.04 5.51 5.35 5.34 5.21 5.20 4.64 3.74 3.72 3.68 3.67 3.65 3.64 3.49 3.48 3.46 3.45 3.22 3.20 3.19 3.17 2.42 2.41 2.41 2.41
0.56 1.00 2.29 1.08 1.05 0.45 0.91 0.54 0.53 1.02 0.98 0.53 3.36 1.18 2.91
f1 (ppm)
HO
HO
OH
O
O
NH
O
NH2
O
O

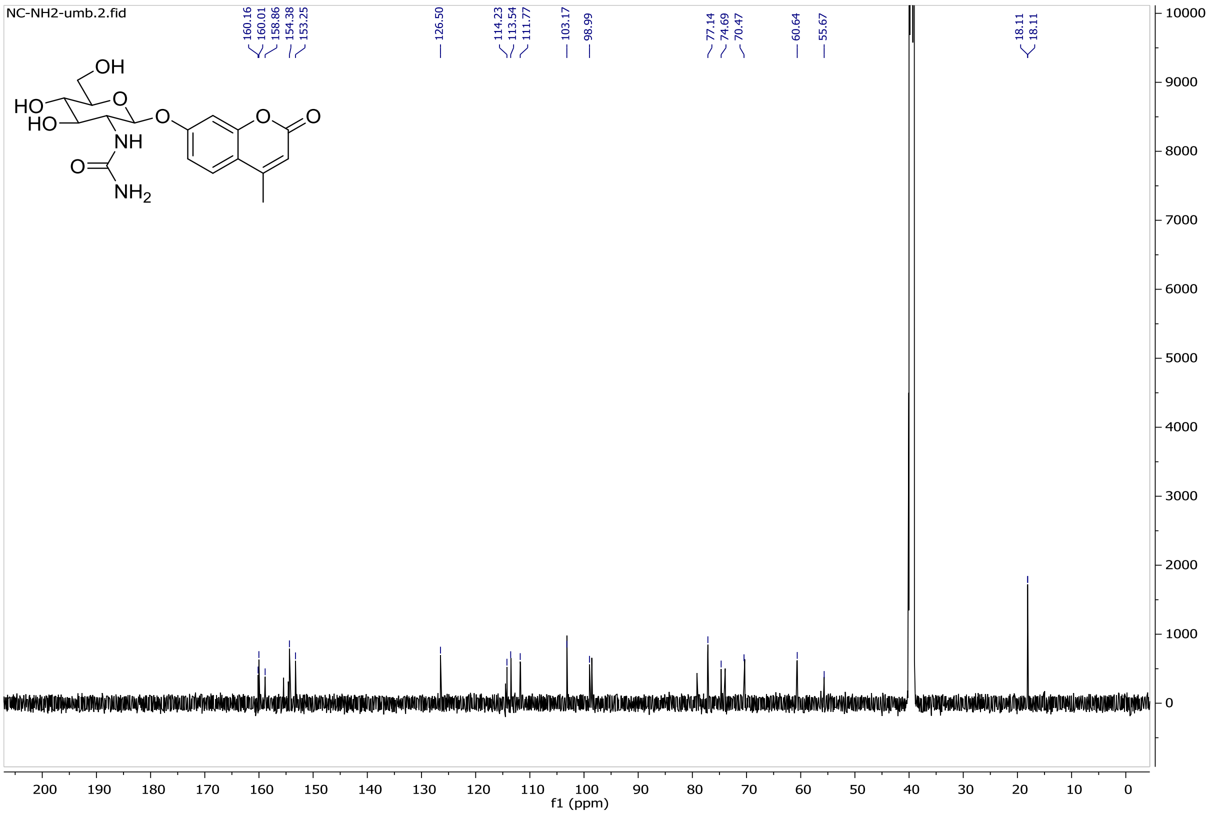
NC-NH2-umb.2.fid
160.16 160.01 158.86 154.38 153.25 126.50 114.23 113.54 111.77 103.17 98.99 77.14 74.69 70.47 60.64 55.67 18.11 18.11
f1 (ppm)
HO
HO
OH
O
O
NH
O
NH2
O
O

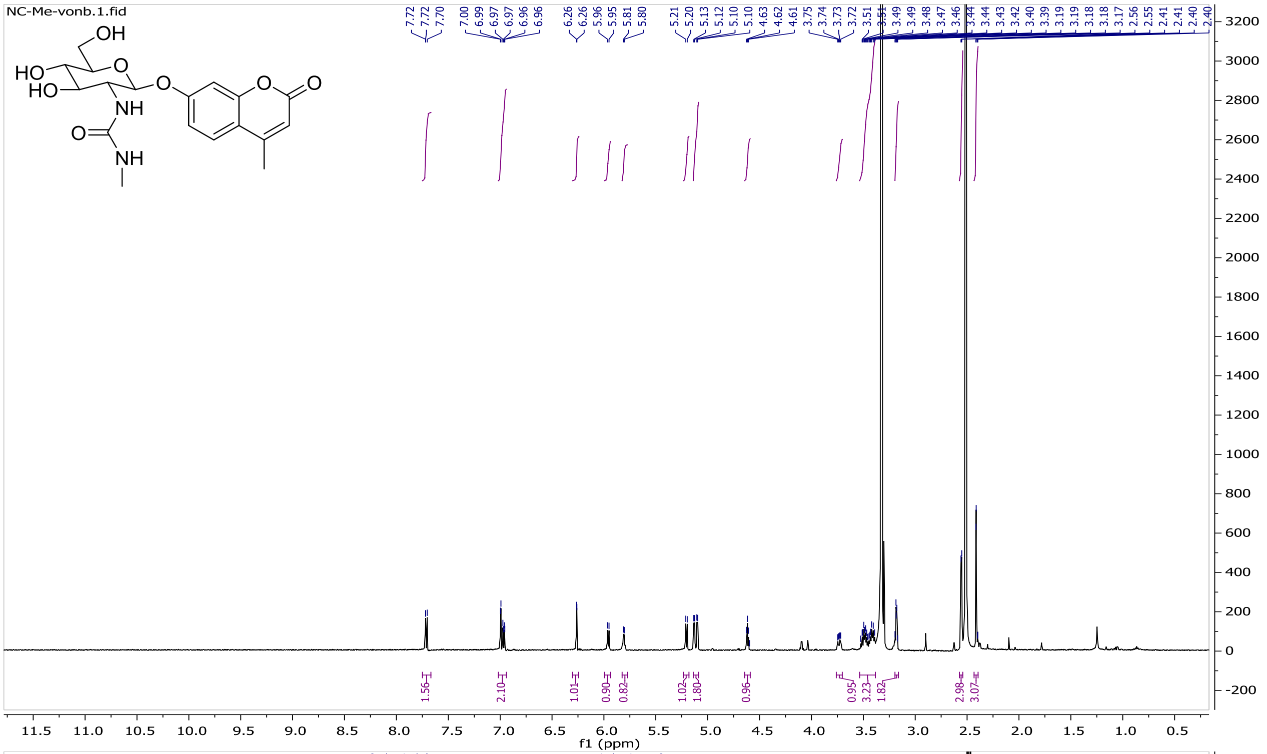

NC-Me-vonb.1.fid
f1 (ppm)

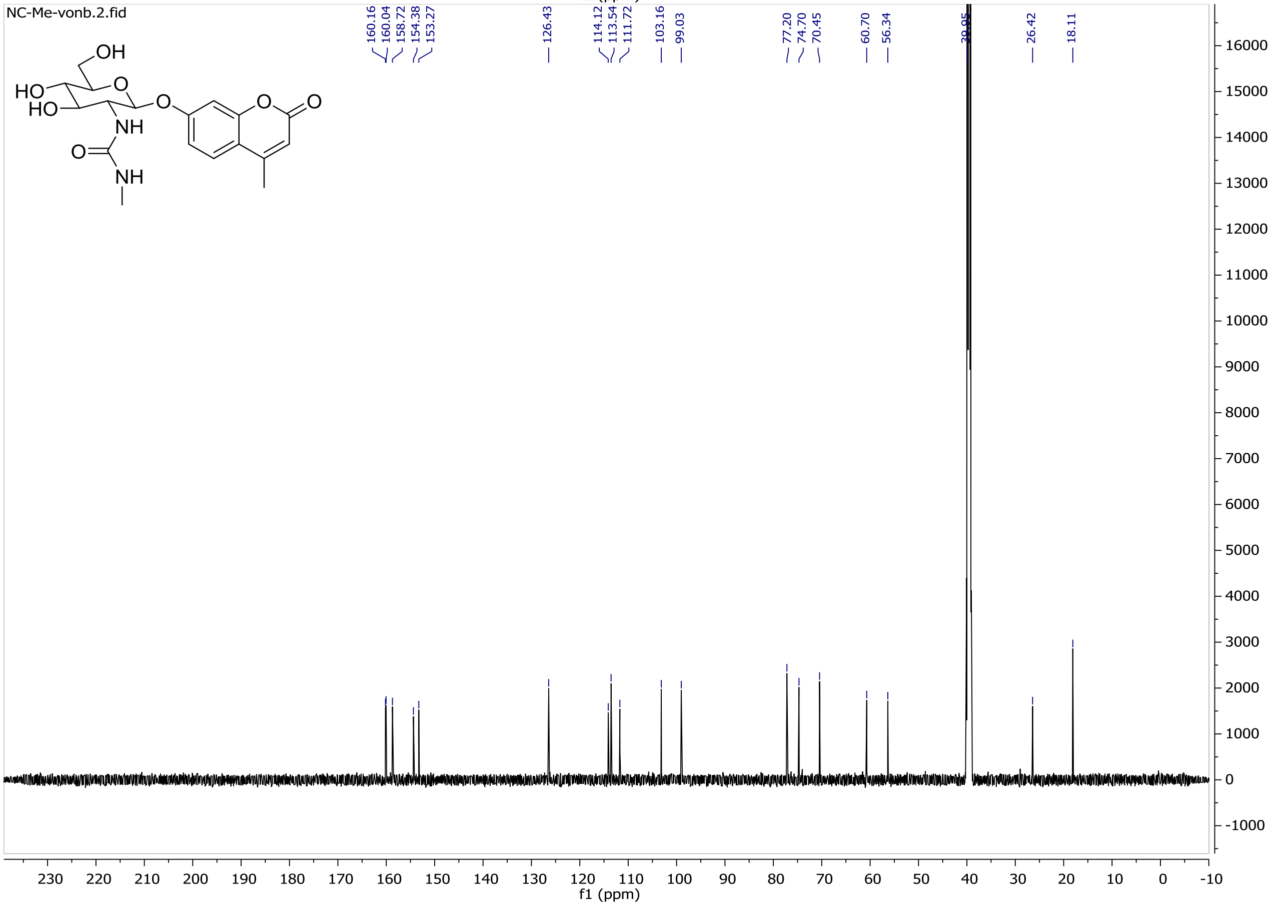

NC-Me-vonb.2.fid
160.16
160.04
158.72
154.38
153.27
126.43
114.12
113.54
111.72
103.16
99.03
77.20
74.70
70.45
60.70
56.34
26.42
18.11
f1 (ppm)

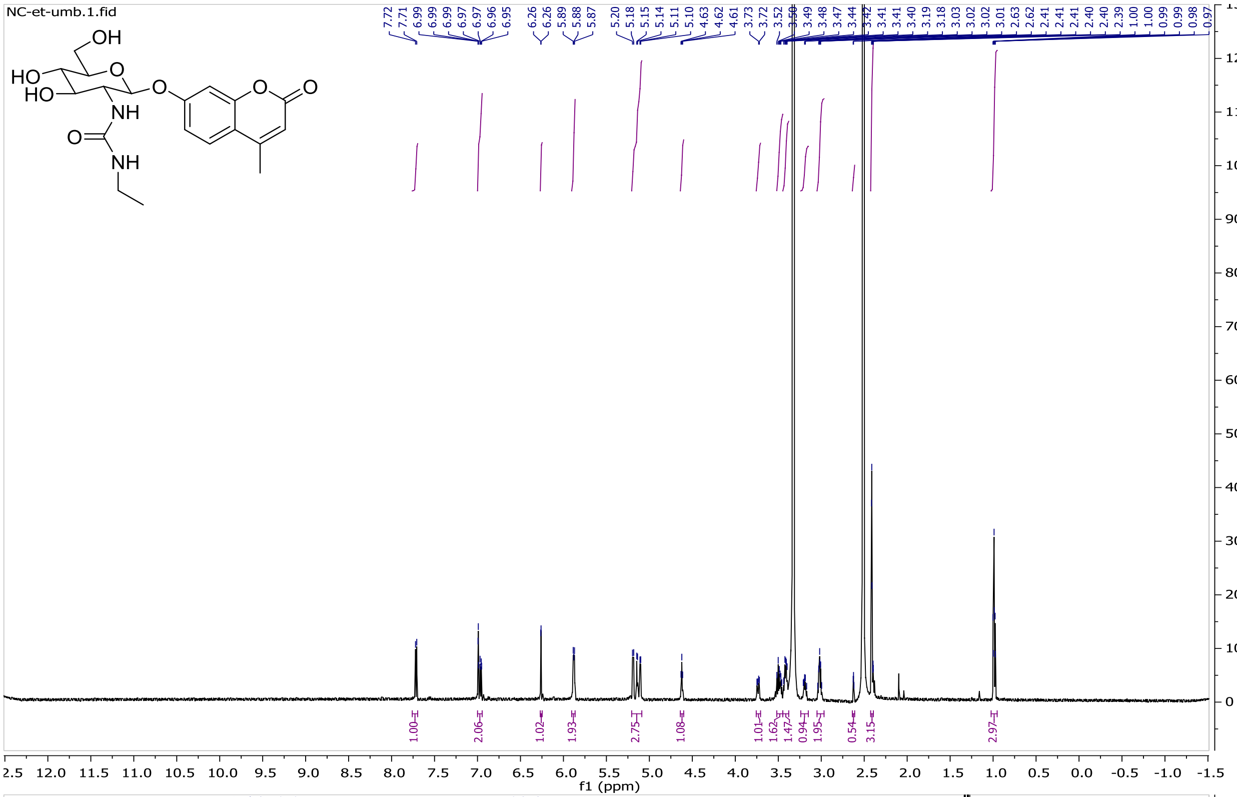
NC-et-umb.1.fid
f1 (ppm)

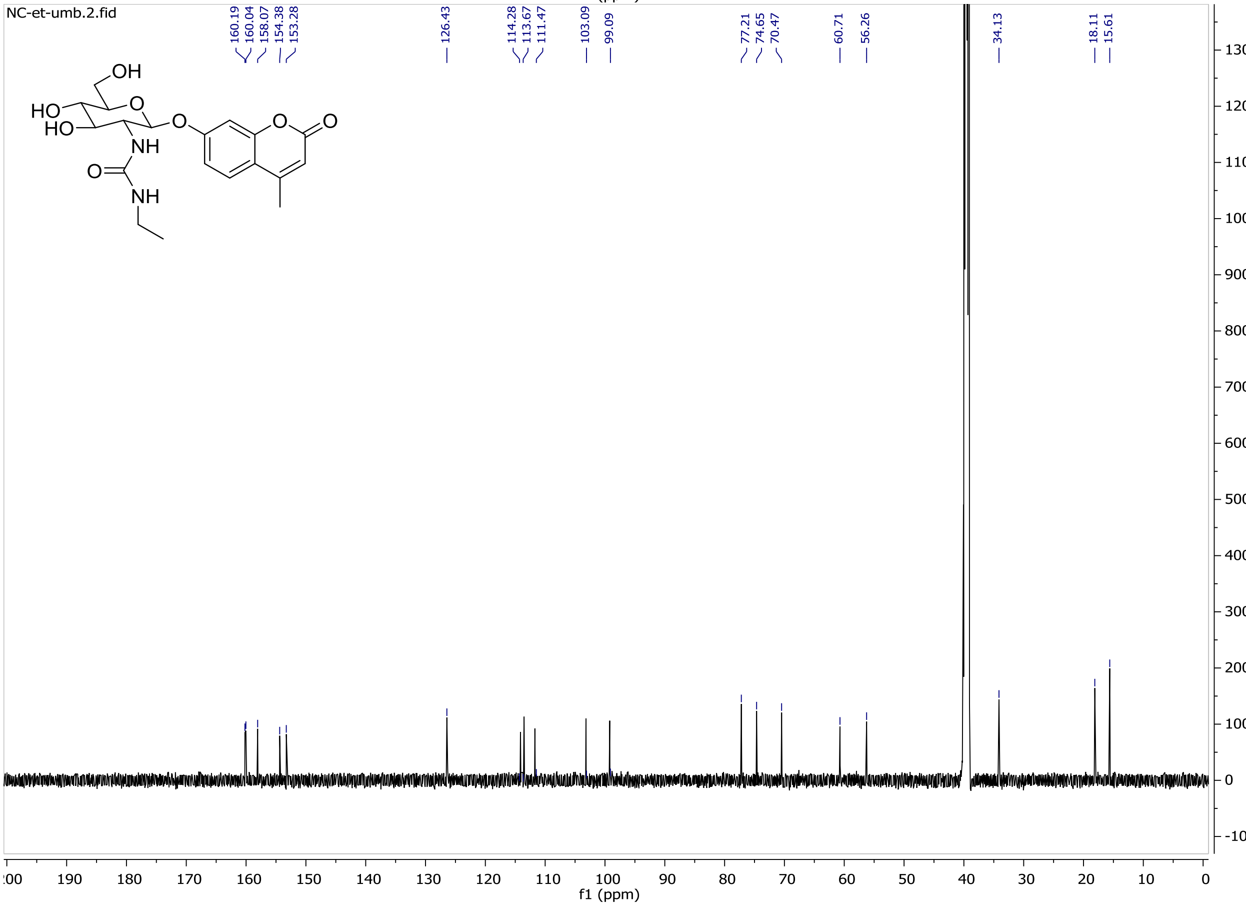
NC-et-umb.2.fid
160.19
160.04
158.07
154.38
153.28
126.43
114.28
113.67
111.47
103.09
99.09
77.21
74.65
70.47
60.71
56.26
34.13
18.11
15.61
f1 (ppm)

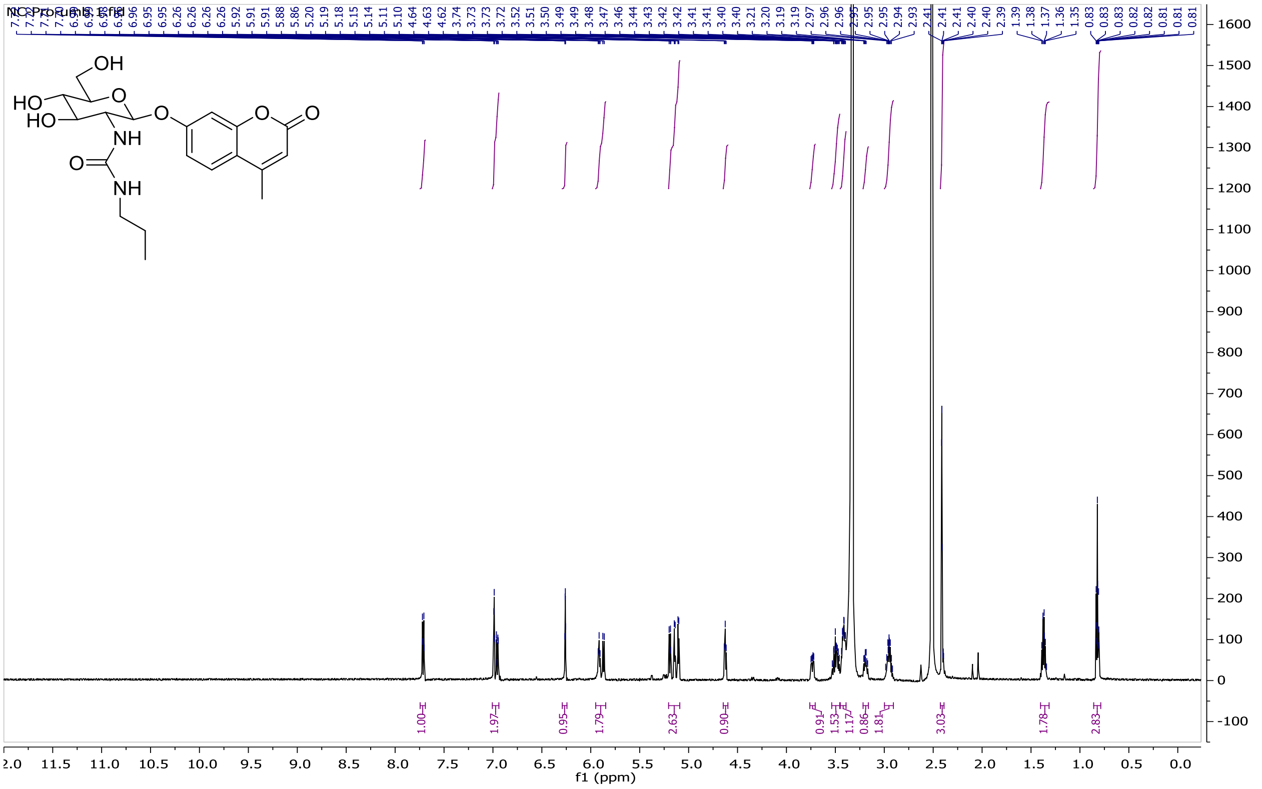
OH
HO
HO
O
O
NH
O
NH
O
O
f1 (ppm)

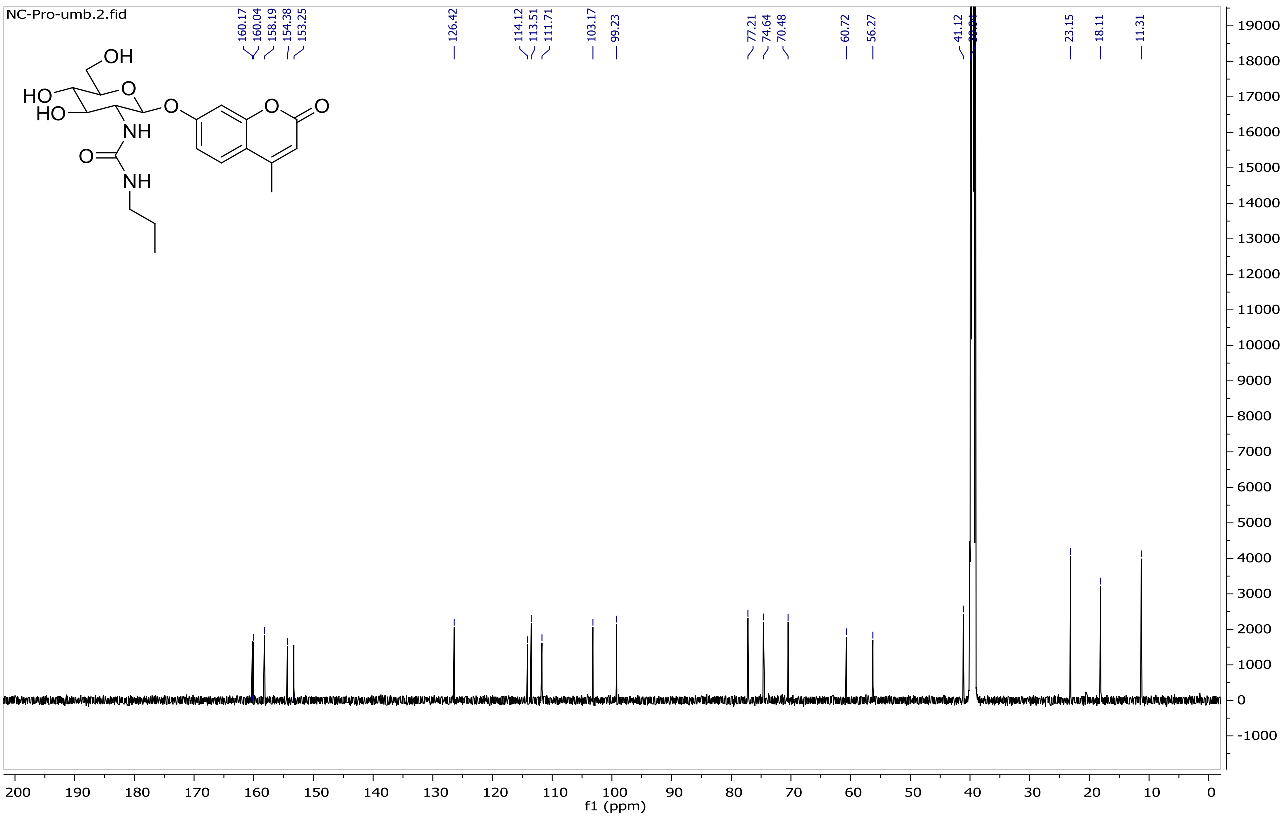
NC-Pro-umb.2.fid
160.17
160.04
158.19
154.38
153.25
126.42
114.12
113.51
111.71
103.17
99.23
77.21
74.64
70.48
60.72
56.27
41.12
23.15
18.11
11.31
OH
HO
HO
O
O
NH
O
NH
O
O
f1 (ppm)

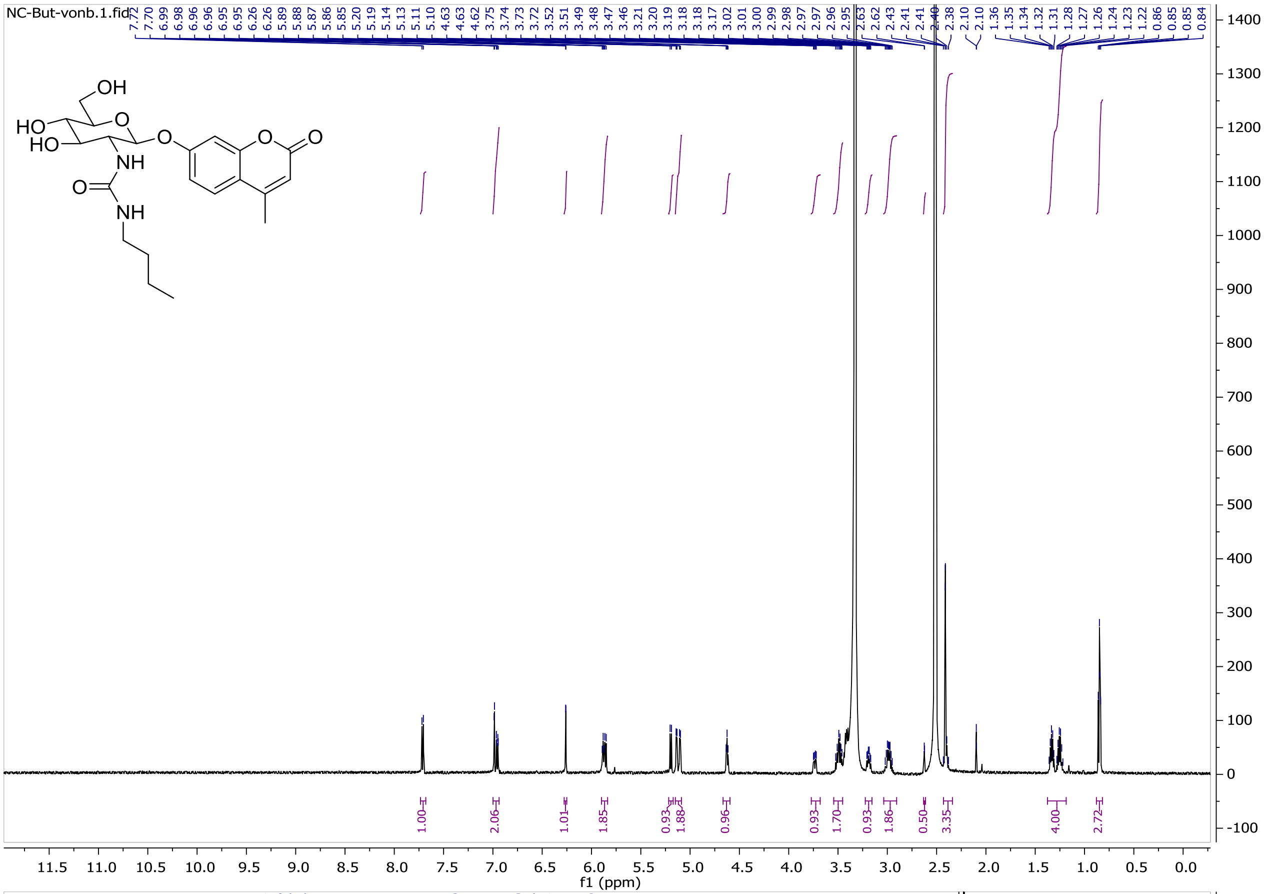

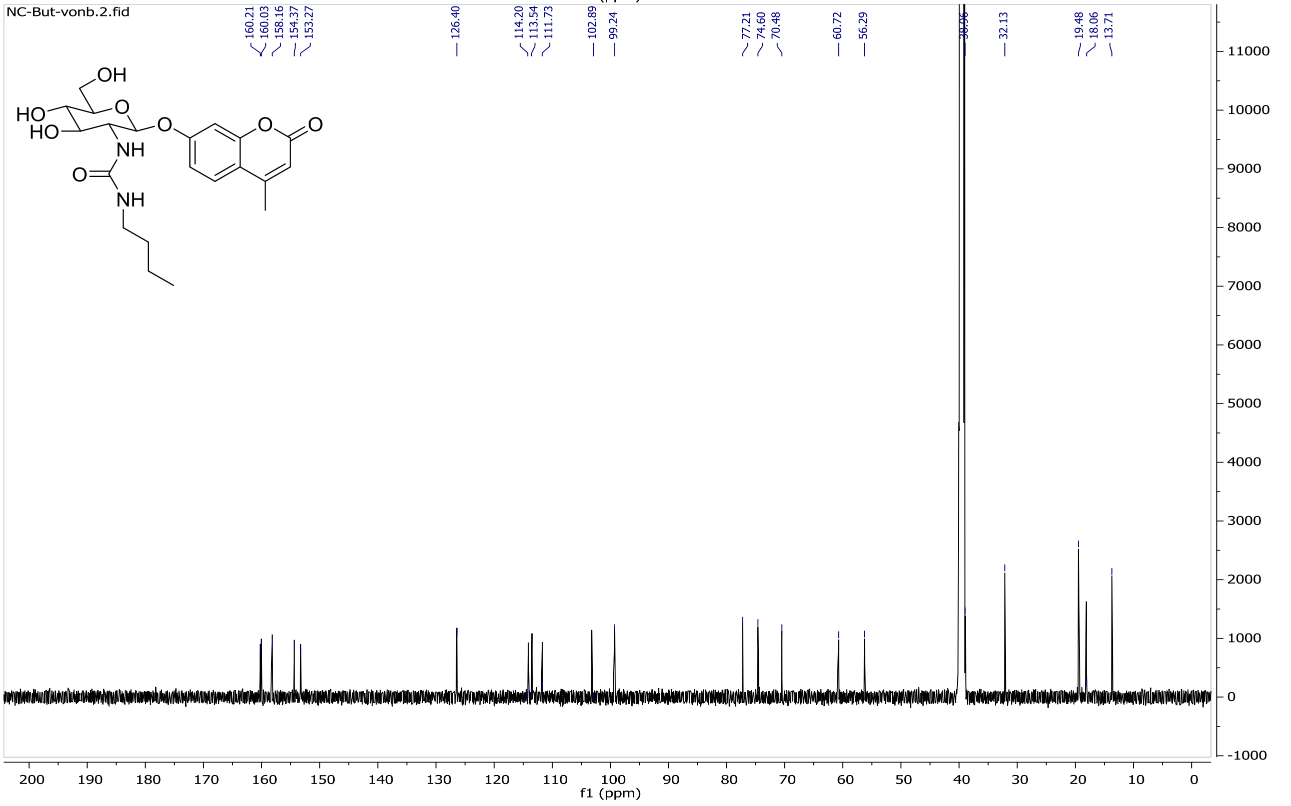

References:

1. Avalos Gonzalez, M., *et al*., *Synthesis of 1,3,4,6-tetra-O-acetyl-2-[3-alkyl-(aryl)-thioreido]-2-deoxy-α-D-glucopyranoses and their transformation into 2-alkyl(aryl)amino-(1,2-dideoxy-α-D-glucopyrano)[2,1-d]-2-thiazolines.* Carb Res, 1986. **154**(1): p. 49-62.
2. Yuzwa, S.A. *et.al.*, *A potent mechanism-inspired O-GlcNAcase inhibitor that blocks phosphorylation of tau in vivo.* Nat Chem Biol, 2008. **4**(8): p. 483-90.
3. Roeser, K.R. and Legler, G., *Role of sugar hydroxyl groups in glycoside hydrolysis. Cleavage mechanism of deoxyglucosides and related substrates by β-glucosidase A3 from Aspergillus wentii.* Bioch et Biophys Acta (BBA) - Enzymology, 1981. **657**(2): p. 321-333.
4. Macauley, M.S., *et al., O-GlcNAcase uses substrate-assisted catalysis: kinetic analysis and development of highly selective mechanism-inspired inhibitors.* J Biol Chem, 2005. **280**(27): p. 25313-22.
5. Macauley, M.S., Stubbs, K.A. and Vocadlo, D.J., *O-GlcNacase Catalyzes Cleavage of Thioglycosides without General Acid Catalysis.* J Am Chem Soc, 2005. **127**(49): p. 17202-17203.
6. Perrin, L.C. and Fabian, M.A., *Multicomponent NMR Titration for Simultaneous Measurement of Relative pK_a's.* Anal Chem, 1996. **68**: p. 2127-2134.
7. Rabenstein, D.L. and Mariappan, S.V.S., *Determination of ^{15}N Isotope Effects on the Acid-Base Equilibria of Amino Groups in Amino Acids by ^{13}C NMR.* J Org Chem, 1993. **58**: p. 4487-4489.
8. Roses, M. *et.al.*, *Dissociation constants of phenols in methanol-water mixtures.* J Chromatograph A, 2000. **867**: p. 45-56.
9. Dennis, R.J., *et al*., *Structure and mechanism of a bacterial β-glucosaminidase having O-GlcNAcase activity.* Nat Struct Mol Biol, 2006. **13**(4): p. 365-71.
10. Kabsch, W., *Xds.* Acta Crystallogr D Biol Crystallogr, 2010. **66**(Pt 2): p. 125-32.
11. Evans, P., *Scaling and assessment of data quality.* Acta Crystallogr D Biol Crystallogr, 2006. **62**(Pt 1): p. 72-82.
12. Leslie, A.W. and Powell, H., *Processing diffraction data with mosflm*, in *Evolving Methods for Macromolecular Crystallography*, R. Read and J. Sussman, Editors. 2007, Springer Netherlands. p. 41-51.
13. Evans, P.R. and Murshudov, G.N., *How good are my data and what is the resolution?* Acta Crystallogr D Biol Crystallogr, 2013. **69**(Pt 7): p. 1204-14.
14. Winn, M.D. *et al., Ongoing developments in CCP4 for high-throughput structure determination.* Acta Crystallogr D Biol Crystallogr, 2002. **58**: p. 1929-1936.
15. Murshudov, G.N., *et al., REFMAC5 for the refinement of macromolecular crystal structures.* Acta Crystallogr D Biol Crystallogr, 2011. **67**(Pt 4): p. 355-67.
16. Adams, P.D., *et al.*, *The Phenix software for automated determination of macromolecular structures.* (1095-9130 (Electronic)).
17. Emsley, P., *et al., Features and development of Coot.* Acta Crystallographica Section D, 2010. **66**(4): p. 486-501.
18. Lebedev, A.A., *et al.*, *JLigand: a graphical tool for the CCP4 template-restraint library.* Acta Crystallogr D Biol Crystallogr, 2012. **68**(4): p. 431-440.
19. Chen, V.B., *et al., MolProbity: all-atom structure validation for macromolecular crystallography.* Acta Crystallogr D Biol Crystallogr, 2010. **66**(1): p. 12-21.
20. McNicholas, S., Potterton, E., Wilson, K.S. and Noble, M.E.M., *Presenting your structures: the CCP4mg molecular-graphics software.* Acta Crystallogr D Biol Crystallogr, 2011. **67**: p. 386-394.
